# Supplementary figures and images for: Inhibition of DNMT1 methyltransferase activity via glucose-regulated O-GlcNAcylation alters the epigenome (part 1 of 2)
Source: eLife. 2023 Jul 20;12:e85595. doi: 10.7554/eLife.85595 (PMC10390045; doi:10.7554/eLife.85595)

Figure 1D

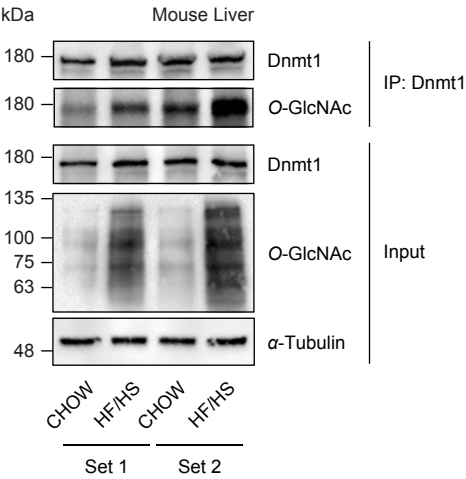

IP: Dnmt1

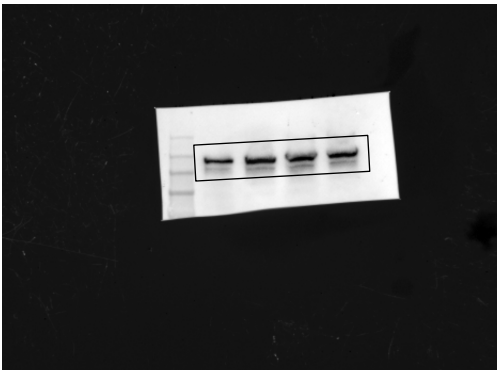

IP: O-GlcNAc

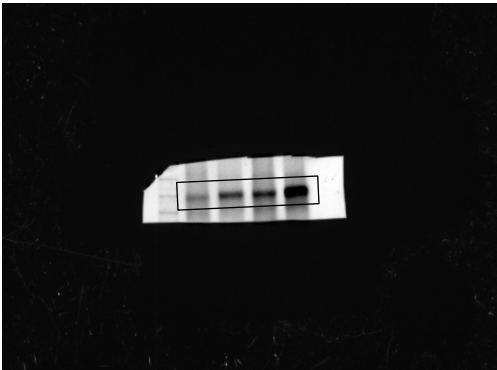

Input: Dnmt1

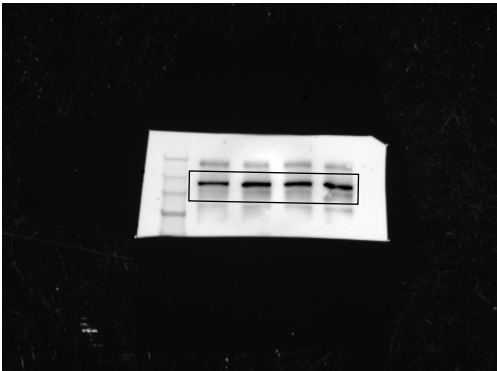

Input: O-GlcNAc

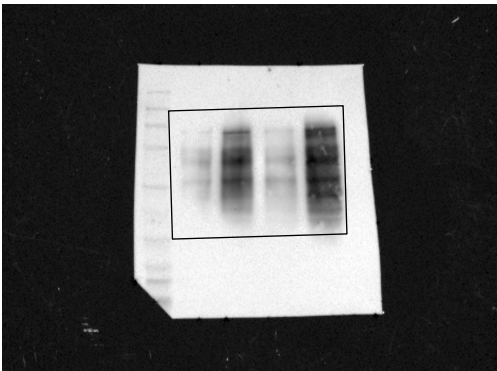

Input:  $\alpha$ -Tubulin

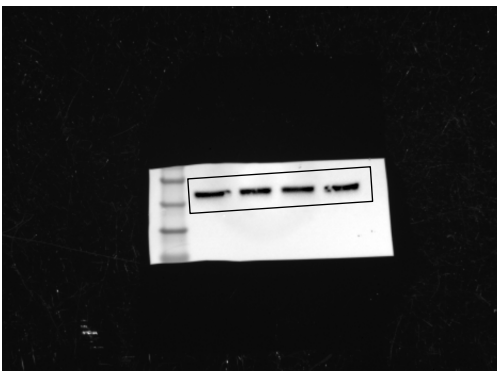

Supplement: Figure 1—source data 1. [file elife-85595-fig1-data1.zip › Figure 1-source data 1/Labeled_file/Figure 1D-source data 1.pdf]

Figure 1A

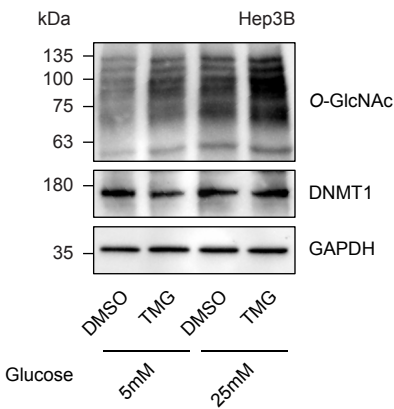

O-GlcNAc

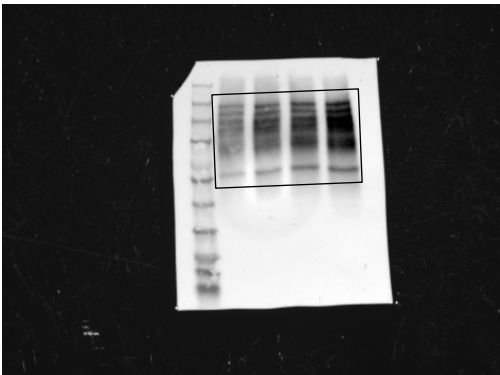

DNMT1

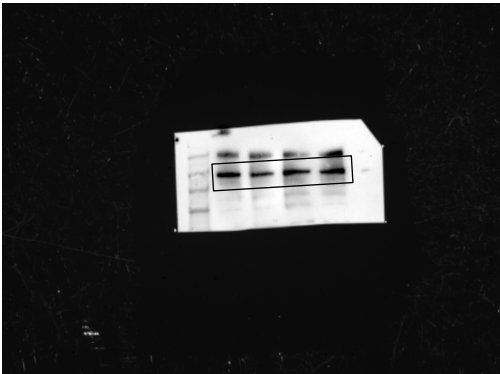

GAPDH

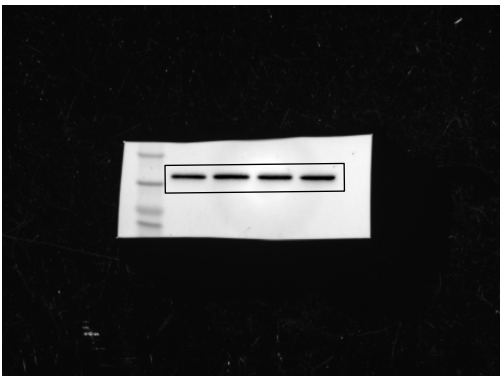

Supplement: Figure 1—source data 1. [file elife-85595-fig1-data1.zip › Figure 1-source data 1/Labeled_file/Figure 1A-source data 1.pdf]

Figure 1B

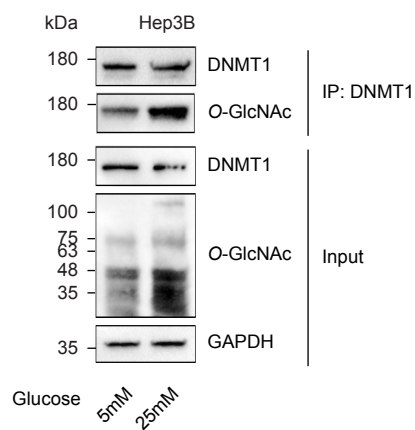

IP: DNMT1

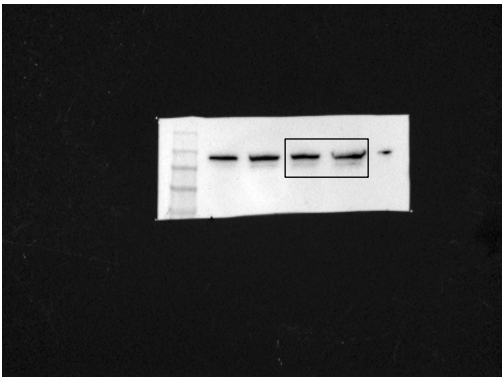

IP: O-GlcNAc

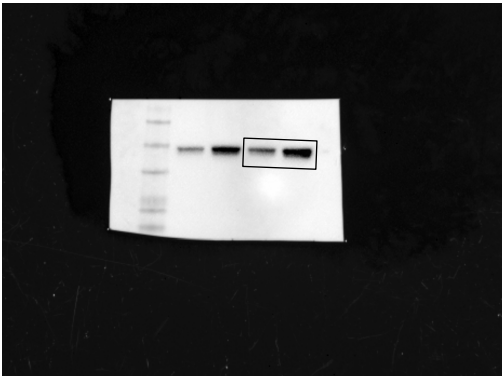

Input: DNMT1

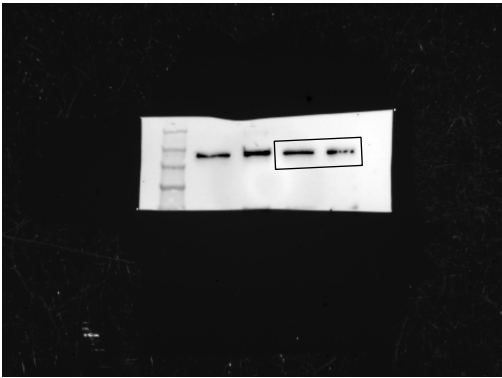

Input: O-GlcNAc

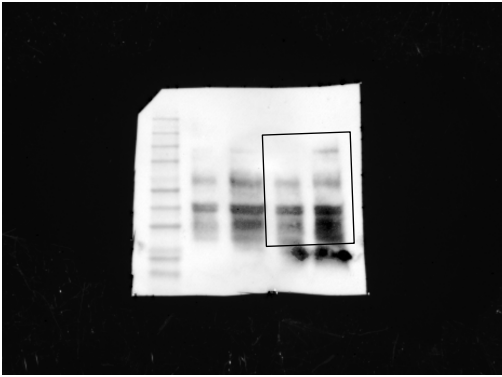

Input: GAPDH

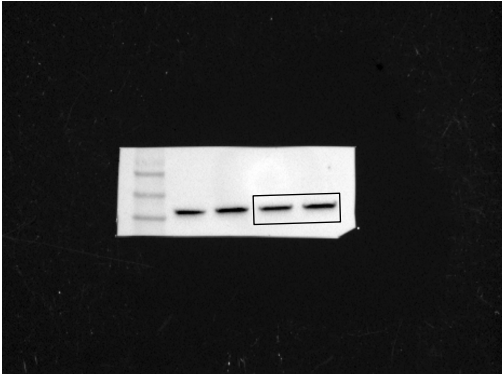

Supplement: Figure 1—source data 1. [file elife-85595-fig1-data1.zip › Figure 1-source data 1/Labeled_file/Figure 1B-source data 1.pdf]

Figure 1C

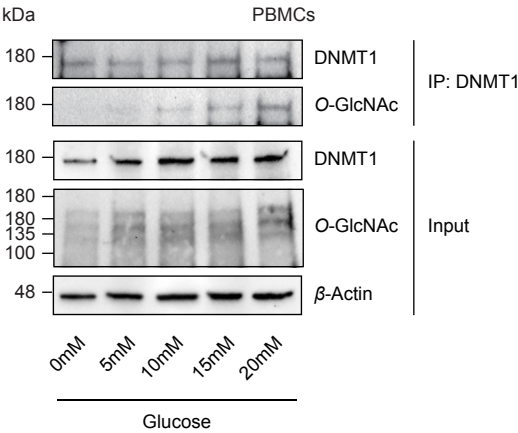

IP: DNMT1

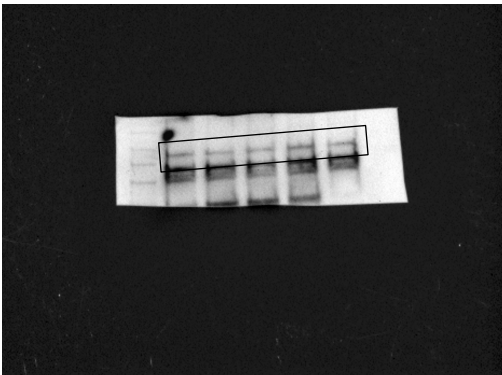

IP: O-GlcNAc

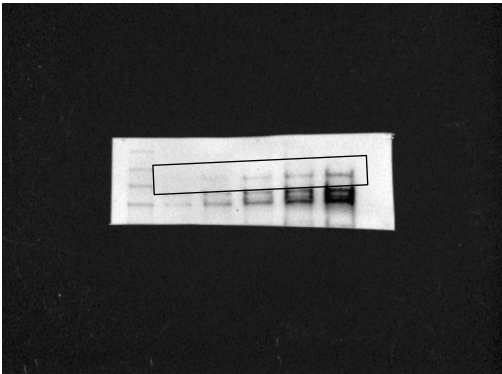

Input: DNMT1

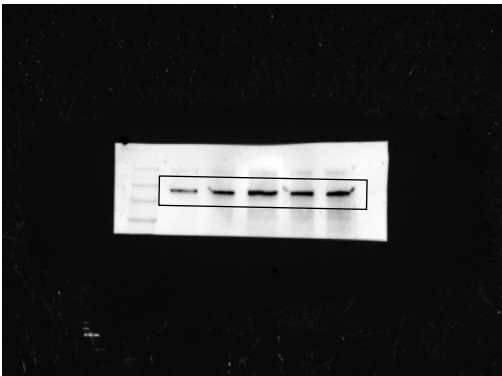

Input: O-GlcNAc

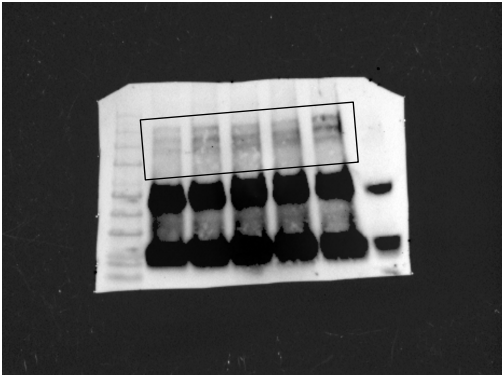

Input:  $\beta$ -Actin

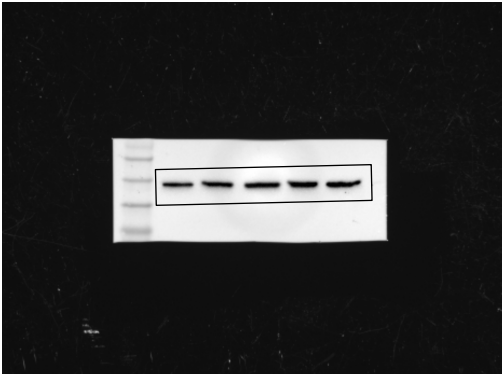

Supplement: Figure 1—source data 1. [file elife-85595-fig1-data1.zip › Figure 1-source data 1/Labeled_file/Figure 1C-source data 1.pdf]

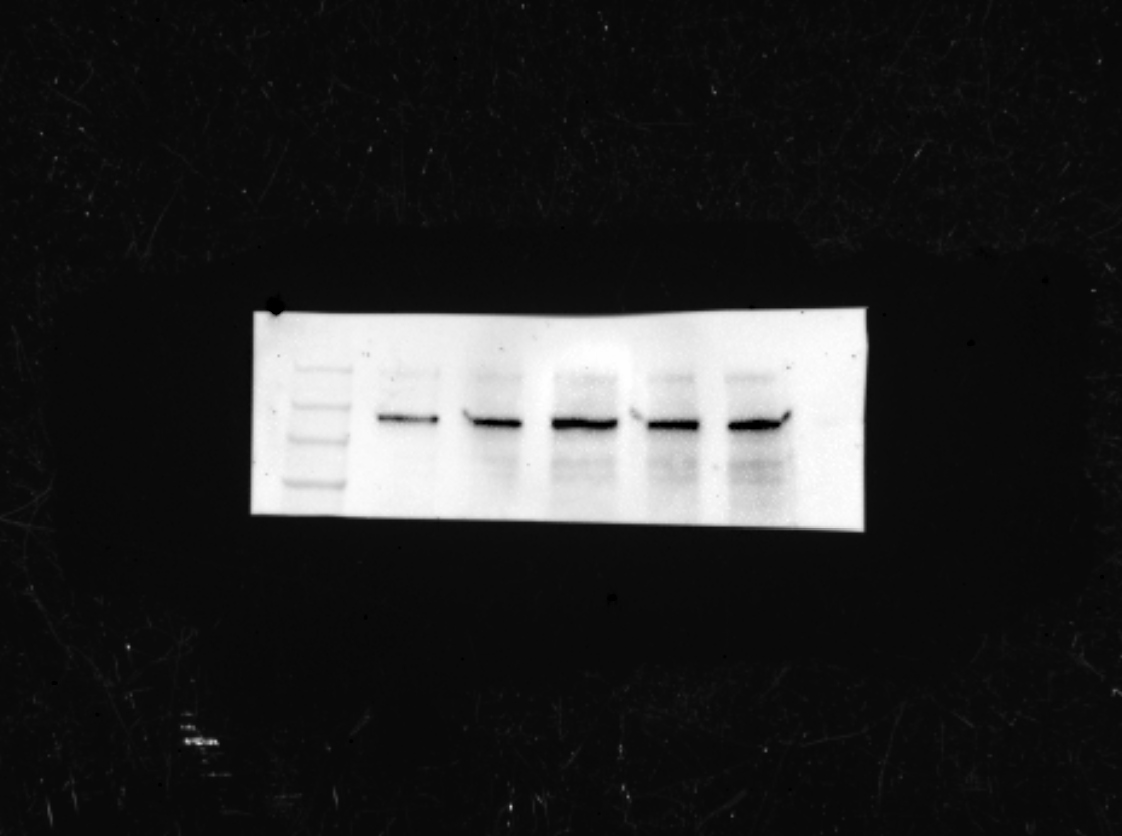

Supplement: Figure 1—source data 1. [file elife-85595-fig1-data1.zip › Figure 1-source data 1/Original_files/Figure 1C_DNMT1_input.tif]

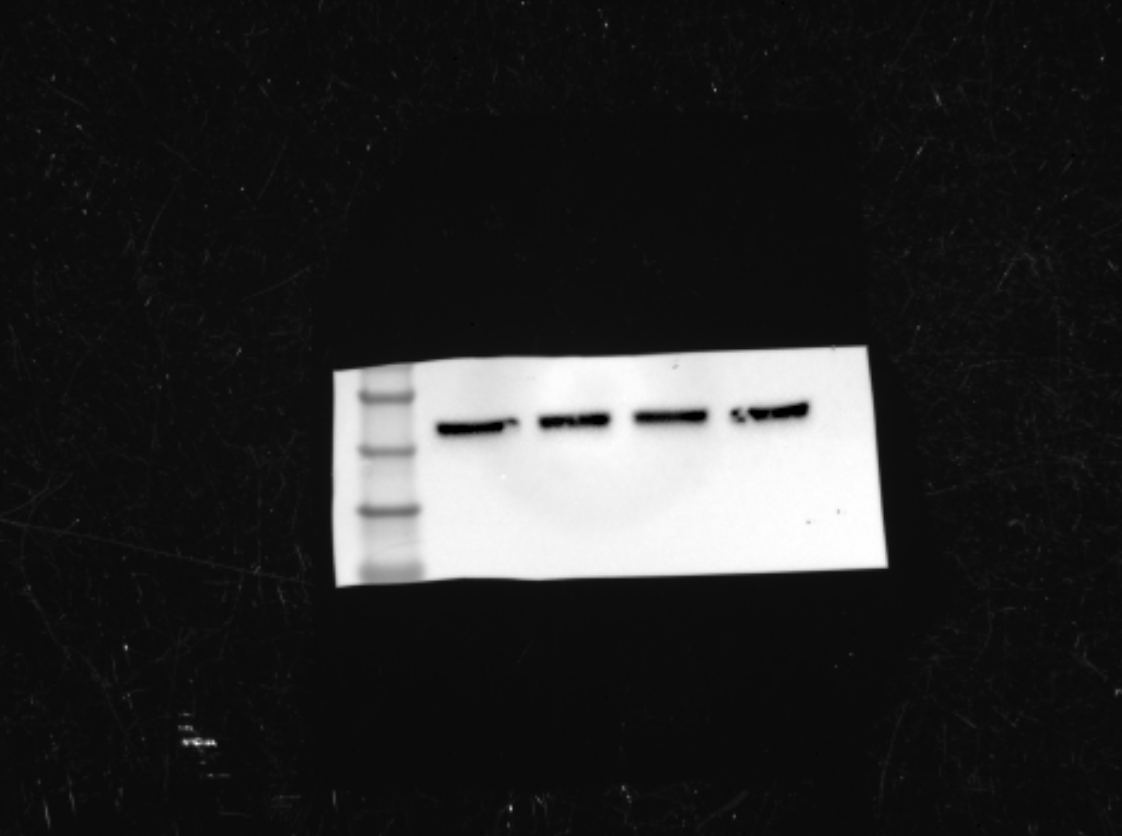

Supplement: Figure 1—source data 1. [file elife-85595-fig1-data1.zip › Figure 1-source data 1/Original_files/Figure 1D_Tubulin_input.tif]

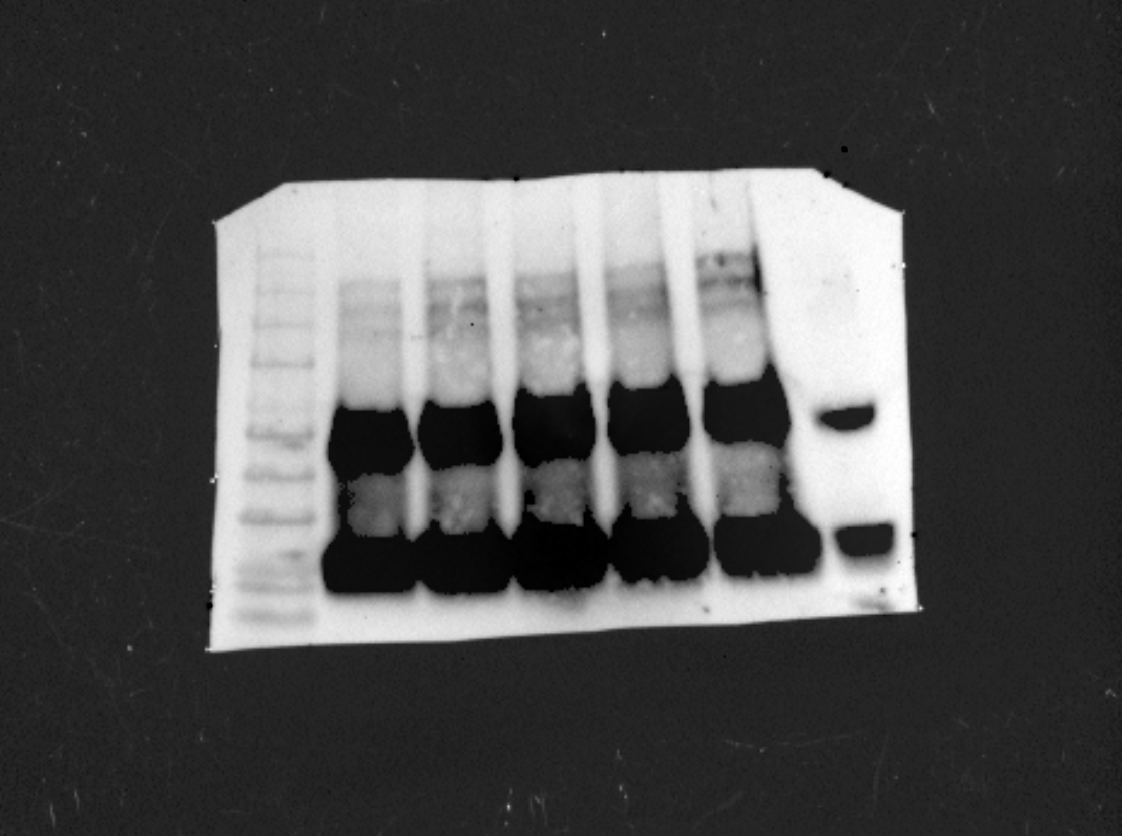

Supplement: Figure 1—source data 1. [file elife-85595-fig1-data1.zip › Figure 1-source data 1/Original_files/Figure 1C_O-GlcNAc_input.tif]

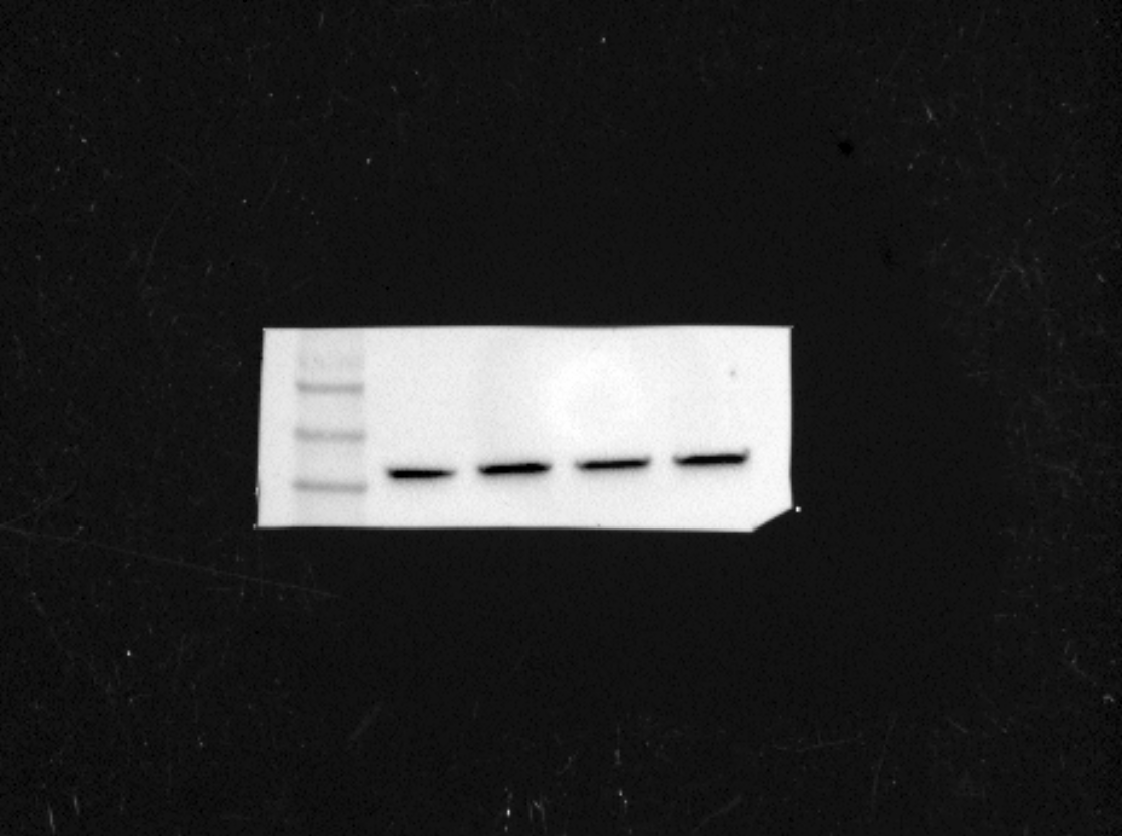

Supplement: Figure 1—source data 1. [file elife-85595-fig1-data1.zip › Figure 1-source data 1/Original_files/Figure 1B_GAPDH_input.tif]

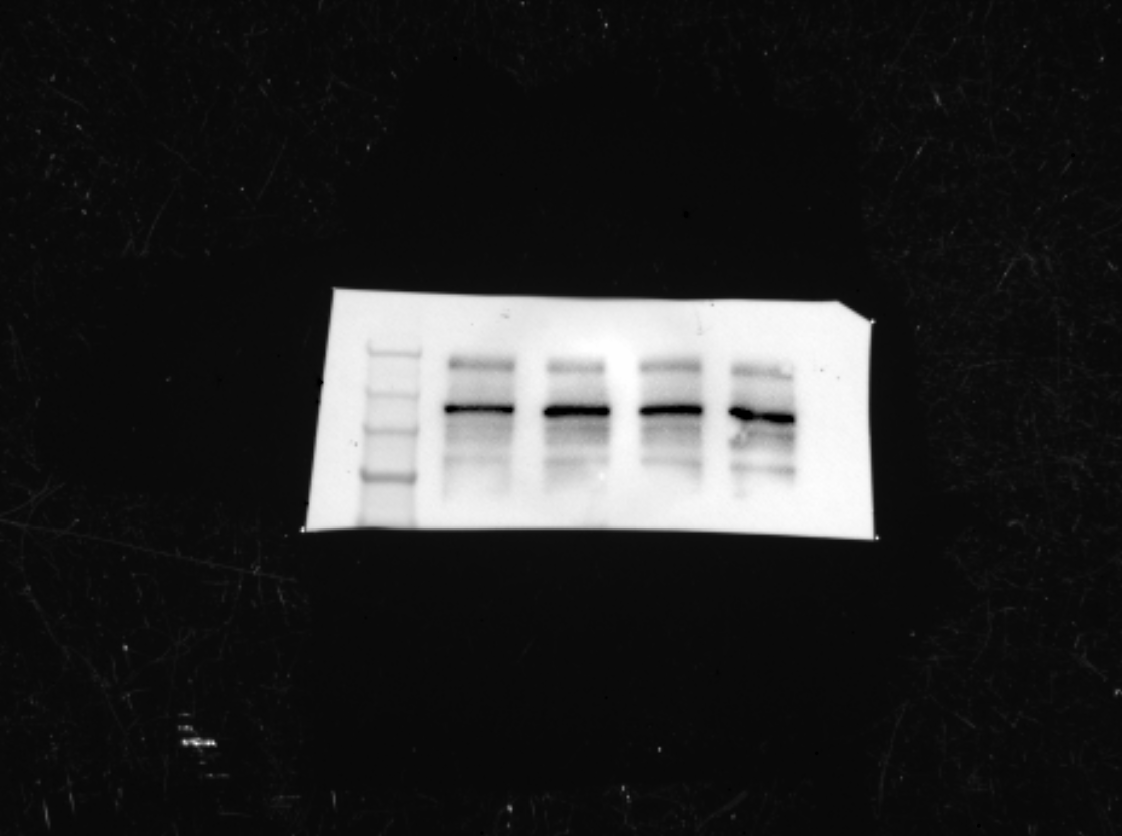

Supplement: Figure 1—source data 1. [file elife-85595-fig1-data1.zip › Figure 1-source data 1/Original_files/Figure 1D_Dnmt1_input.tif]

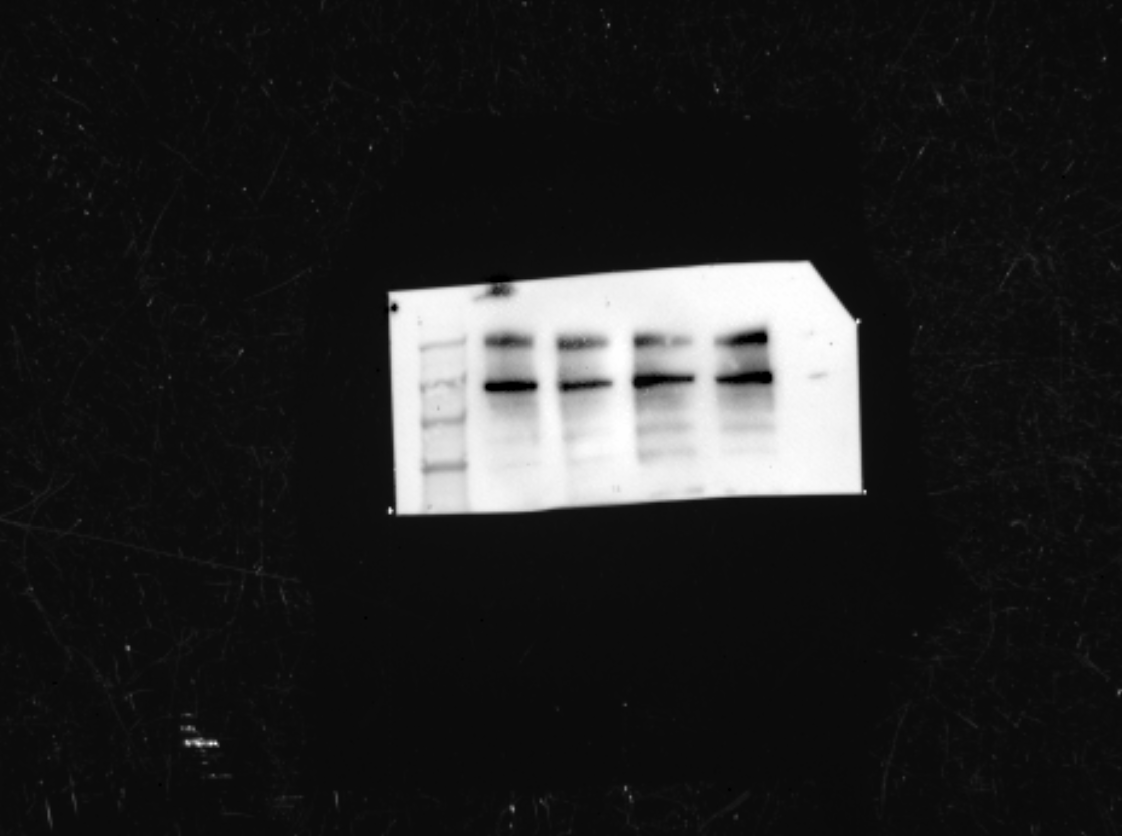

Supplement: Figure 1—source data 1. [file elife-85595-fig1-data1.zip › Figure 1-source data 1/Original_files/Figure 1A_DNMT1.tif]

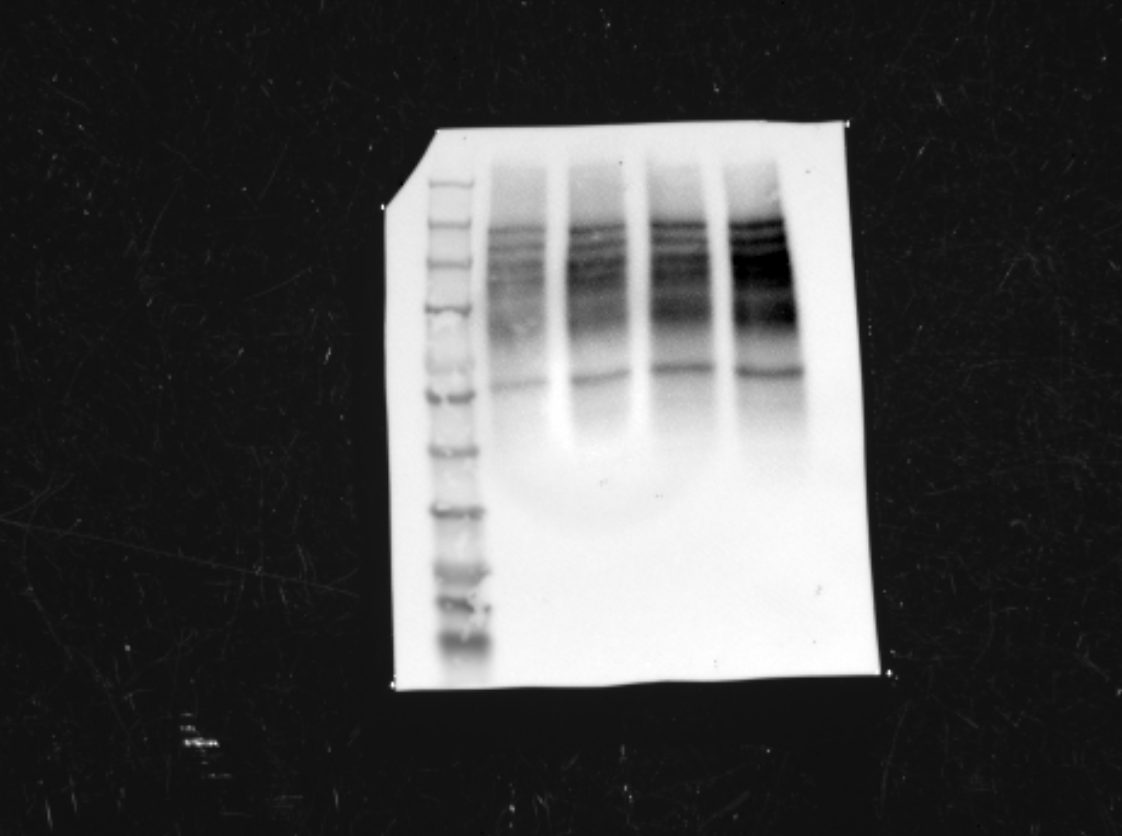

Supplement: Figure 1—source data 1. [file elife-85595-fig1-data1.zip › Figure 1-source data 1/Original_files/Figure 1A_O-GlcNAc.tif]

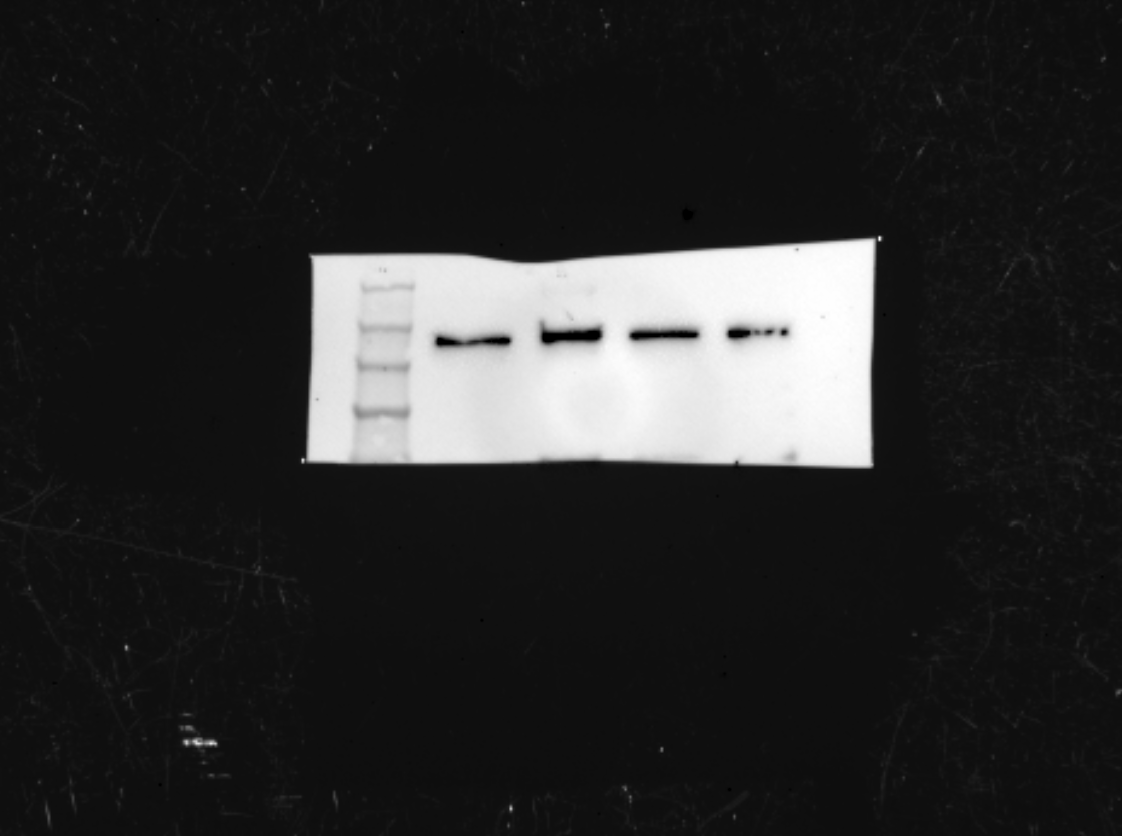

Supplement: Figure 1—source data 1. [file elife-85595-fig1-data1.zip › Figure 1-source data 1/Original_files/Figure 1B_DNMT1_input.tif]

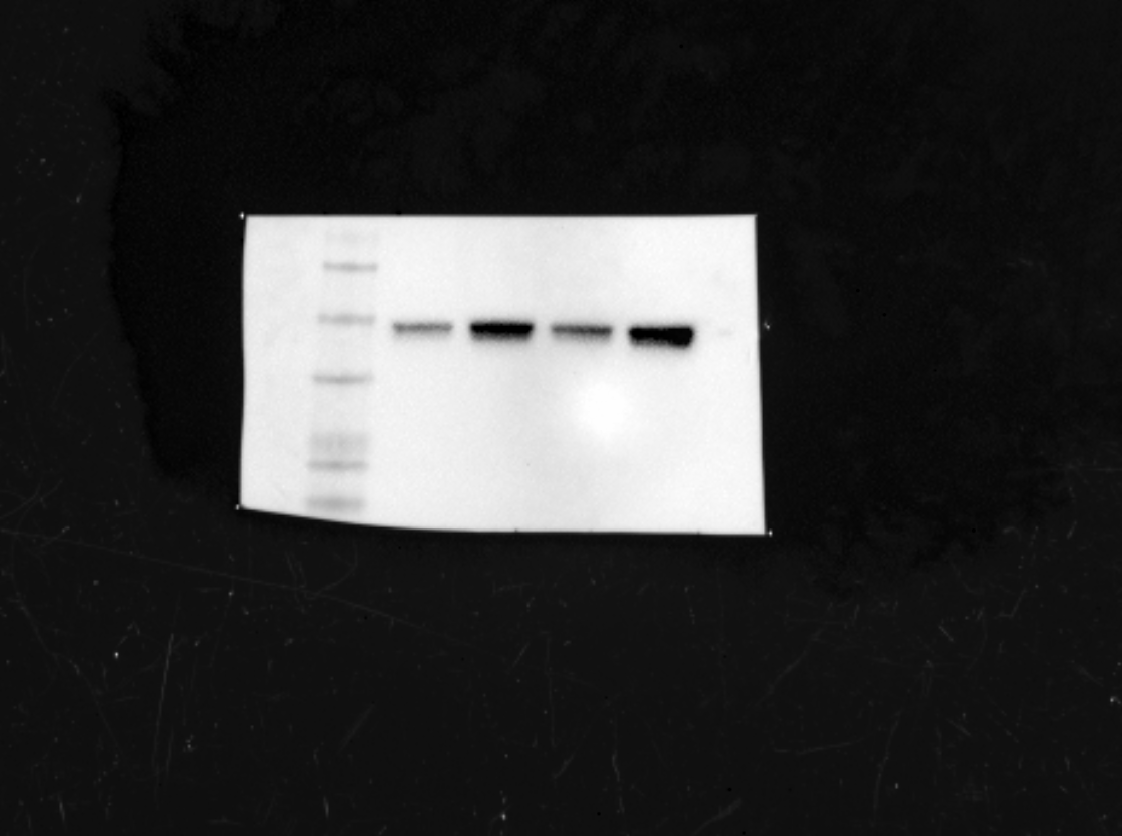

Supplement: Figure 1—source data 1. [file elife-85595-fig1-data1.zip › Figure 1-source data 1/Original_files/Figure 1B_O-GlcNAc_IP.tif]

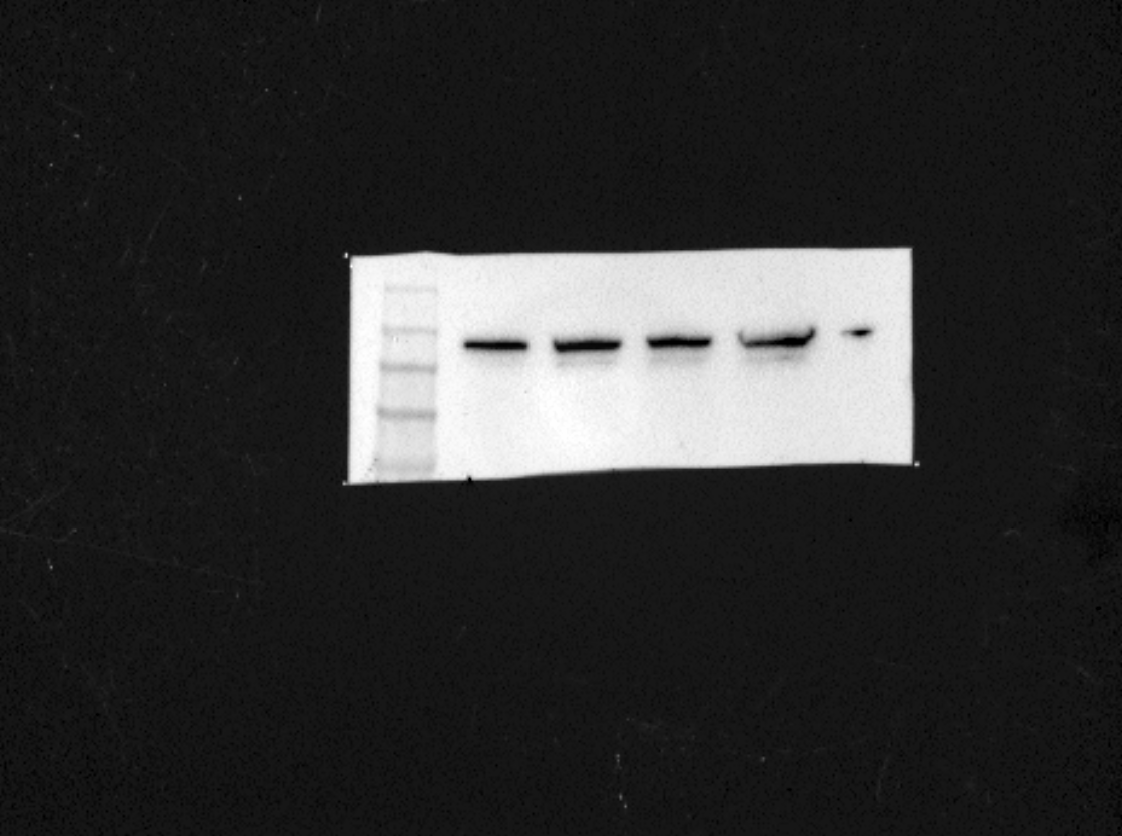

Supplement: Figure 1—source data 1. [file elife-85595-fig1-data1.zip › Figure 1-source data 1/Original_files/Figure 1B_DNMT1_IP.tif]

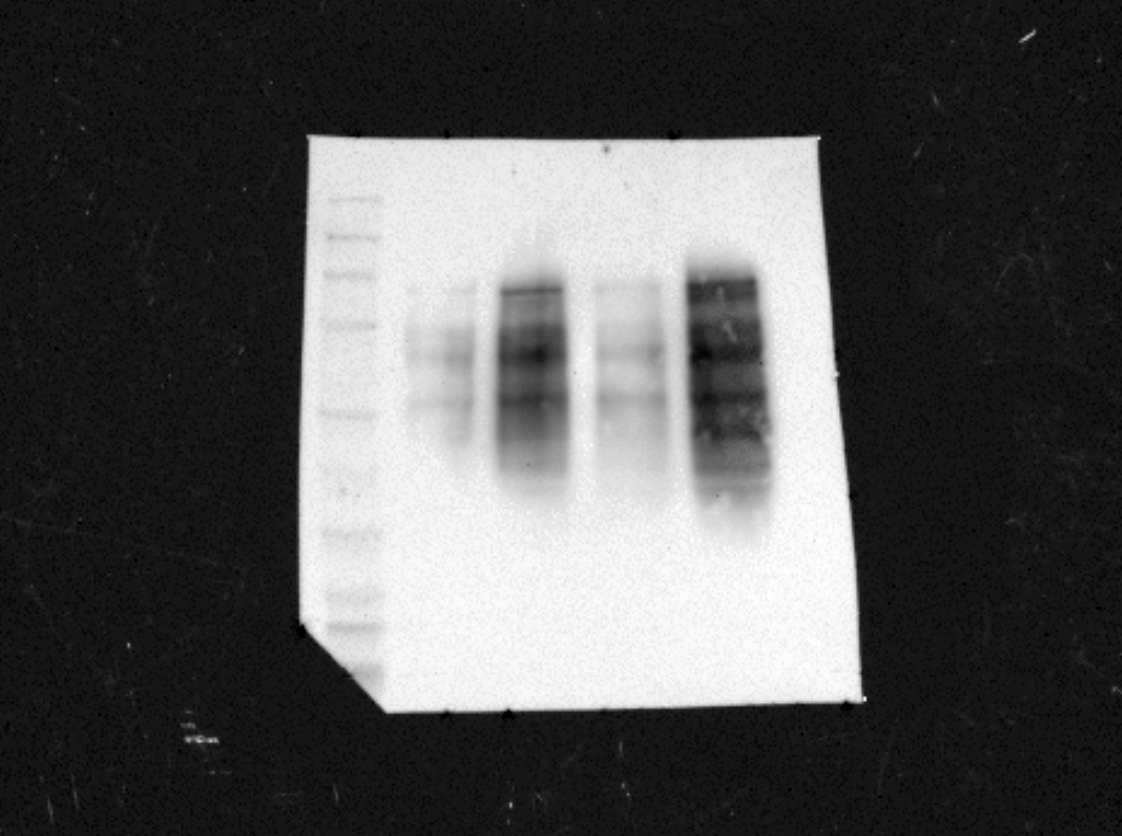

Supplement: Figure 1—source data 1. [file elife-85595-fig1-data1.zip › Figure 1-source data 1/Original_files/Figure 1D_O-GlcNAc_input.tif]

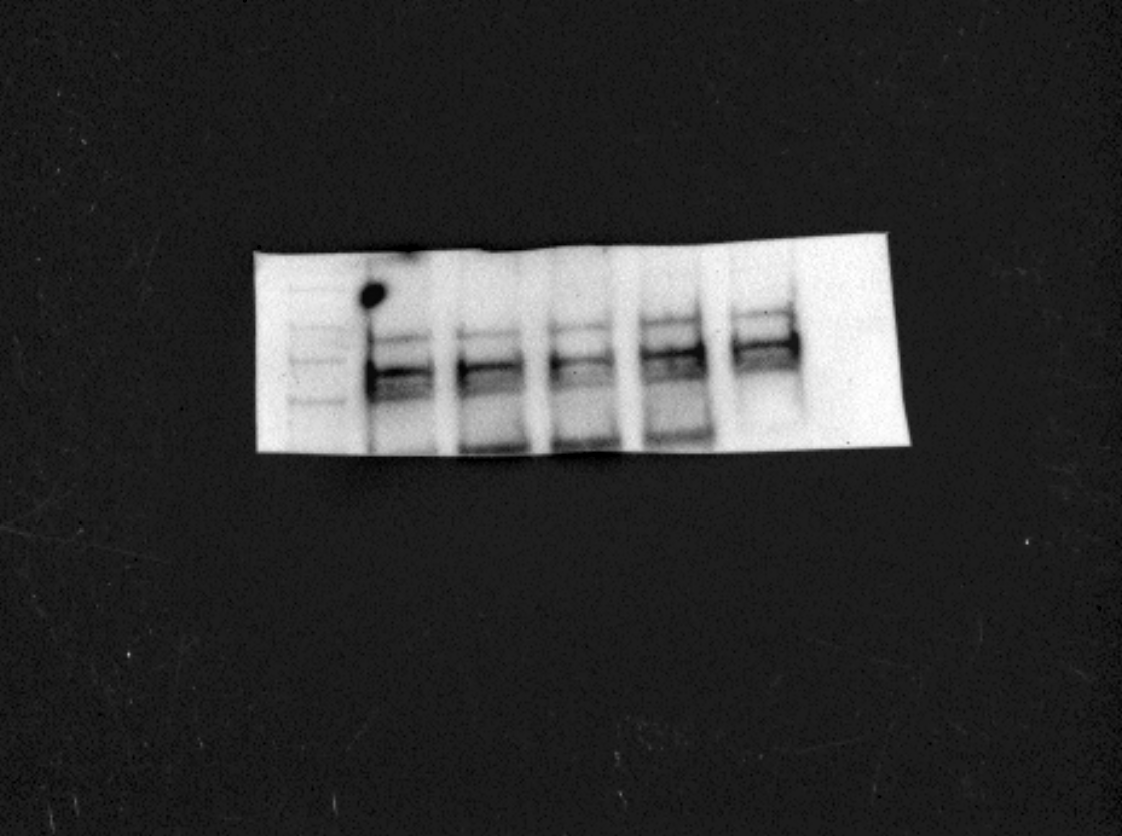

Supplement: Figure 1—source data 1. [file elife-85595-fig1-data1.zip › Figure 1-source data 1/Original_files/Figure 1C_DNMT1_IP.tif]

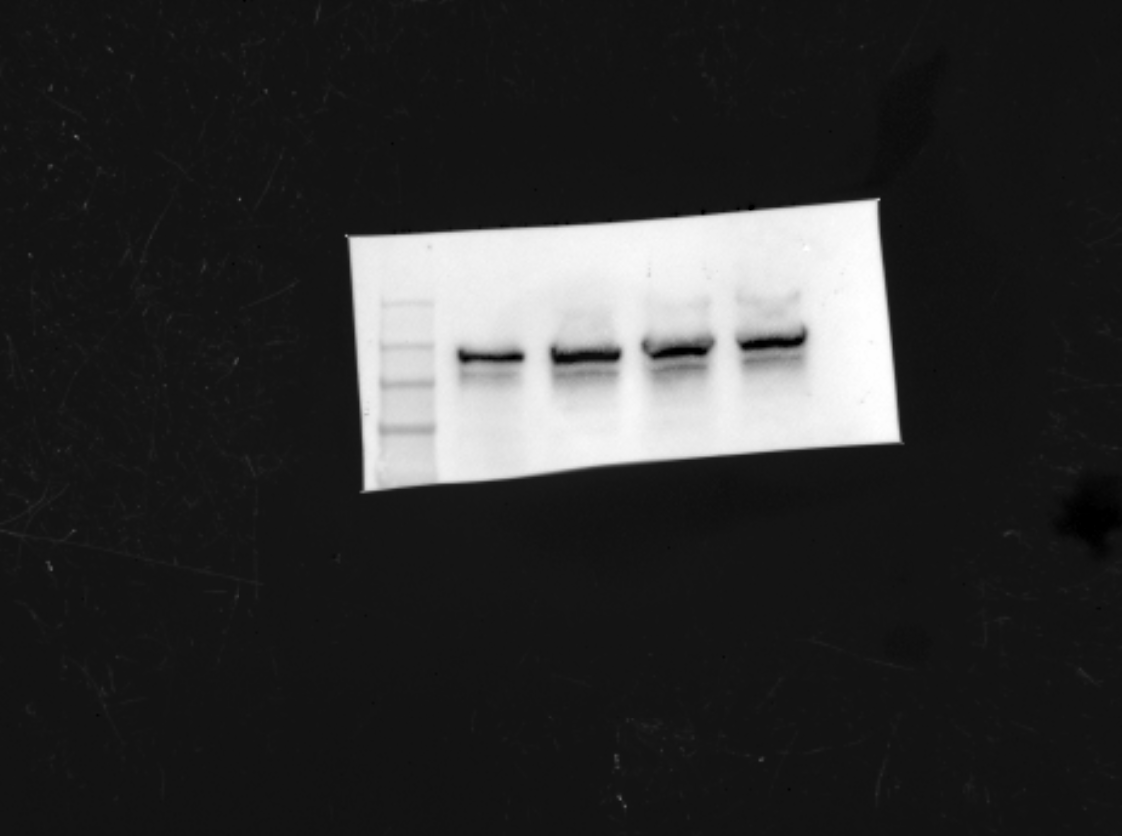

Supplement: Figure 1—source data 1. [file elife-85595-fig1-data1.zip › Figure 1-source data 1/Original_files/Figure 1D_Dnmt1_IP.tif]

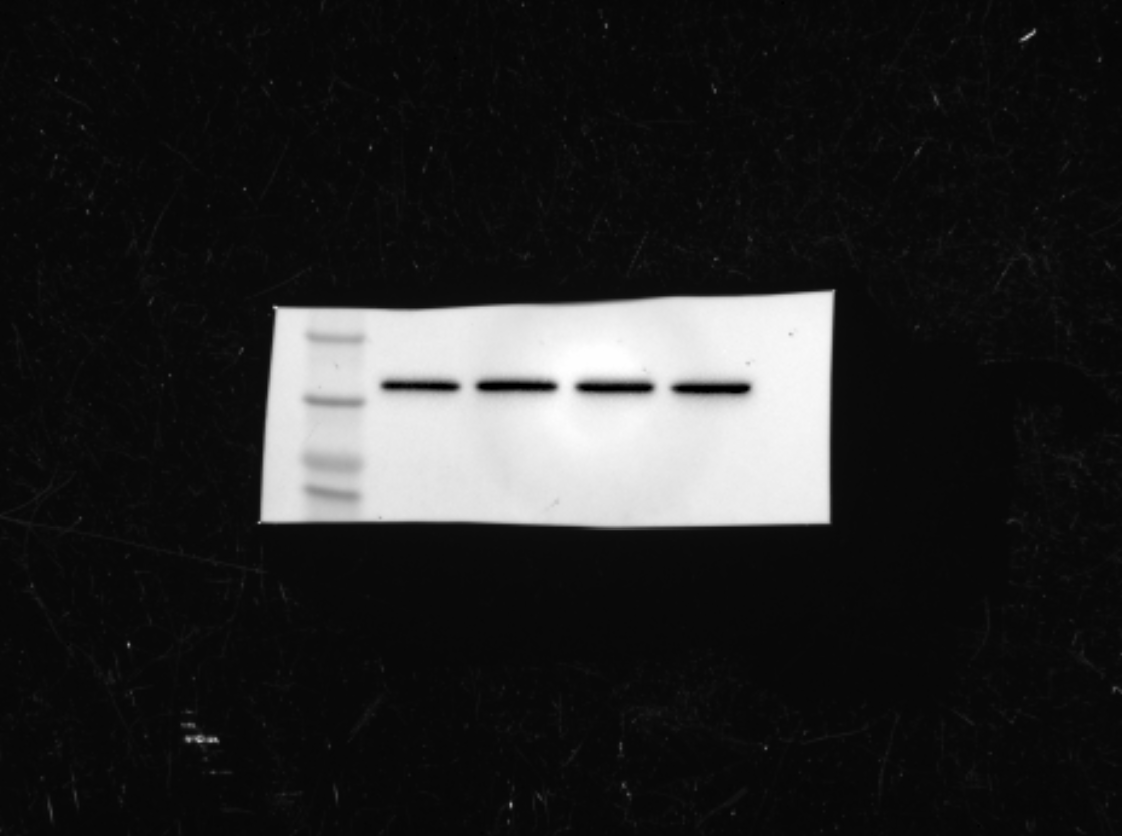

Supplement: Figure 1—source data 1. [file elife-85595-fig1-data1.zip › Figure 1-source data 1/Original_files/Figure 1A_GAPDH.tif]

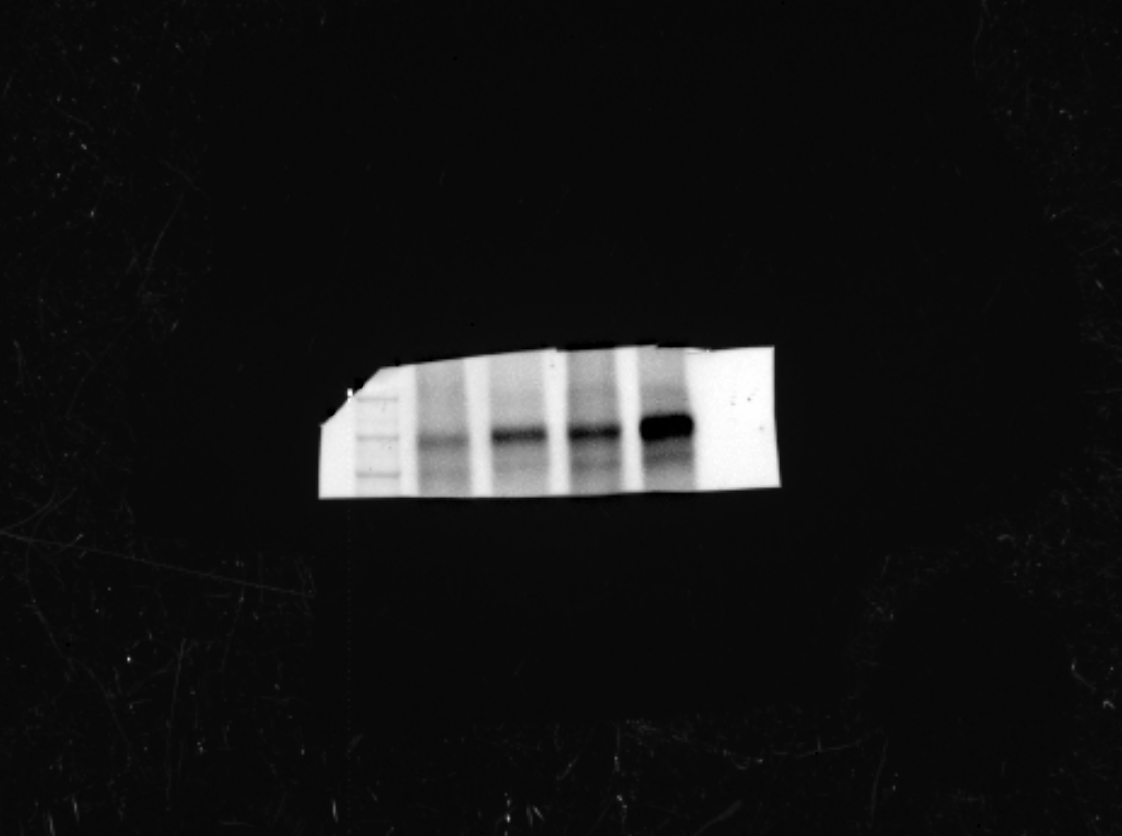

Supplement: Figure 1—source data 1. [file elife-85595-fig1-data1.zip › Figure 1-source data 1/Original_files/Figure 1D_O-GlcNAc_IP.tif]

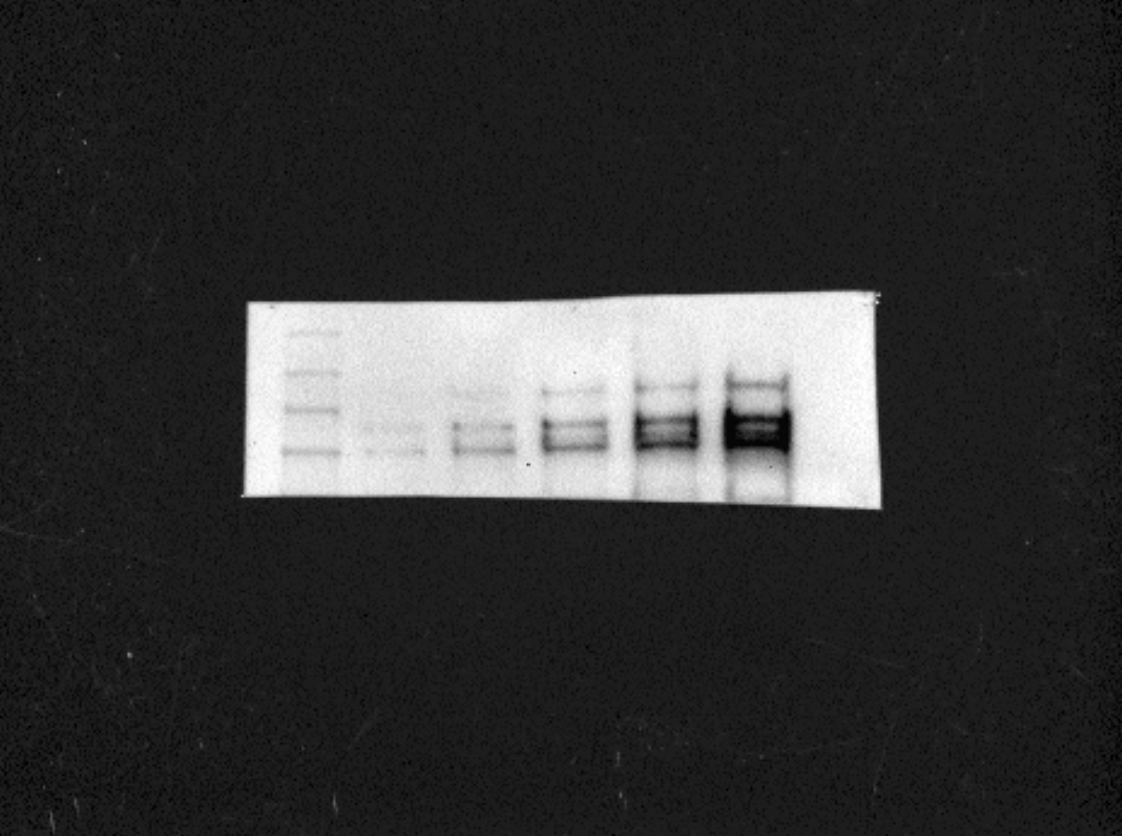

Supplement: Figure 1—source data 1. [file elife-85595-fig1-data1.zip › Figure 1-source data 1/Original_files/Figure 1C_O-GlcNAc_IP.tif]

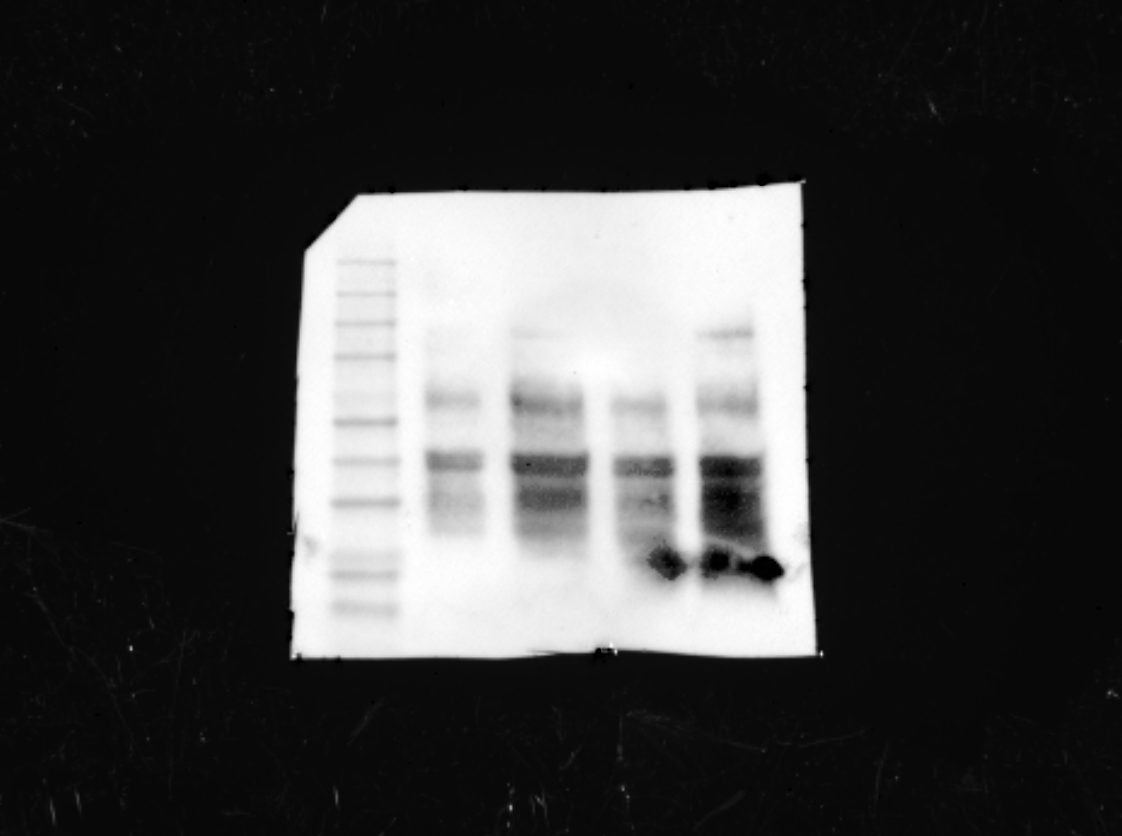

Supplement: Figure 1—source data 1. [file elife-85595-fig1-data1.zip › Figure 1-source data 1/Original_files/Figure 1B_O-GlcNAc_input.tif]

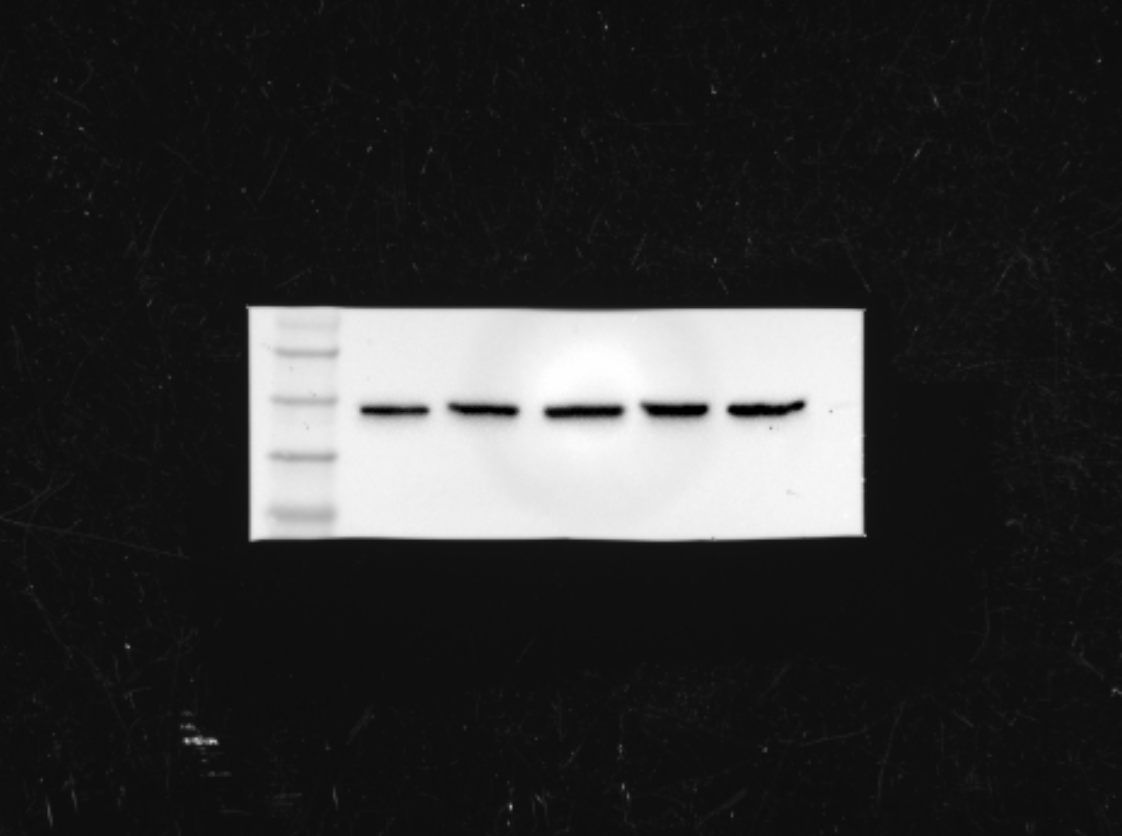

Supplement: Figure 1—source data 1. [file elife-85595-fig1-data1.zip › Figure 1-source data 1/Original_files/Figure 1C_Actin_input.tif]

Figure 1—figure supplement 1

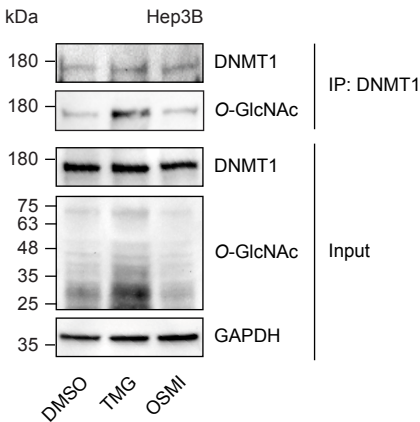

IP: DNMT1

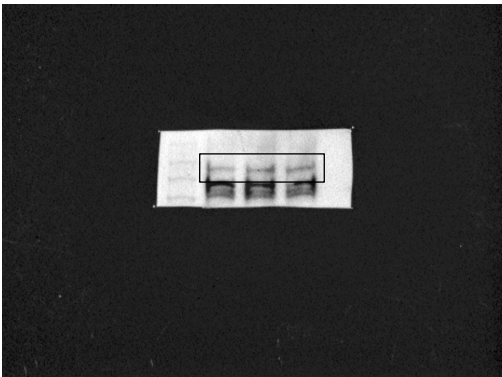

IP: O-GlcNAc

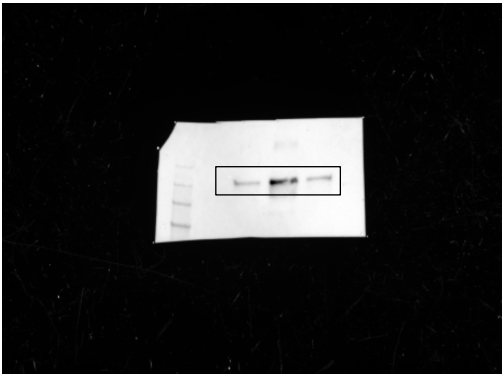

Input: DNMT1

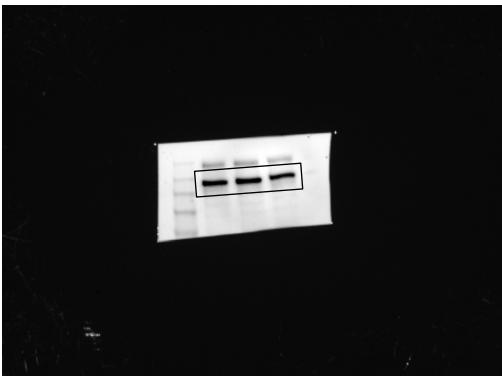

Input: O-GlcNAc

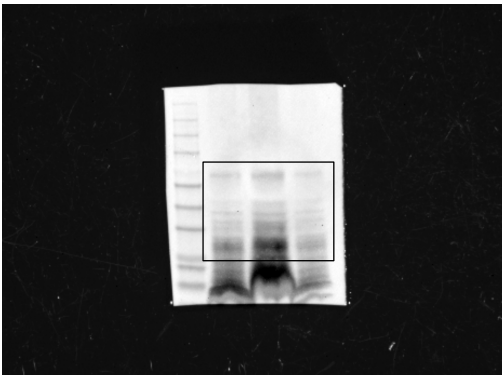

Input: GAPDH

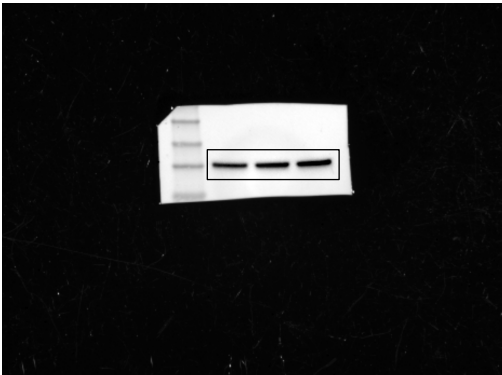

Supplement: Figure 1—figure supplement 1—source data 1. [file elife-85595-fig1-figsupp1-data1.zip › Figure 1-figure supplement 1-source data 1/Labeled_file/Figure 1-figure supplement 1-source data 1.pdf]

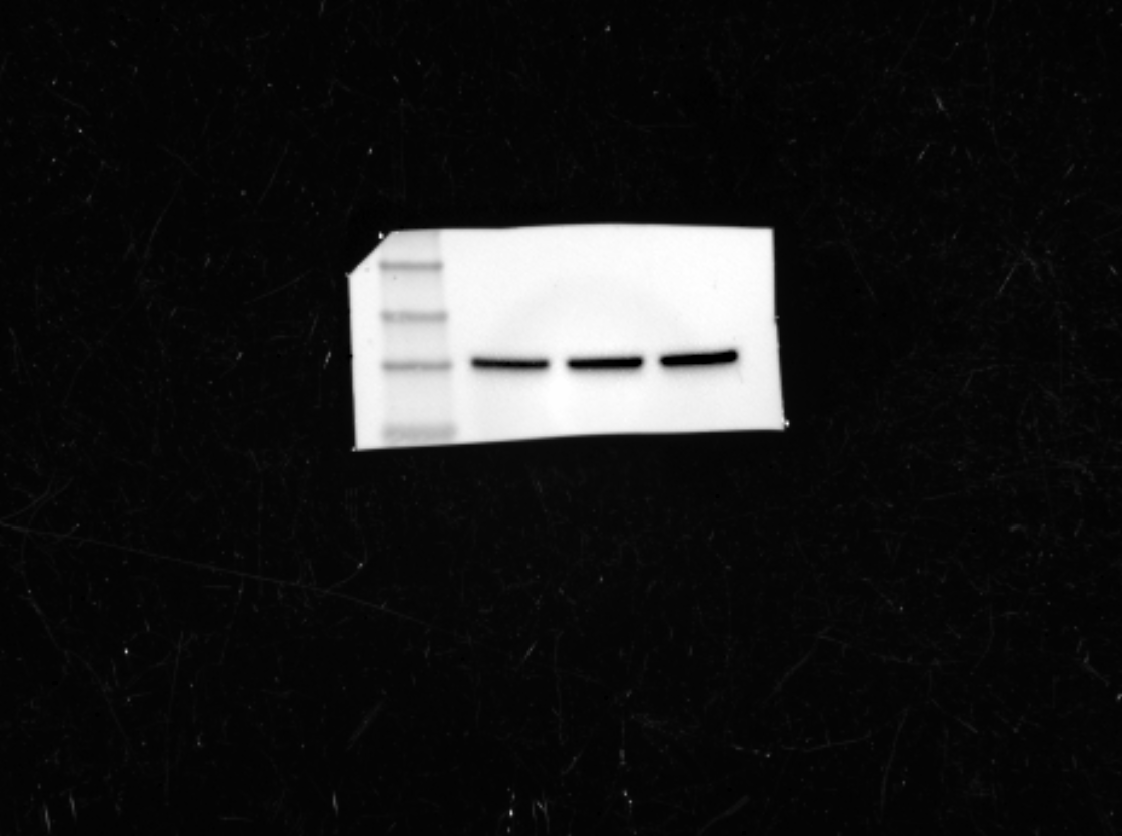

Supplement: Figure 1—figure supplement 1—source data 1. [file elife-85595-fig1-figsupp1-data1.zip › Figure 1-figure supplement 1-source data 1/Original_files/Figure 1-figure supplement 1_GAPDH_input.tif]

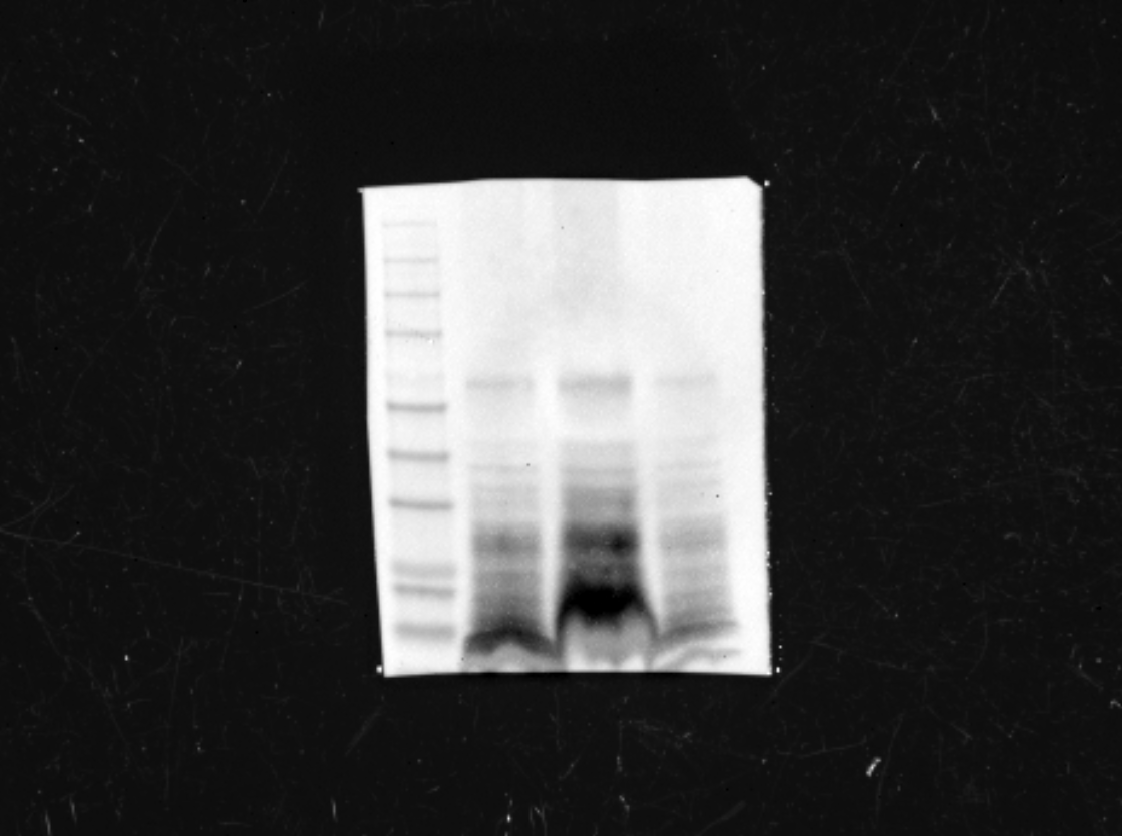

Supplement: Figure 1—figure supplement 1—source data 1. [file elife-85595-fig1-figsupp1-data1.zip › Figure 1-figure supplement 1-source data 1/Original_files/Figure 1-figure supplement 1_O-GlcNAc_input.tif]

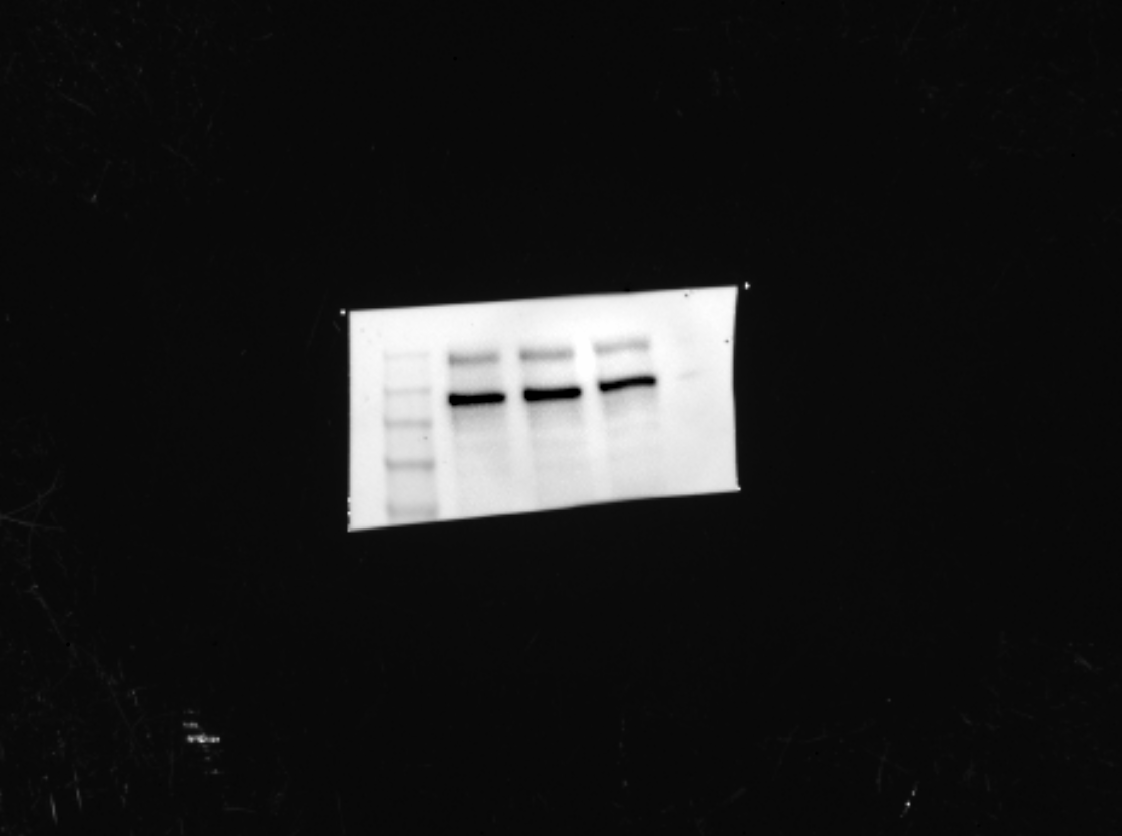

Supplement: Figure 1—figure supplement 1—source data 1. [file elife-85595-fig1-figsupp1-data1.zip › Figure 1-figure supplement 1-source data 1/Original_files/Figure 1-figure supplement 1_DNMT1_input.tif]

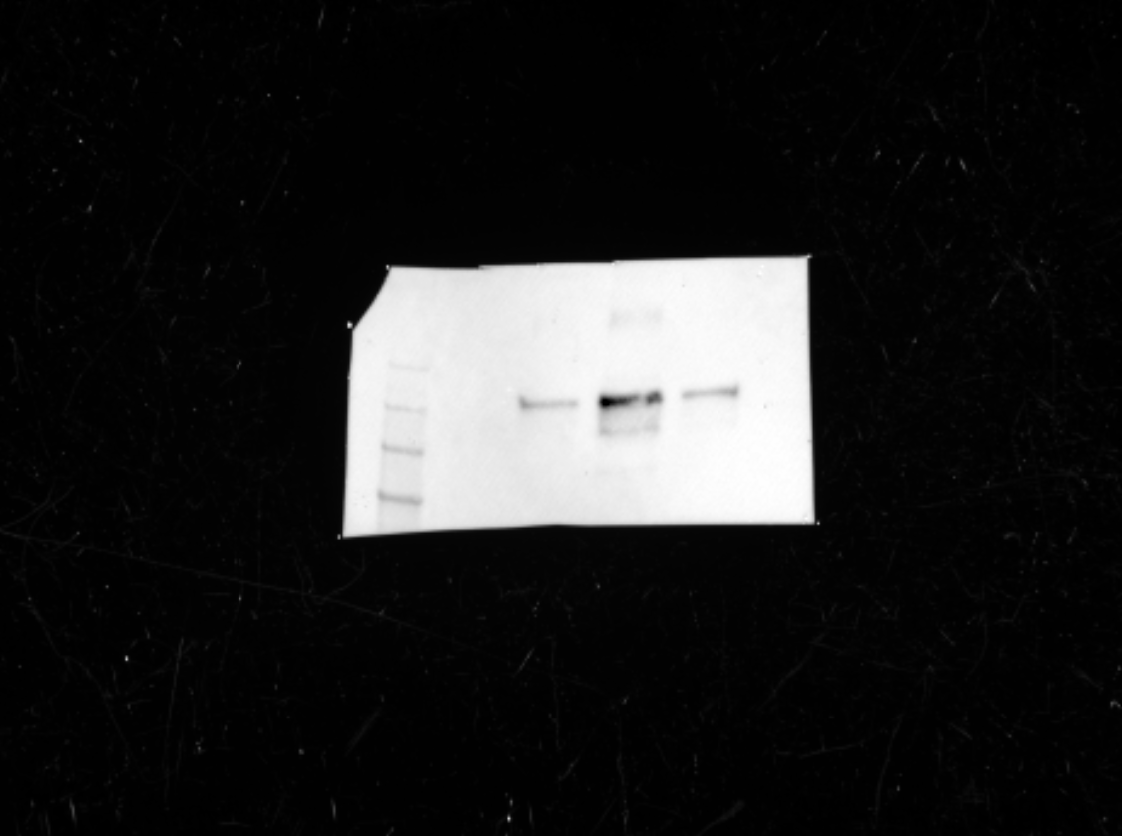

Supplement: Figure 1—figure supplement 1—source data 1. [file elife-85595-fig1-figsupp1-data1.zip › Figure 1-figure supplement 1-source data 1/Original_files/Figure 1-figure supplement 1_O-GlcNAc_IP.tif]

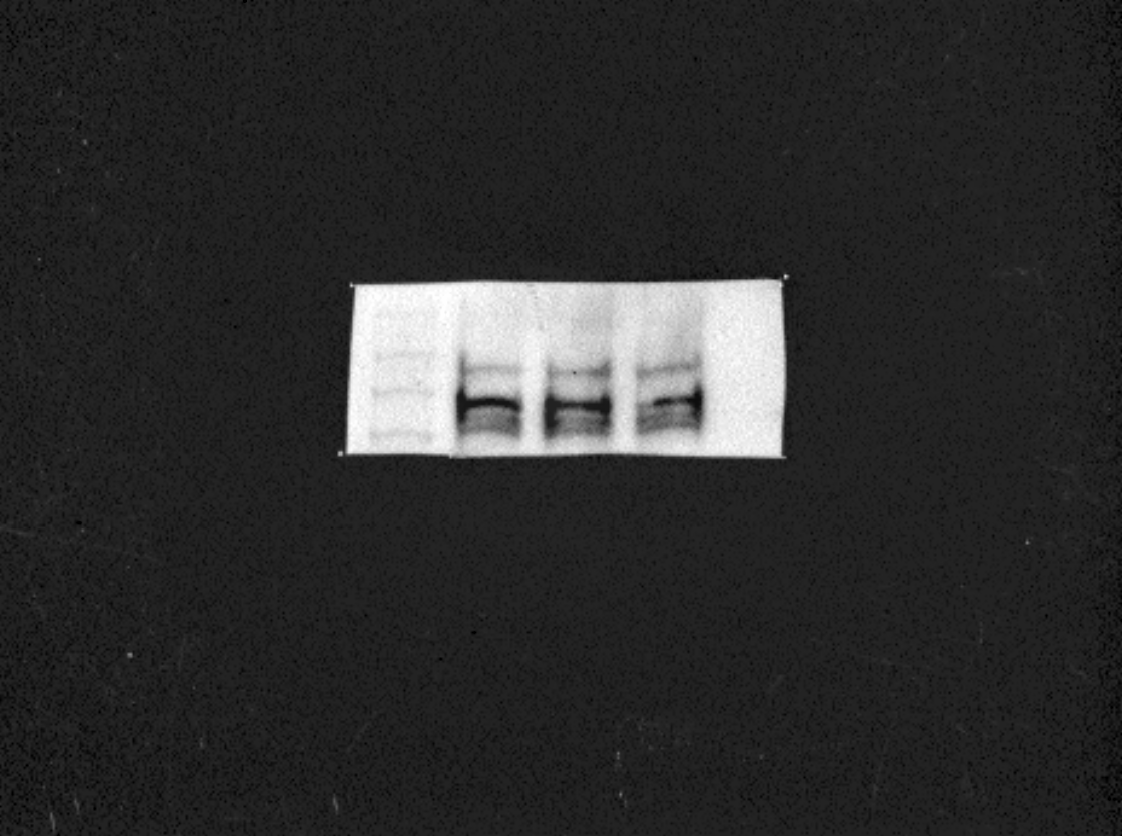

Supplement: Figure 1—figure supplement 1—source data 1. [file elife-85595-fig1-figsupp1-data1.zip › Figure 1-figure supplement 1-source data 1/Original_files/Figure 1-figure supplement 1_DNMT1_IP.tif]

Figure 1—figure supplement 2B

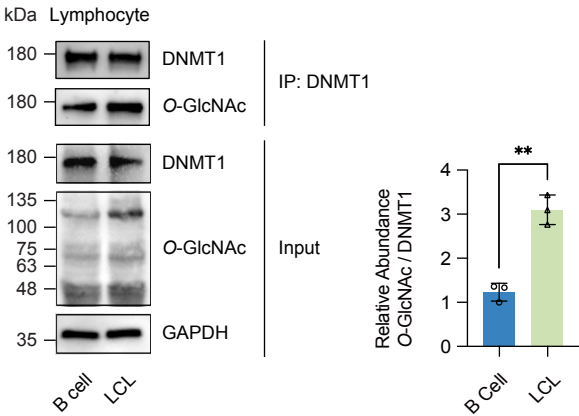

IP: DNMT1

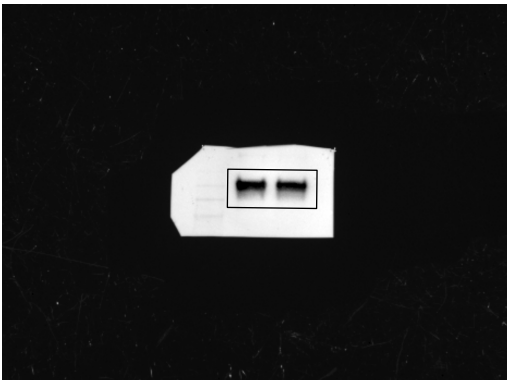

IP: O-GlcNAc

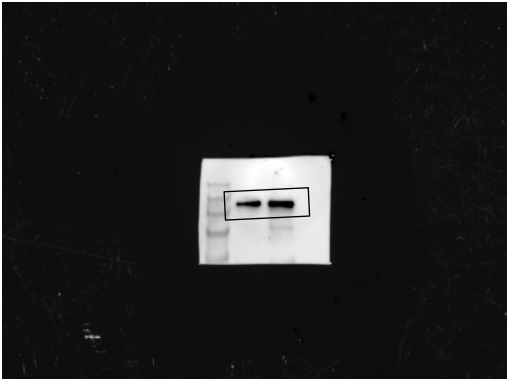

Input: DNMT1

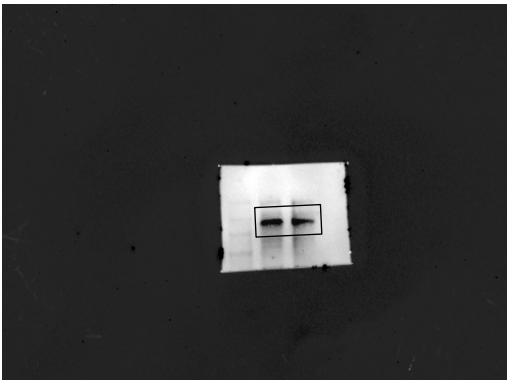

Input: O-GlcNAc

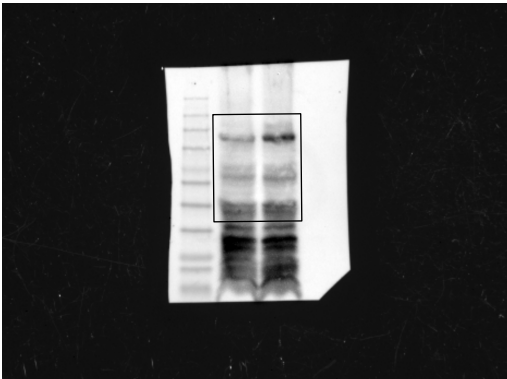

Input: GAPDH

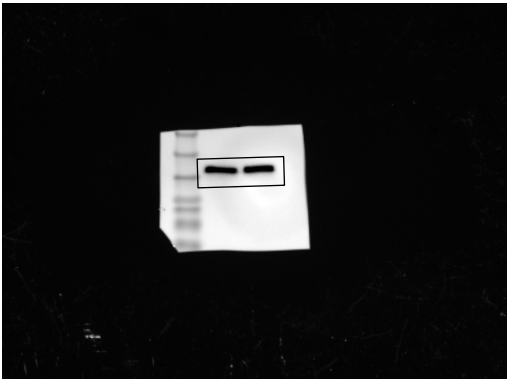

Supplement: Figure 1—figure supplement 2—source data 1. [file elife-85595-fig1-figsupp2-data1.zip › Figure 1-figure supplement 2-source data 1/Labeled_file/Figure 1-figure supplement 2B-source data 1.pdf]

Figure 1—figure supplement 2A

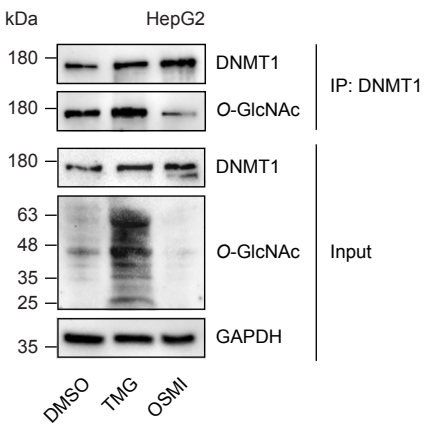

IP: DNMT1

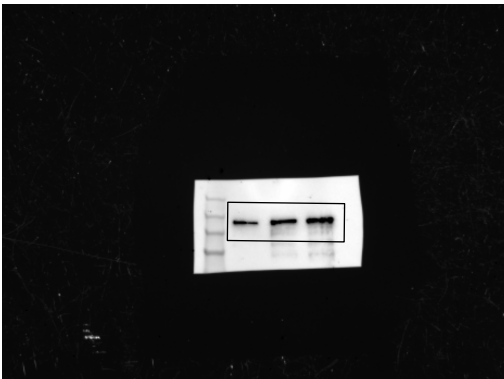

IP: O-GlcNAc

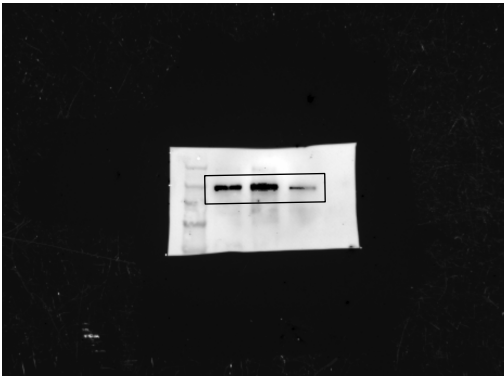

Input: DNMT1

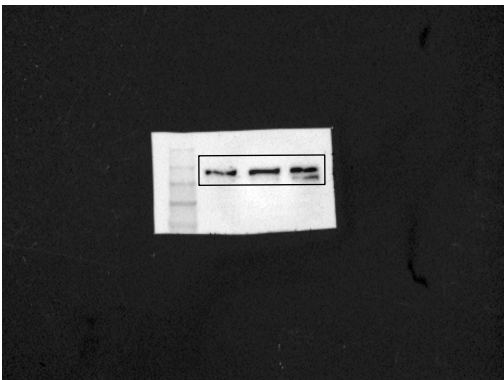

Input: O-GlcNAc

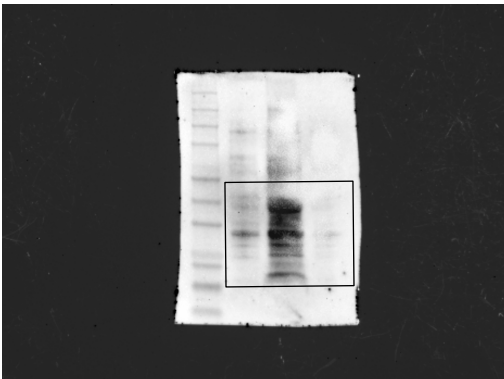

Input: GAPDH

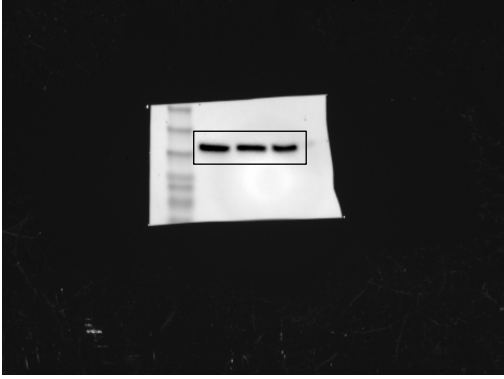

Supplement: Figure 1—figure supplement 2—source data 1. [file elife-85595-fig1-figsupp2-data1.zip › Figure 1-figure supplement 2-source data 1/Labeled_file/Figure 1-figure supplement 2A-source data 1.pdf]

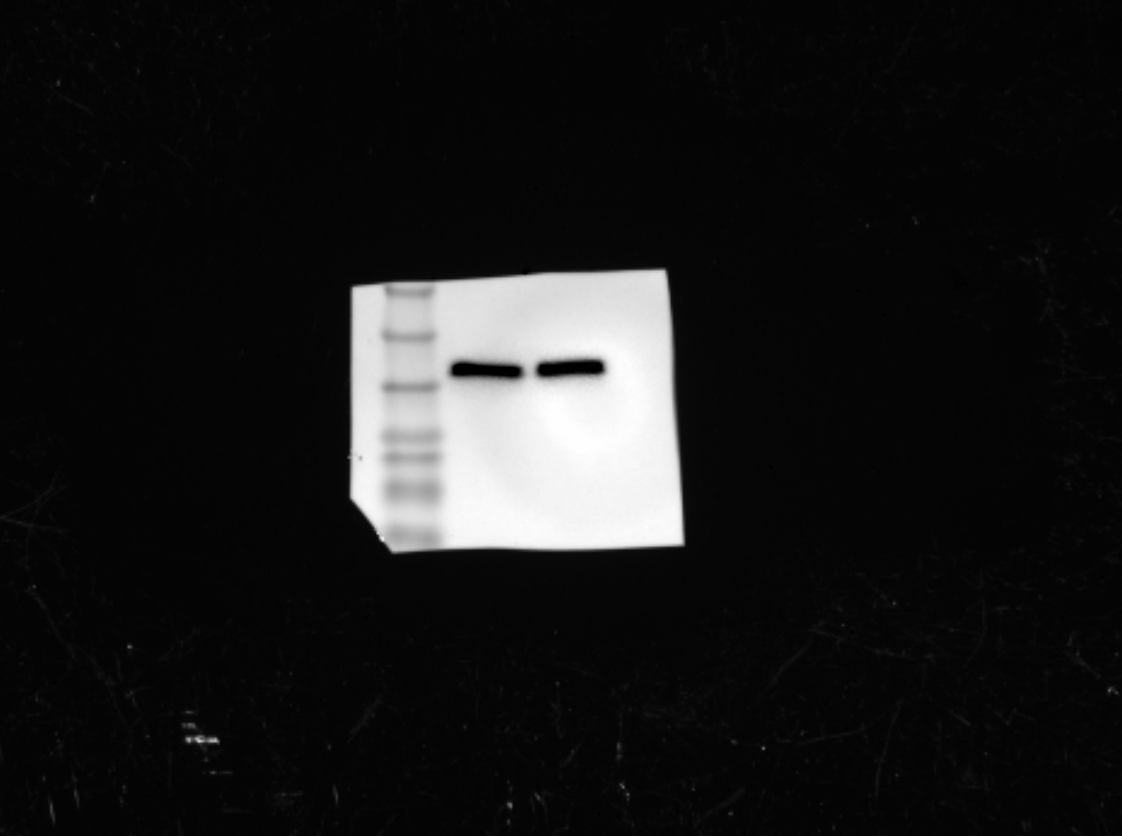

Supplement: Figure 1—figure supplement 2—source data 1. [file elife-85595-fig1-figsupp2-data1.zip › Figure 1-figure supplement 2-source data 1/Original_files/Figure 1-figure supplement 2B_GAPDH_input.tif]

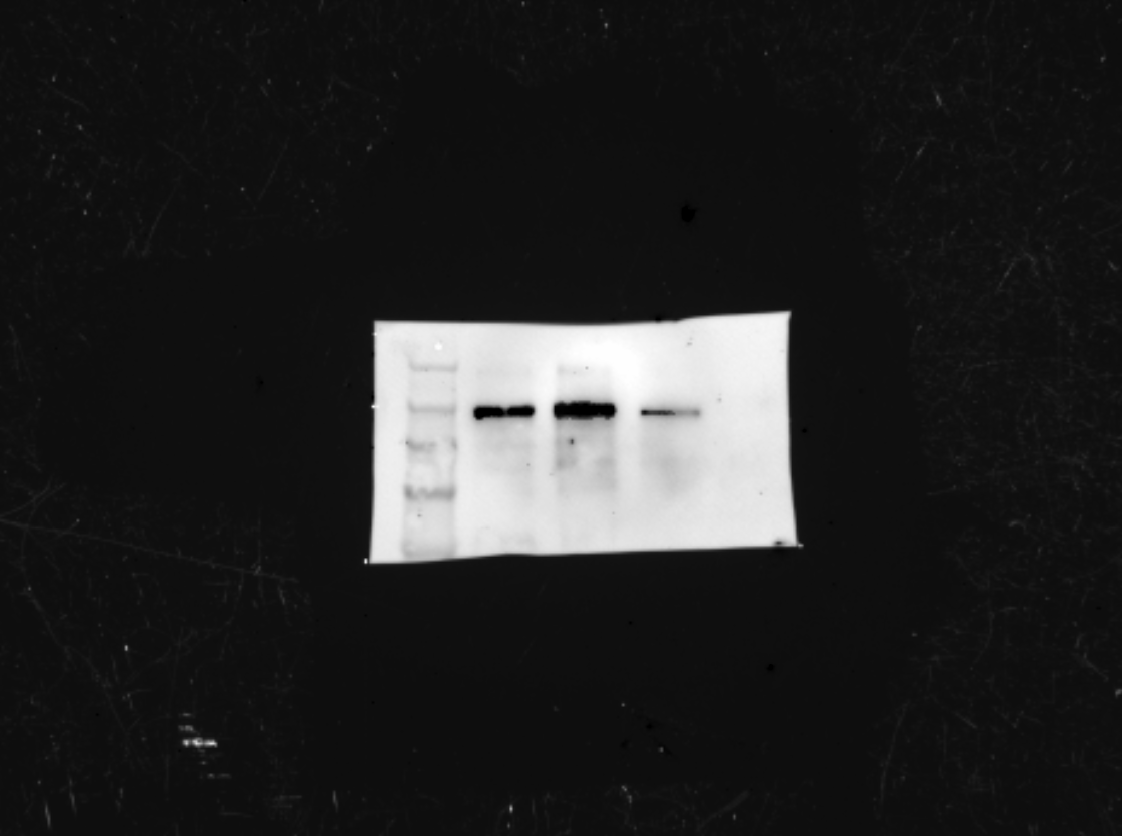

Supplement: Figure 1—figure supplement 2—source data 1. [file elife-85595-fig1-figsupp2-data1.zip › Figure 1-figure supplement 2-source data 1/Original_files/Figure 1-figure supplement 2A_O-GlcNAc_IP.tif]

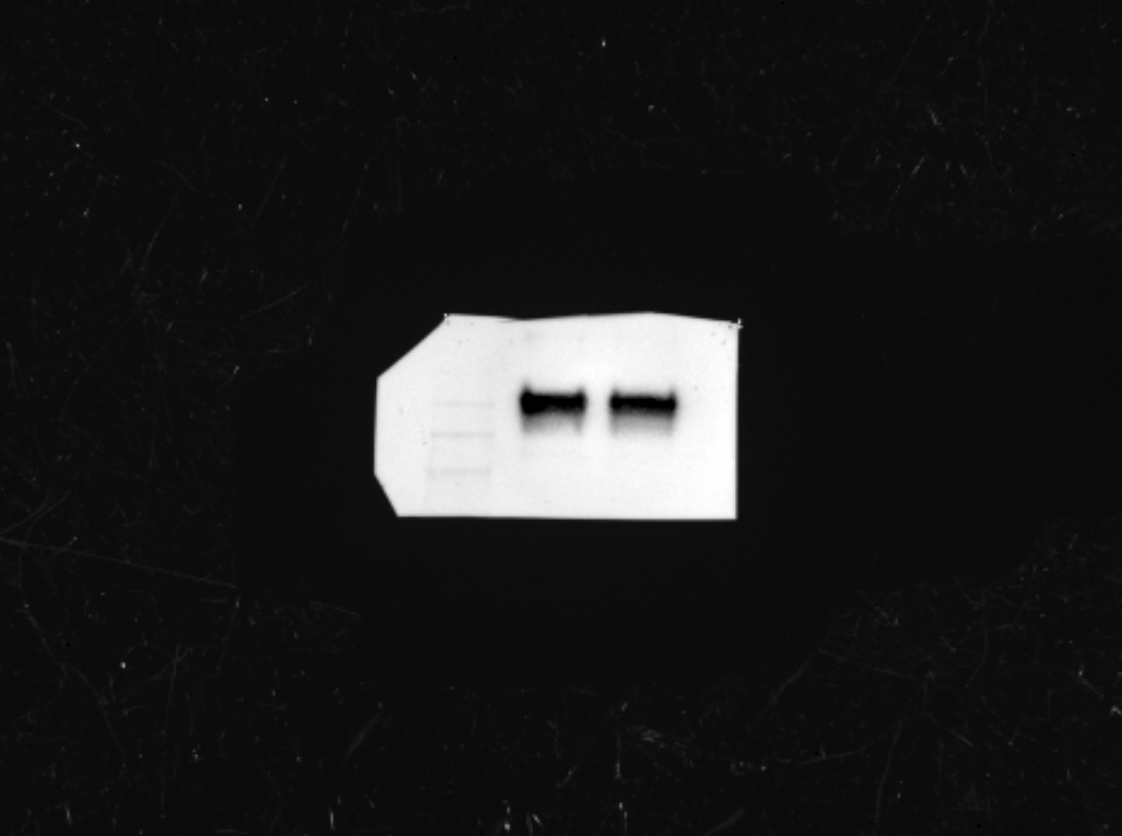

Supplement: Figure 1—figure supplement 2—source data 1. [file elife-85595-fig1-figsupp2-data1.zip › Figure 1-figure supplement 2-source data 1/Original_files/Figure 1-figure supplement 2B_DNMT1_IP.tif]

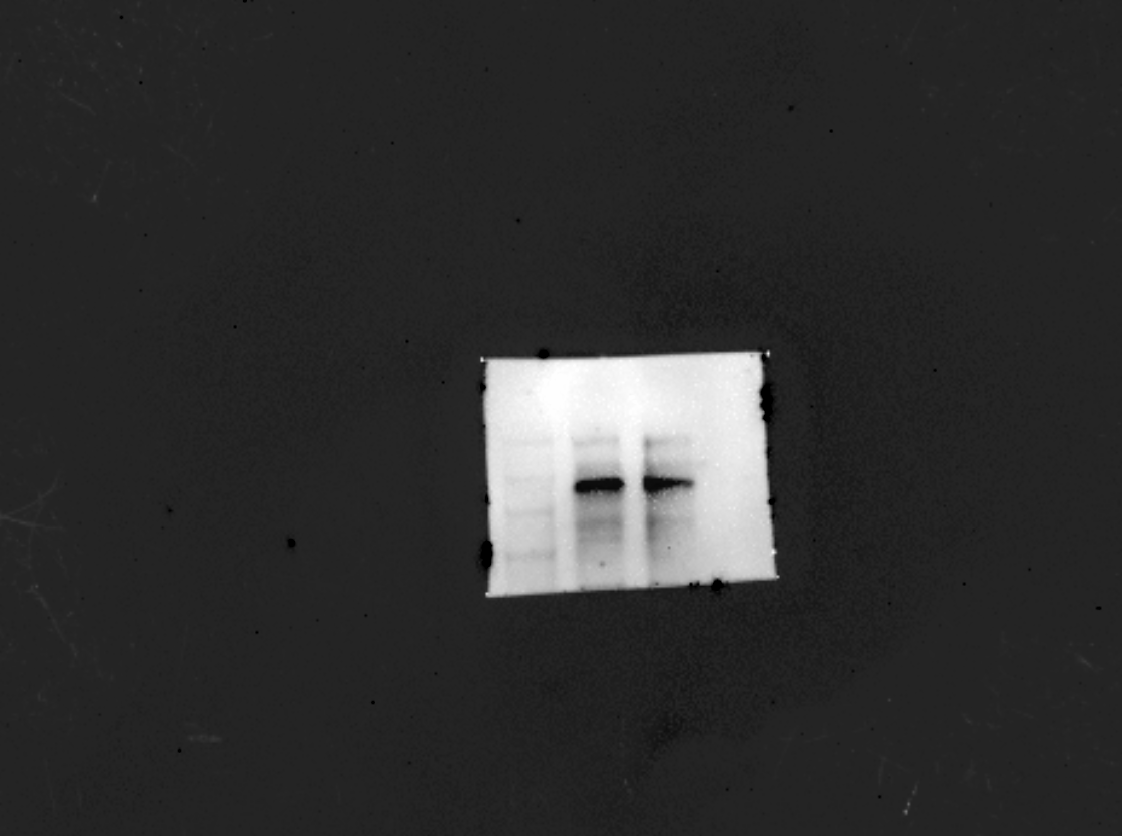

Supplement: Figure 1—figure supplement 2—source data 1. [file elife-85595-fig1-figsupp2-data1.zip › Figure 1-figure supplement 2-source data 1/Original_files/Figure 1-figure supplement 2B_DNMT1_input.tif]

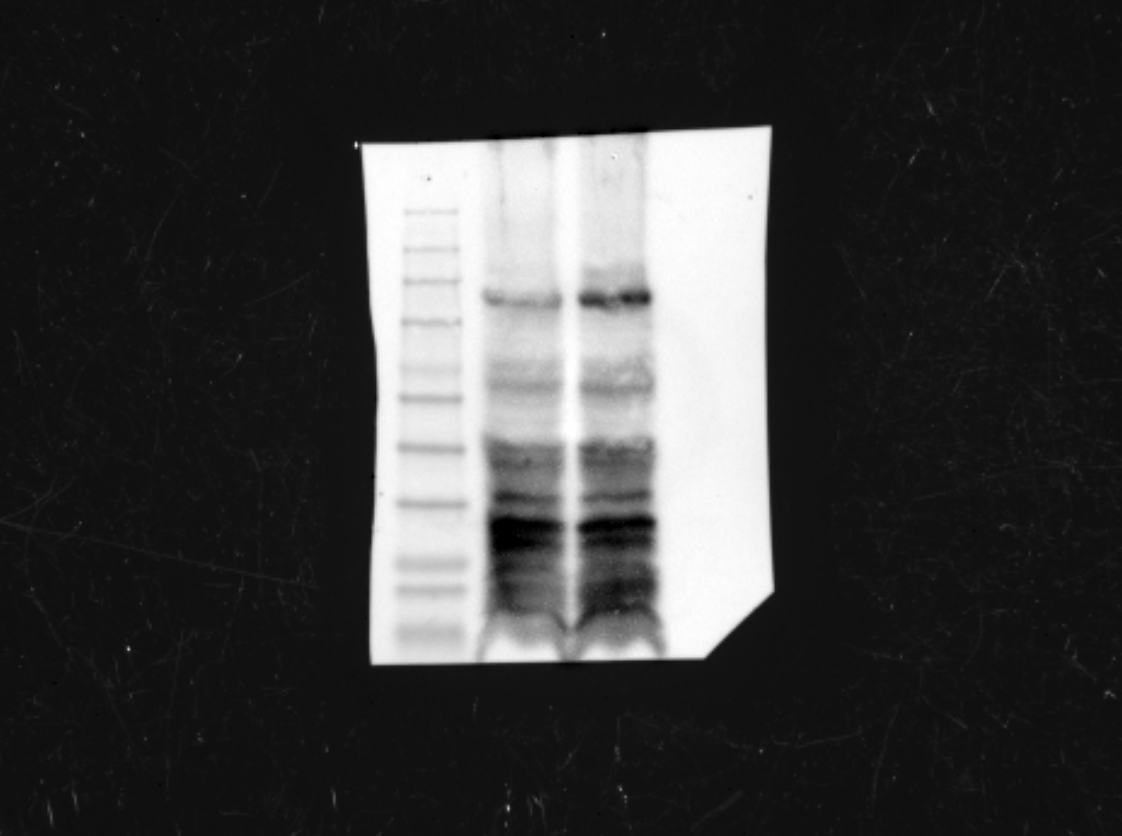

Supplement: Figure 1—figure supplement 2—source data 1. [file elife-85595-fig1-figsupp2-data1.zip › Figure 1-figure supplement 2-source data 1/Original_files/Figure 1-figure supplement 2B_O-GlcNAc_input.tif]

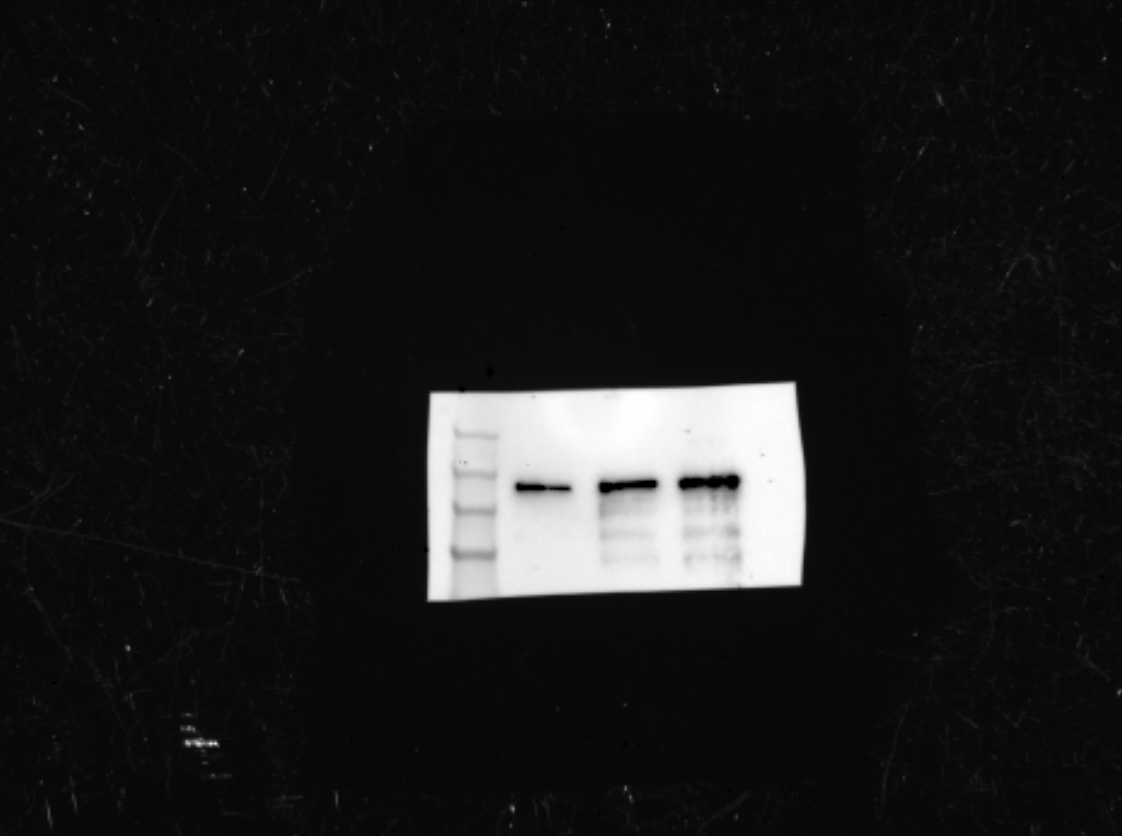

Supplement: Figure 1—figure supplement 2—source data 1. [file elife-85595-fig1-figsupp2-data1.zip › Figure 1-figure supplement 2-source data 1/Original_files/Figure 1-figure supplement 2A_DNMT1_IP.tif]

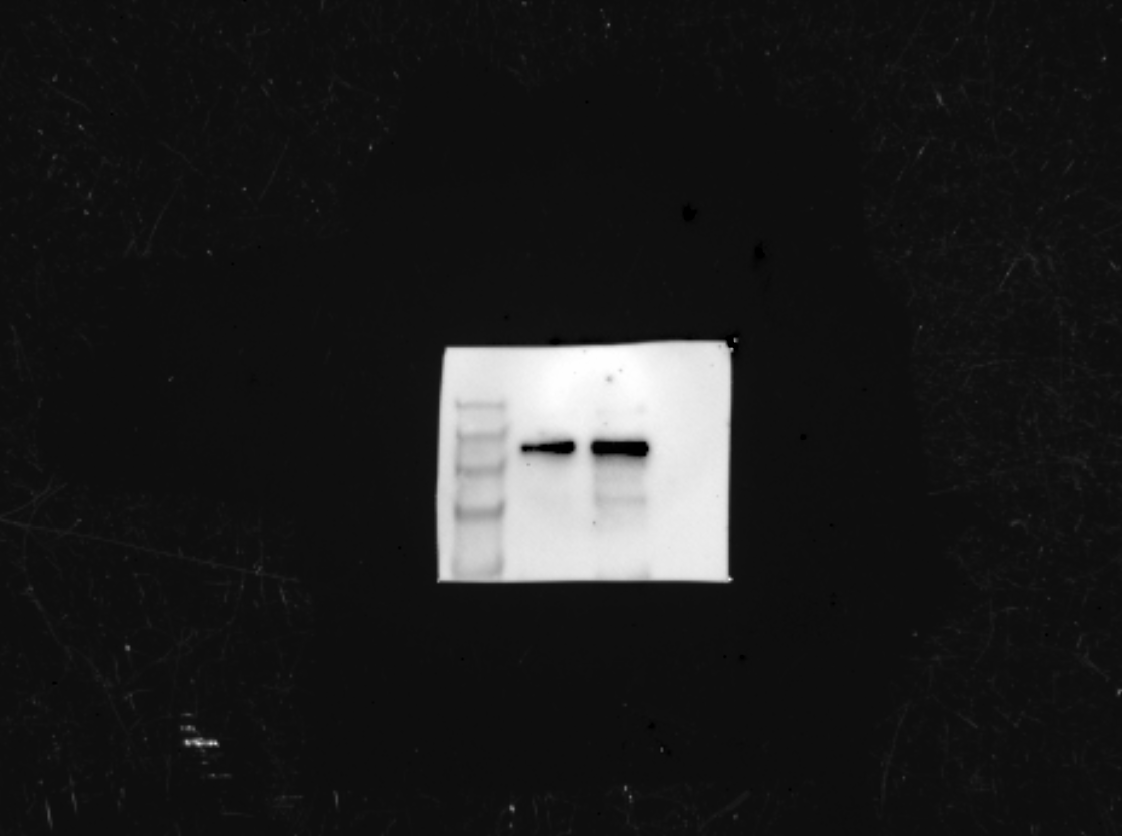

Supplement: Figure 1—figure supplement 2—source data 1. [file elife-85595-fig1-figsupp2-data1.zip › Figure 1-figure supplement 2-source data 1/Original_files/Figure 1-figure supplement 2B_O-GlcNAc_IP.tif]

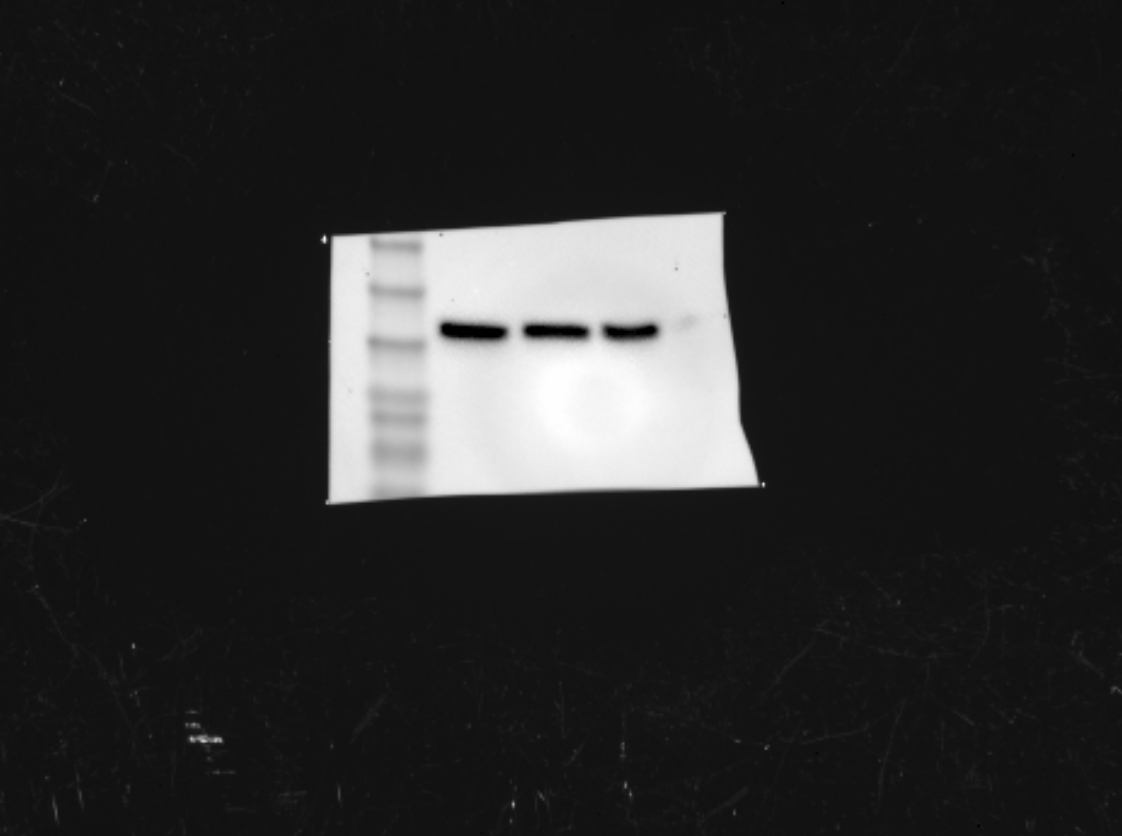

Supplement: Figure 1—figure supplement 2—source data 1. [file elife-85595-fig1-figsupp2-data1.zip › Figure 1-figure supplement 2-source data 1/Original_files/Figure 1-figure supplement 2A_GAPDH_input.tif]

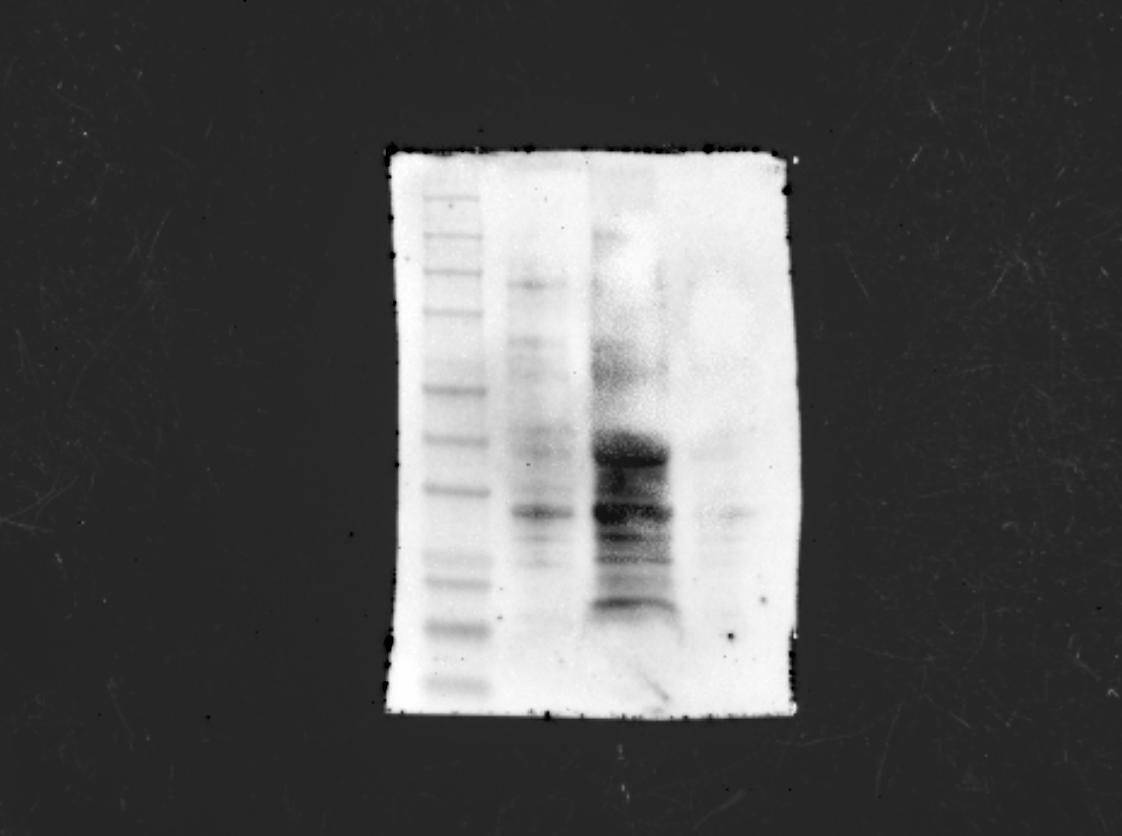

Supplement: Figure 1—figure supplement 2—source data 1. [file elife-85595-fig1-figsupp2-data1.zip › Figure 1-figure supplement 2-source data 1/Original_files/Figure 1-figure supplement 2A_O-GlcNAc_input.tif]

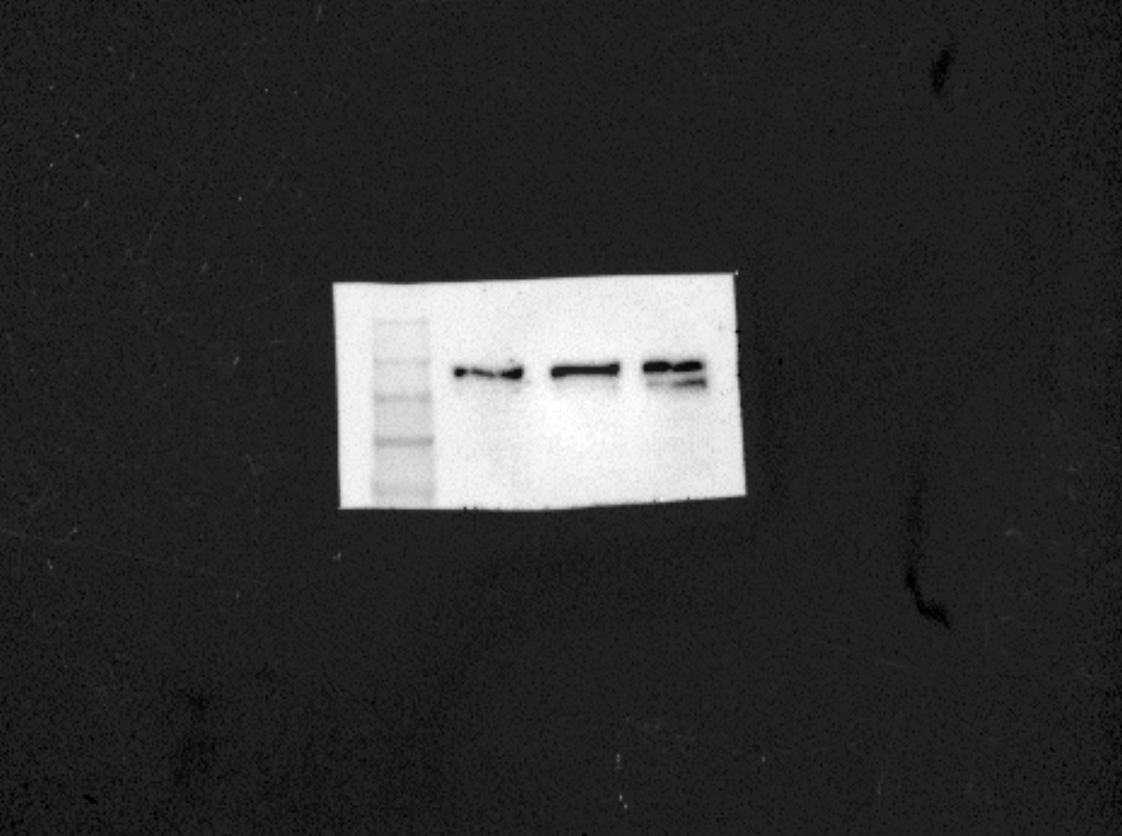

Supplement: Figure 1—figure supplement 2—source data 1. [file elife-85595-fig1-figsupp2-data1.zip › Figure 1-figure supplement 2-source data 1/Original_files/Figure 1-figure supplement 2A_DNMT1_input.tif]

Figure 1—figure supplement 3A

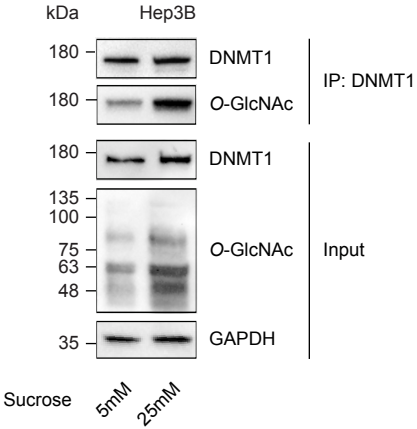

IP: DNMT1

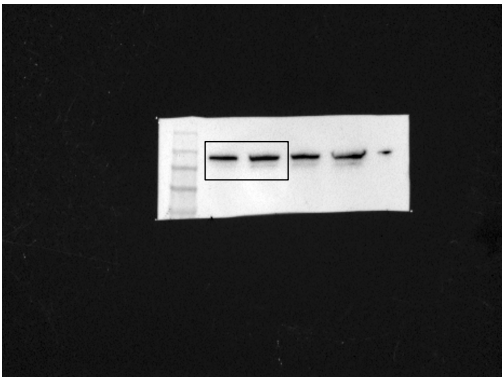

IP: O-GlcNAc

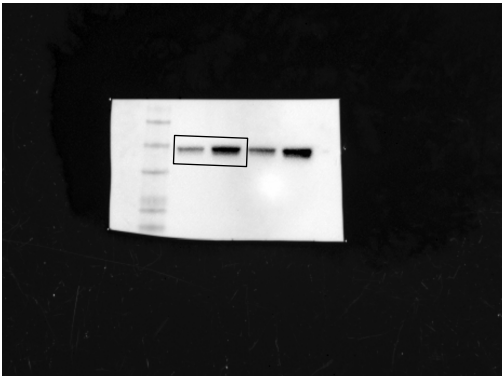

Input: DNMT1

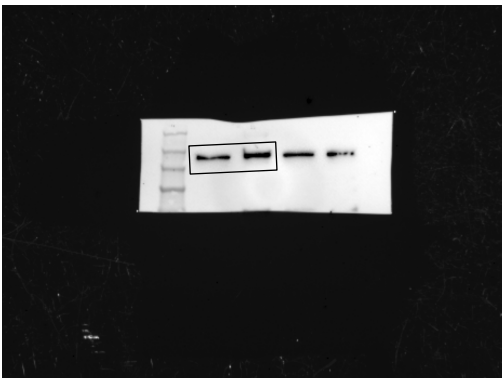

Input: O-GlcNAc

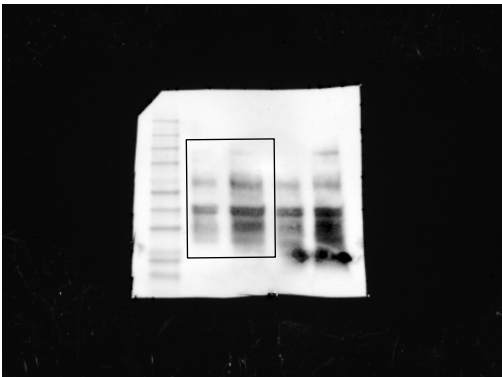

Input: GAPDH

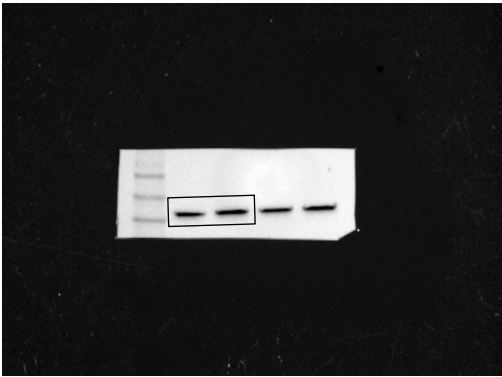

Supplement: Figure 1—figure supplement 3—source data 1. [file elife-85595-fig1-figsupp3-data1.zip › Figure 1-figure supplement 3-source data 1/Labeled_file/Figure 1-figure supplement 3A-source data 1.pdf]

Figure 1—figure supplement 3B

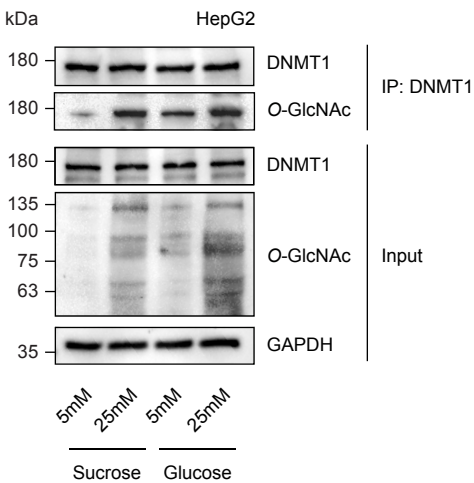

IP: DNMT1

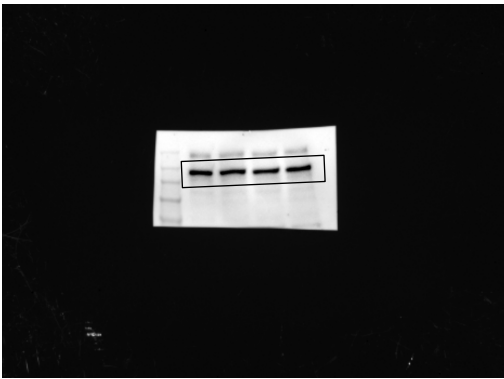

IP: O-GlcNAc

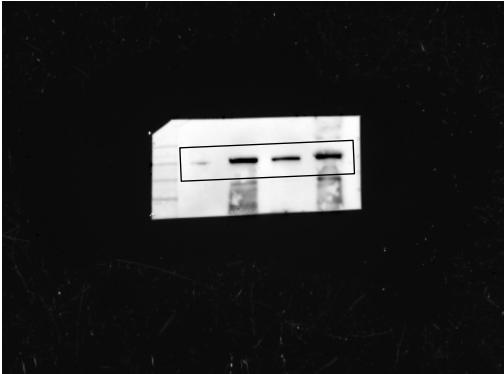

Input: DNMT1

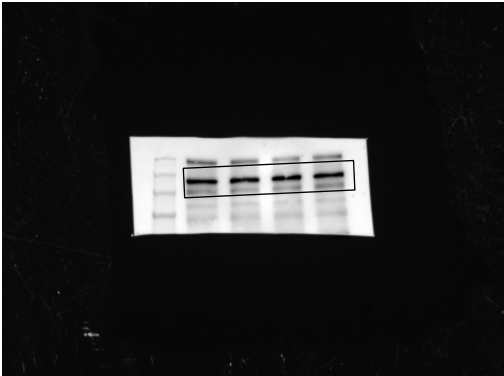

Input: O-GlcNAc

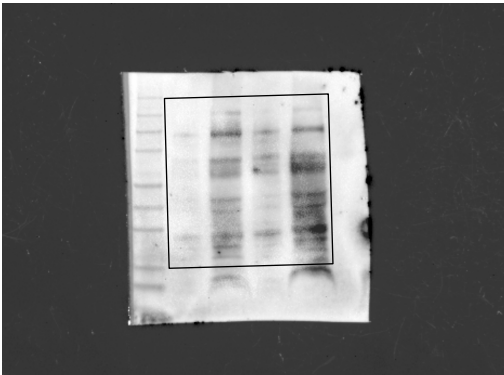

Input: GAPDH

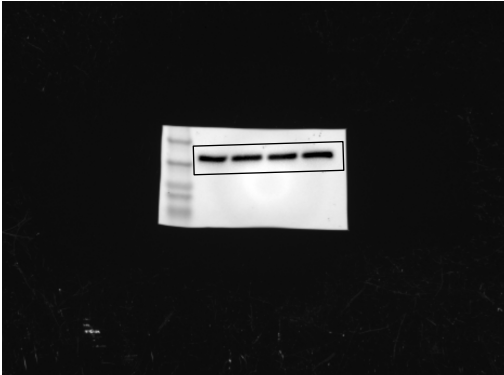

Supplement: Figure 1—figure supplement 3—source data 1. [file elife-85595-fig1-figsupp3-data1.zip › Figure 1-figure supplement 3-source data 1/Labeled_file/Figure 1-figure supplement 3B-source data 1.pdf]

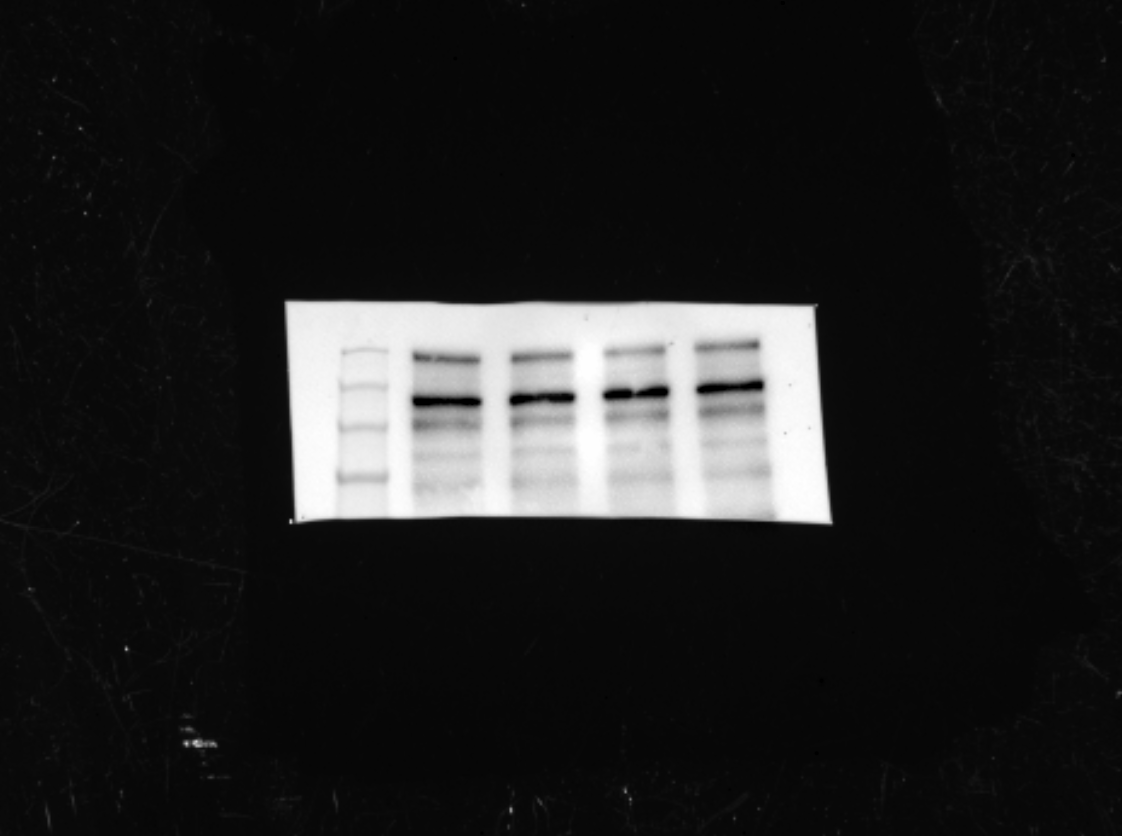

Supplement: Figure 1—figure supplement 3—source data 1. [file elife-85595-fig1-figsupp3-data1.zip › Figure 1-figure supplement 3-source data 1/Original_files/Figure 1-figure supplement 3B_DNMT1_input.tif]

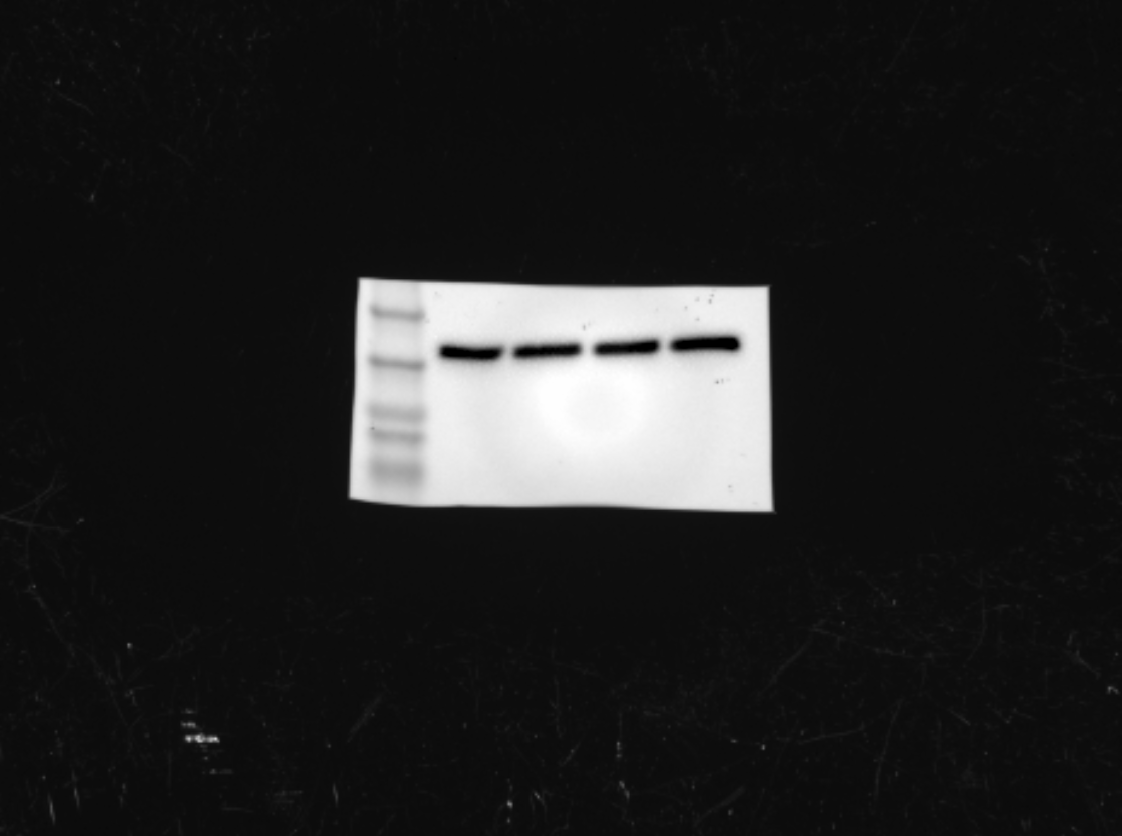

Supplement: Figure 1—figure supplement 3—source data 1. [file elife-85595-fig1-figsupp3-data1.zip › Figure 1-figure supplement 3-source data 1/Original_files/Figure 1-figure supplement 3B_GAPDH_input.tif]

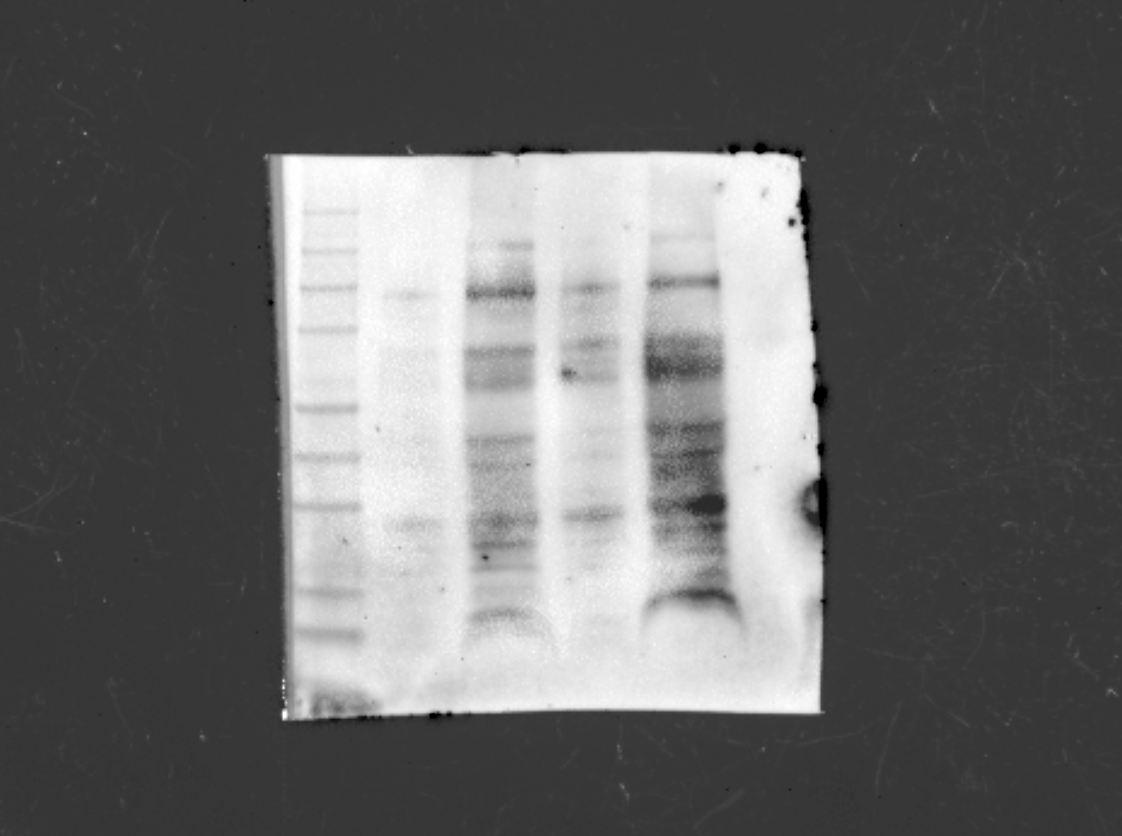

Supplement: Figure 1—figure supplement 3—source data 1. [file elife-85595-fig1-figsupp3-data1.zip › Figure 1-figure supplement 3-source data 1/Original_files/Figure 1-figure supplement 3B_O-GlcNAc_input.tif]

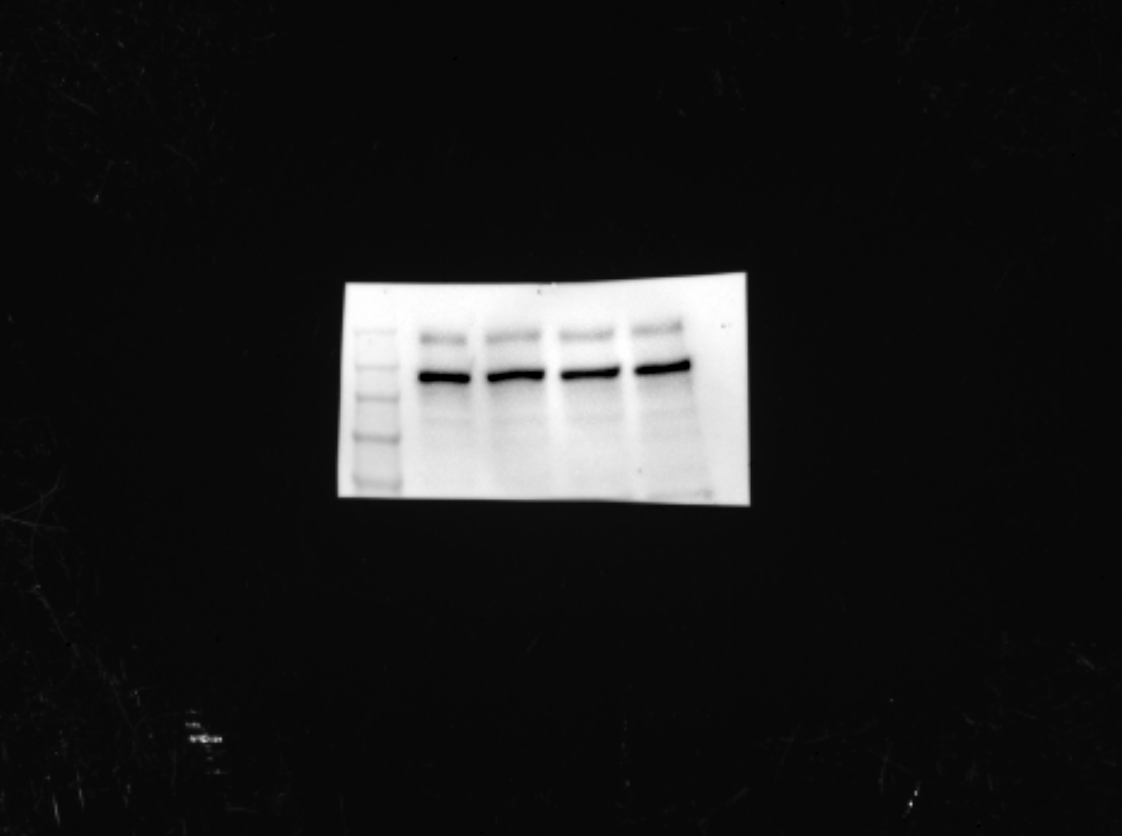

Supplement: Figure 1—figure supplement 3—source data 1. [file elife-85595-fig1-figsupp3-data1.zip › Figure 1-figure supplement 3-source data 1/Original_files/Figure 1-figure supplement 3B_DNMT1_IP.tif]

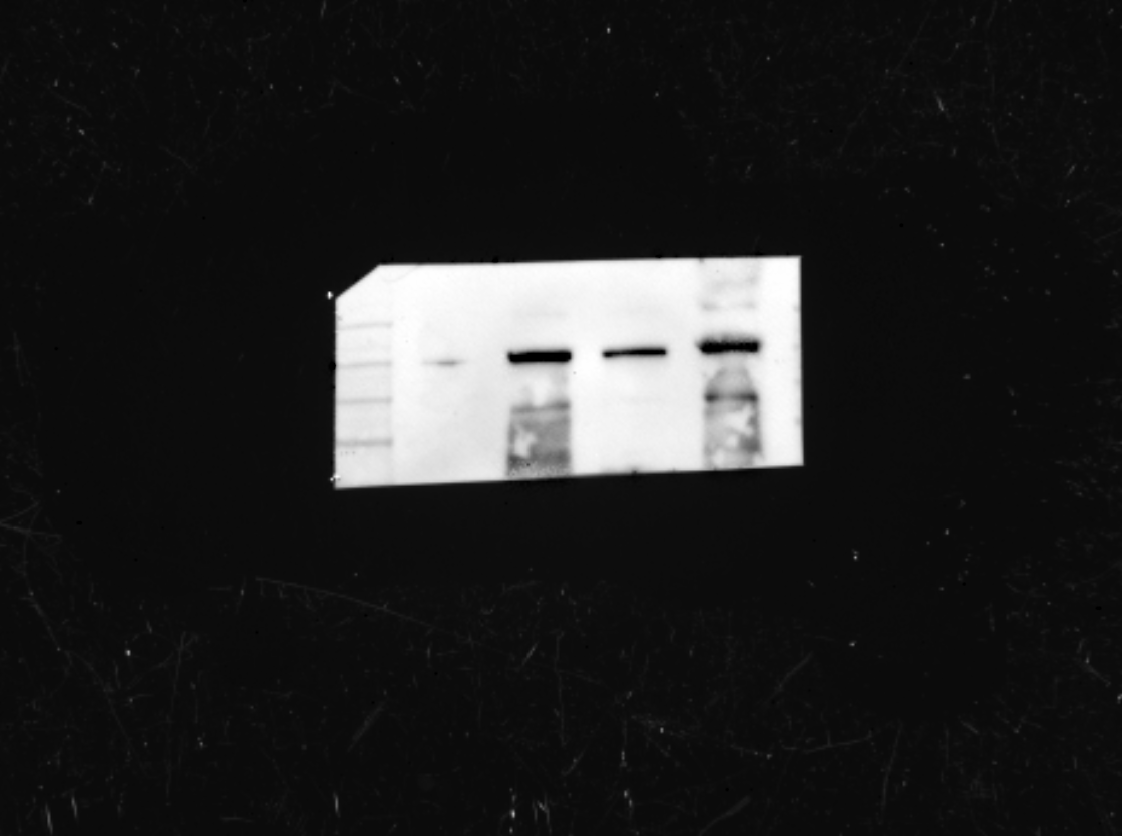

Supplement: Figure 1—figure supplement 3—source data 1. [file elife-85595-fig1-figsupp3-data1.zip › Figure 1-figure supplement 3-source data 1/Original_files/Figure 1-figure supplement 3B_O-GlcNAc_IP.tif]

Figure 1—figure supplement 5

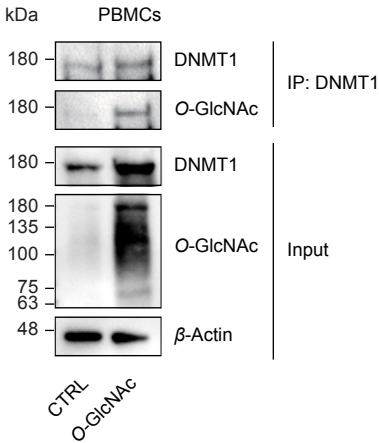

IP: DNMT1

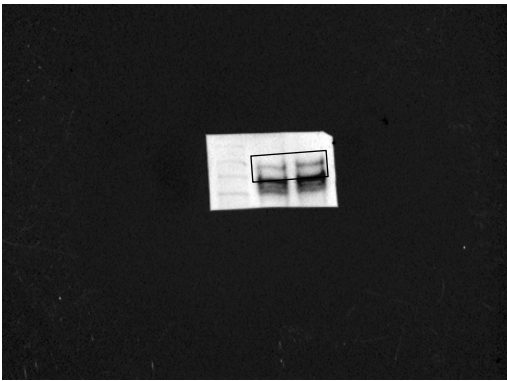

IP: O-GlcNAc

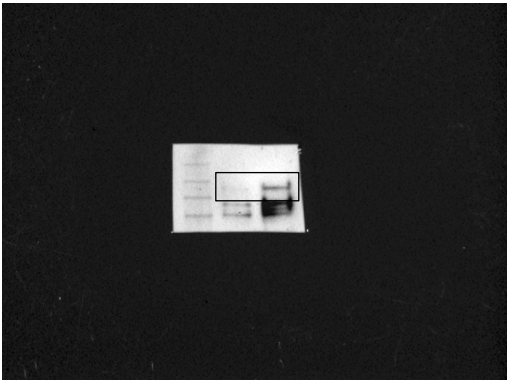

Input: DNMT1

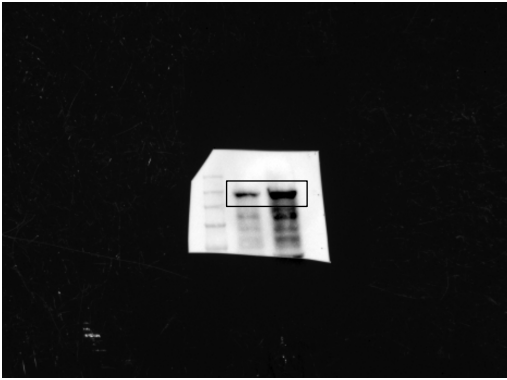

Input: O-GlcNAc

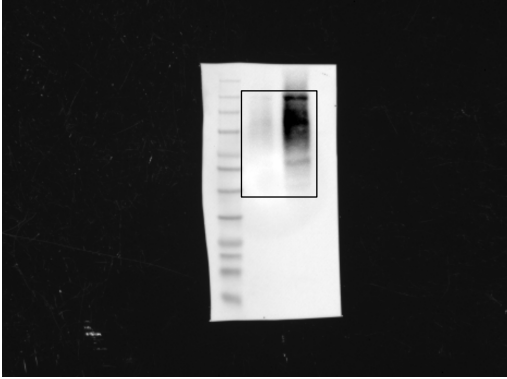

Input: GAPDH

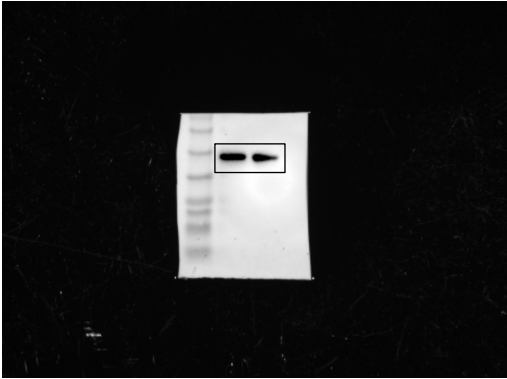

Supplement: Figure 1—figure supplement 5—source data 1. [file elife-85595-fig1-figsupp5-data1.zip › Figure 1-figure supplement 5-source data 1/Labeled_file/Figure 1-figure supplement 5-source data 1.pdf]

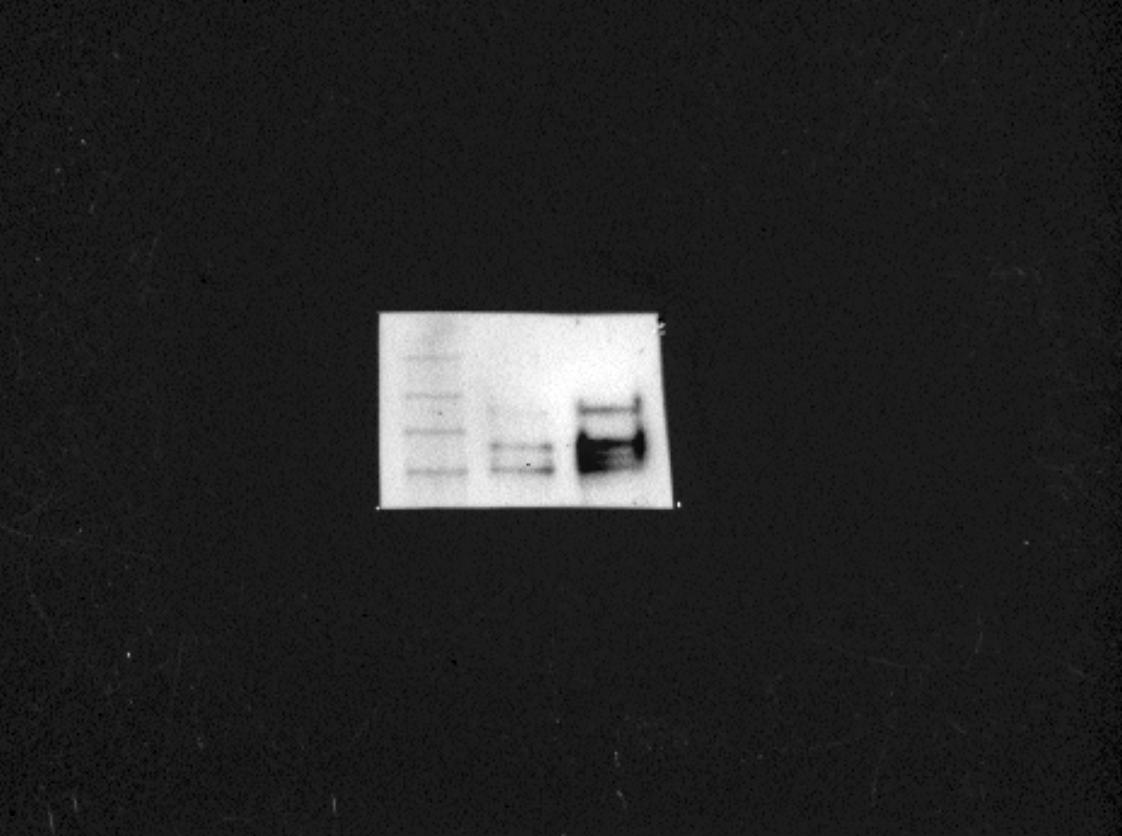

Supplement: Figure 1—figure supplement 5—source data 1. [file elife-85595-fig1-figsupp5-data1.zip › Figure 1-figure supplement 5-source data 1/Original_files/Figure 1-figure supplement 5_O-GlcNAc_IP.tif]

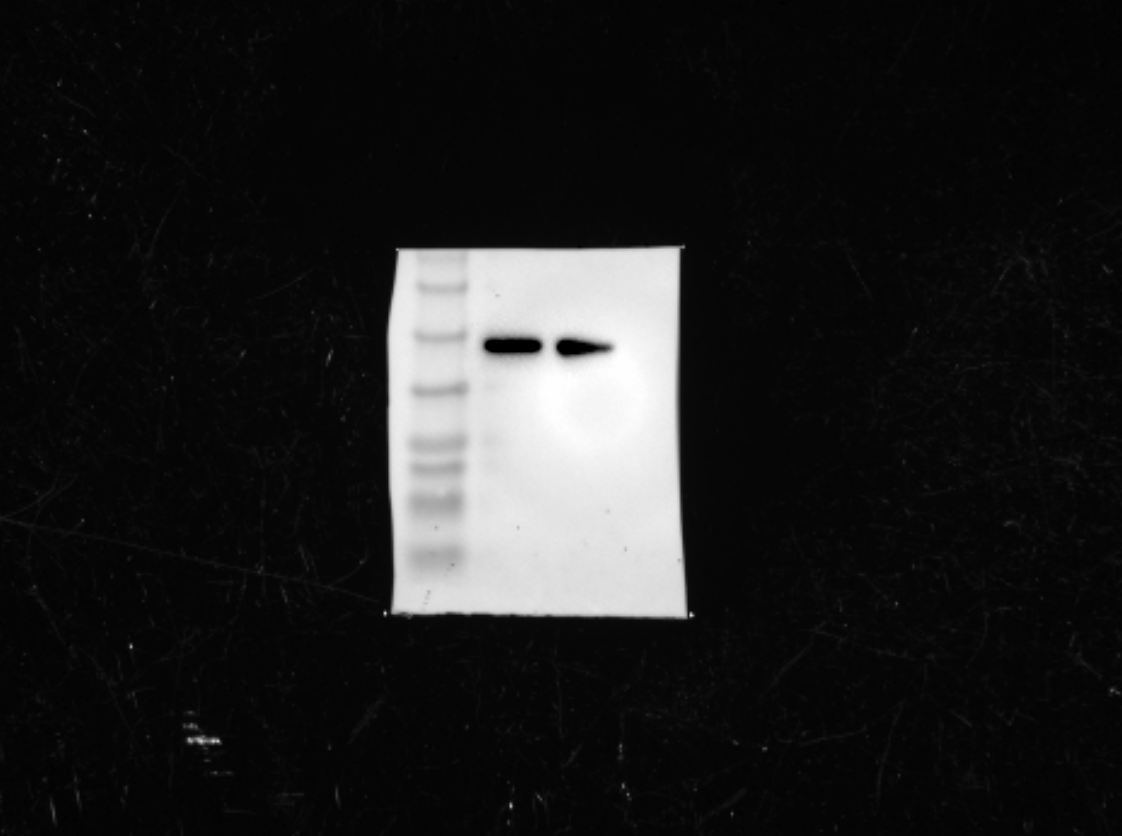

Supplement: Figure 1—figure supplement 5—source data 1. [file elife-85595-fig1-figsupp5-data1.zip › Figure 1-figure supplement 5-source data 1/Original_files/Figure 1-figure supplement 5_actin_input.tif]

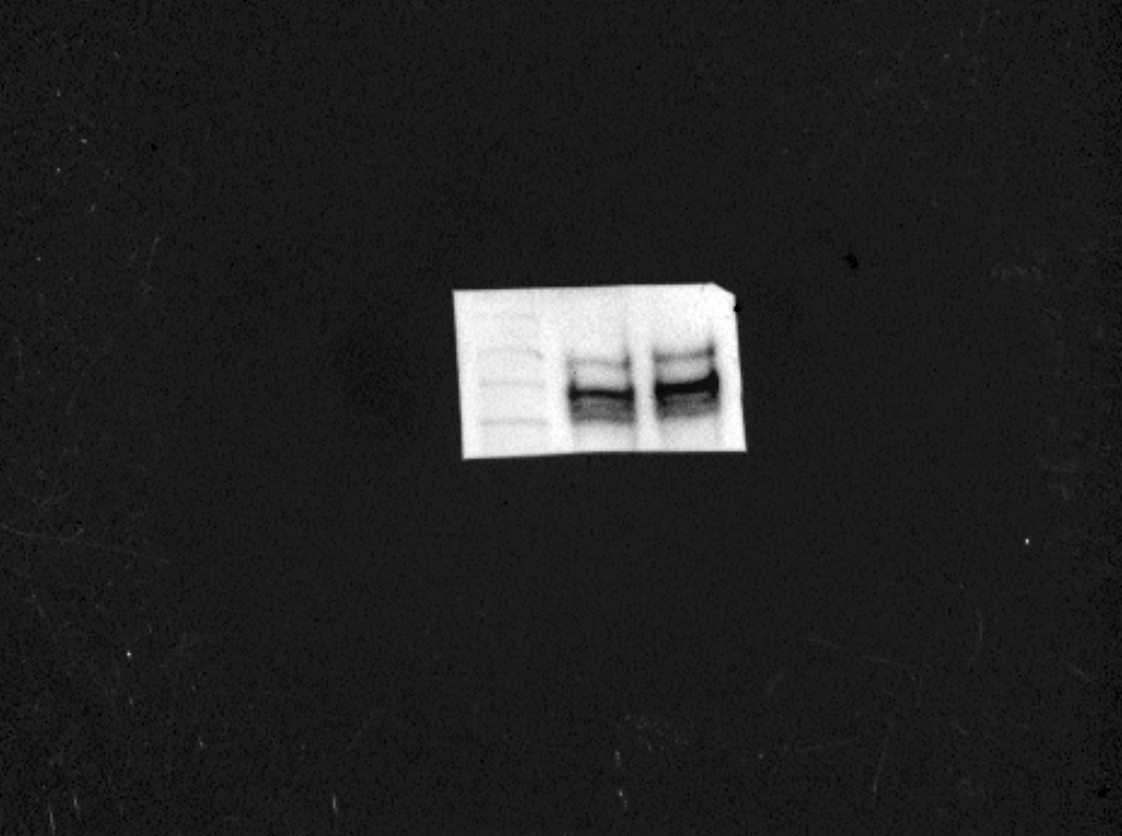

Supplement: Figure 1—figure supplement 5—source data 1. [file elife-85595-fig1-figsupp5-data1.zip › Figure 1-figure supplement 5-source data 1/Original_files/Figure 1-figure supplement 5_DNMT1_IP.tif]

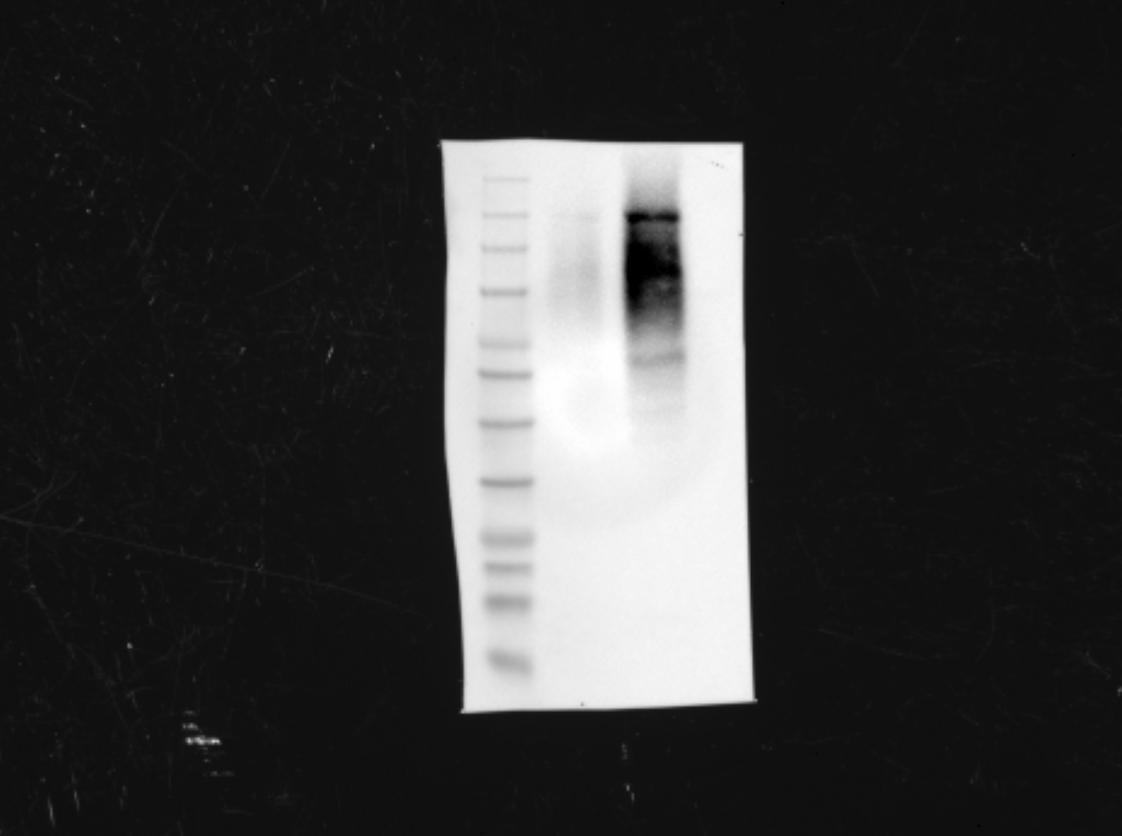

Supplement: Figure 1—figure supplement 5—source data 1. [file elife-85595-fig1-figsupp5-data1.zip › Figure 1-figure supplement 5-source data 1/Original_files/Figure 1-figure supplement 5_O-GlcNAc_input.tif]

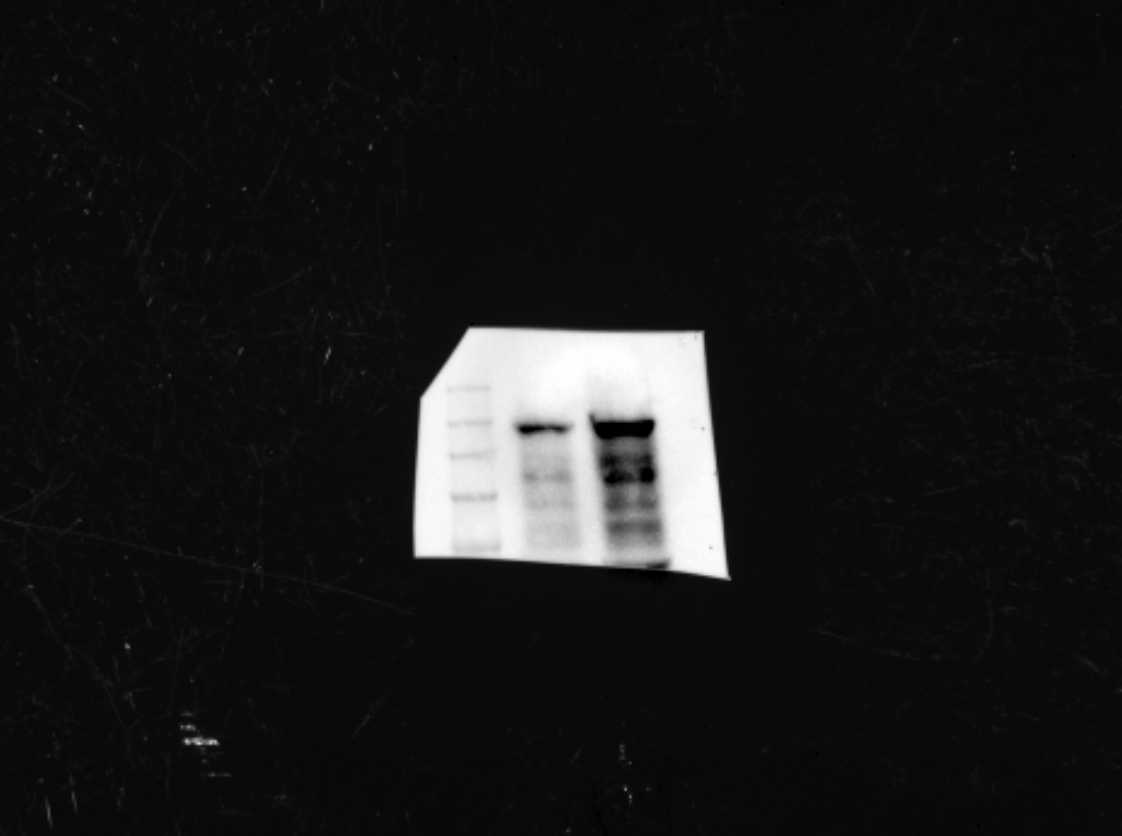

Supplement: Figure 1—figure supplement 5—source data 1. [file elife-85595-fig1-figsupp5-data1.zip › Figure 1-figure supplement 5-source data 1/Original_files/Figure 1-figure supplement 5_DNMT1_input.tif]

Figure 2D

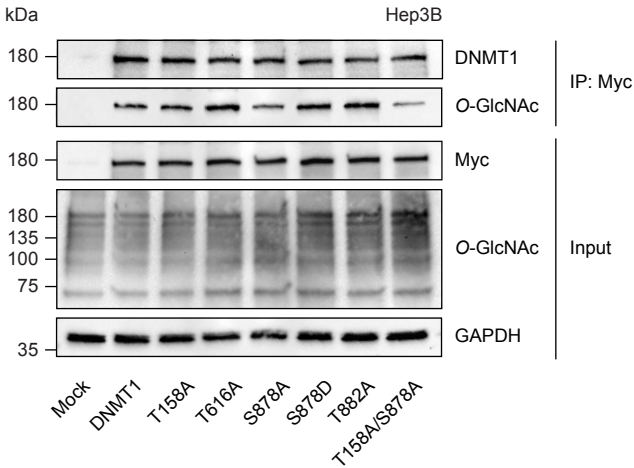

IP: DNMT1

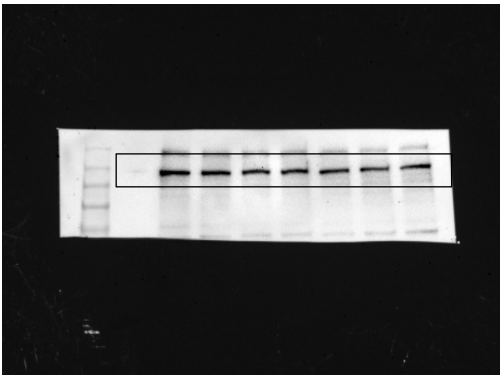

IP: O-GlcNAc

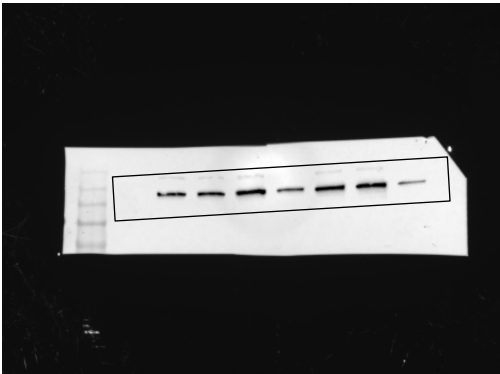

Input: Myc

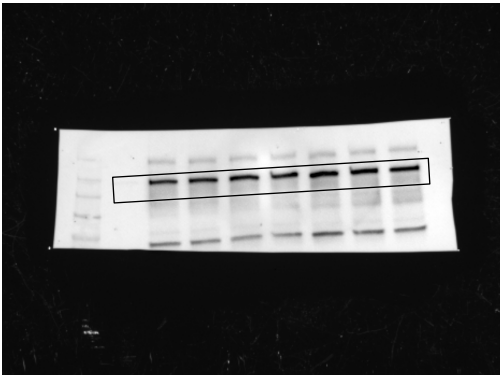

Input: O-GlcNAc

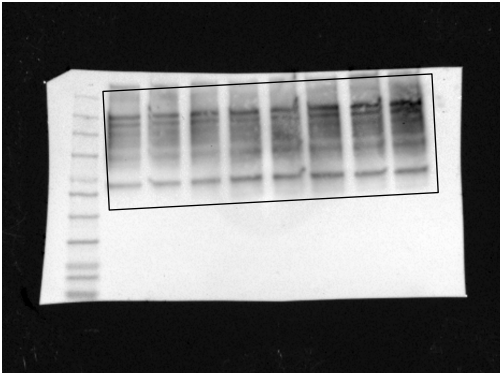

Input: GAPDH

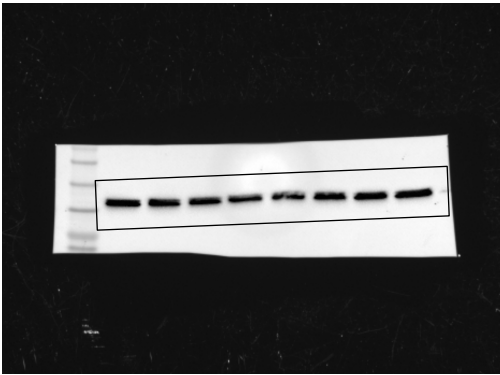

Supplement: Figure 2—source data 1. [file elife-85595-fig2-data1.zip › Figure 2-source data 1/Labeled_file/Figure 2D-source data 1.pdf]

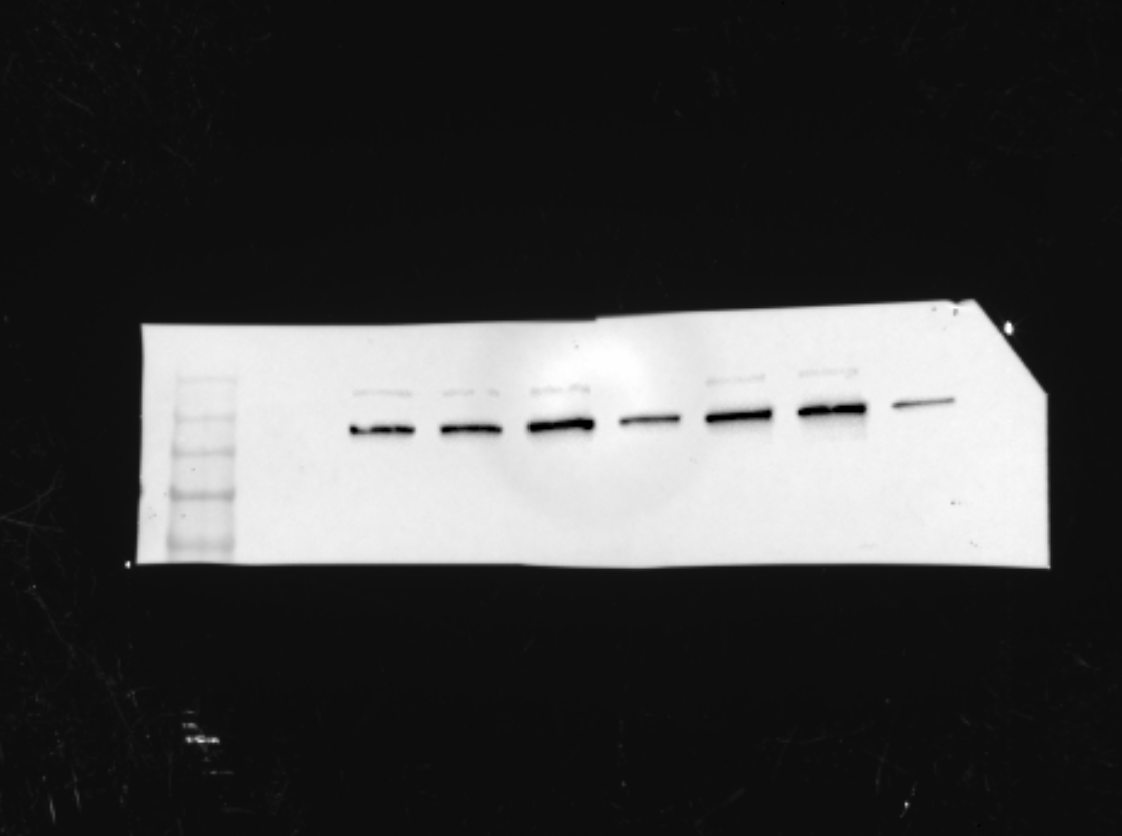

Supplement: Figure 2—source data 1. [file elife-85595-fig2-data1.zip › Figure 2-source data 1/Original_files/Figure 2D_O-GlcNAc_IP.tif]

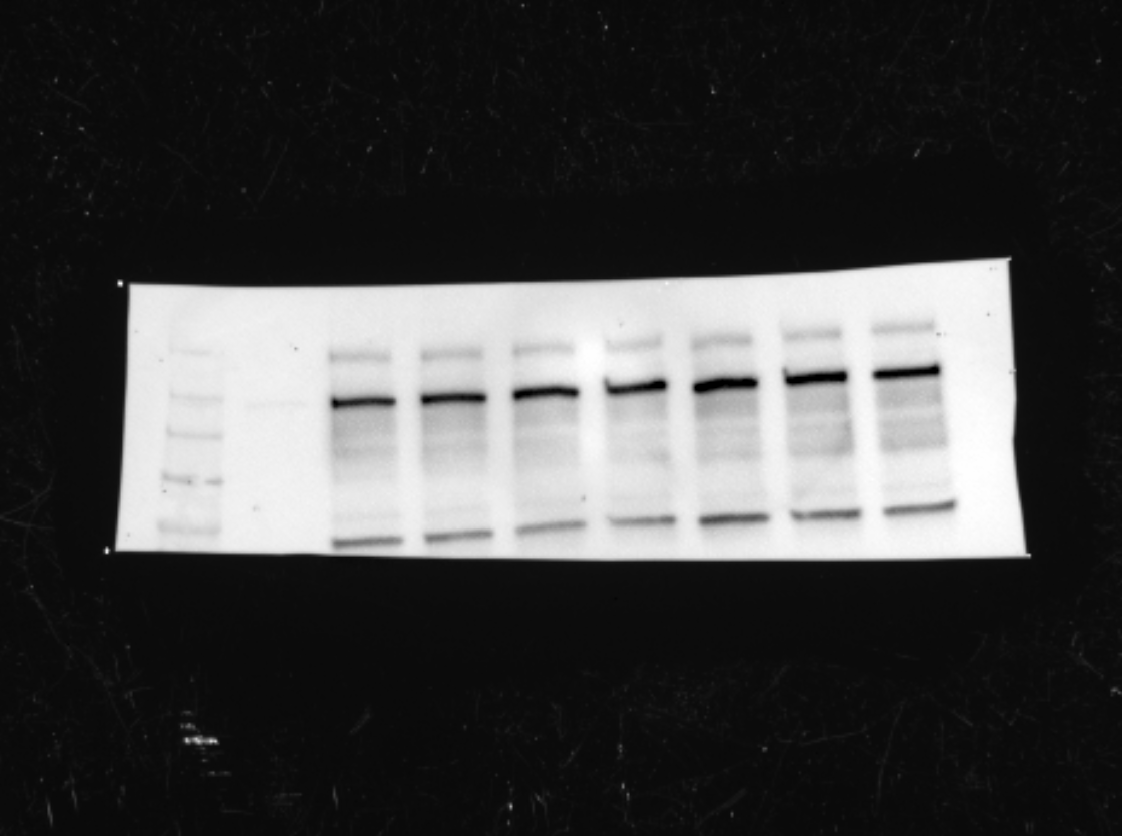

Supplement: Figure 2—source data 1. [file elife-85595-fig2-data1.zip › Figure 2-source data 1/Original_files/Figure 2D_Myc_input.tif]

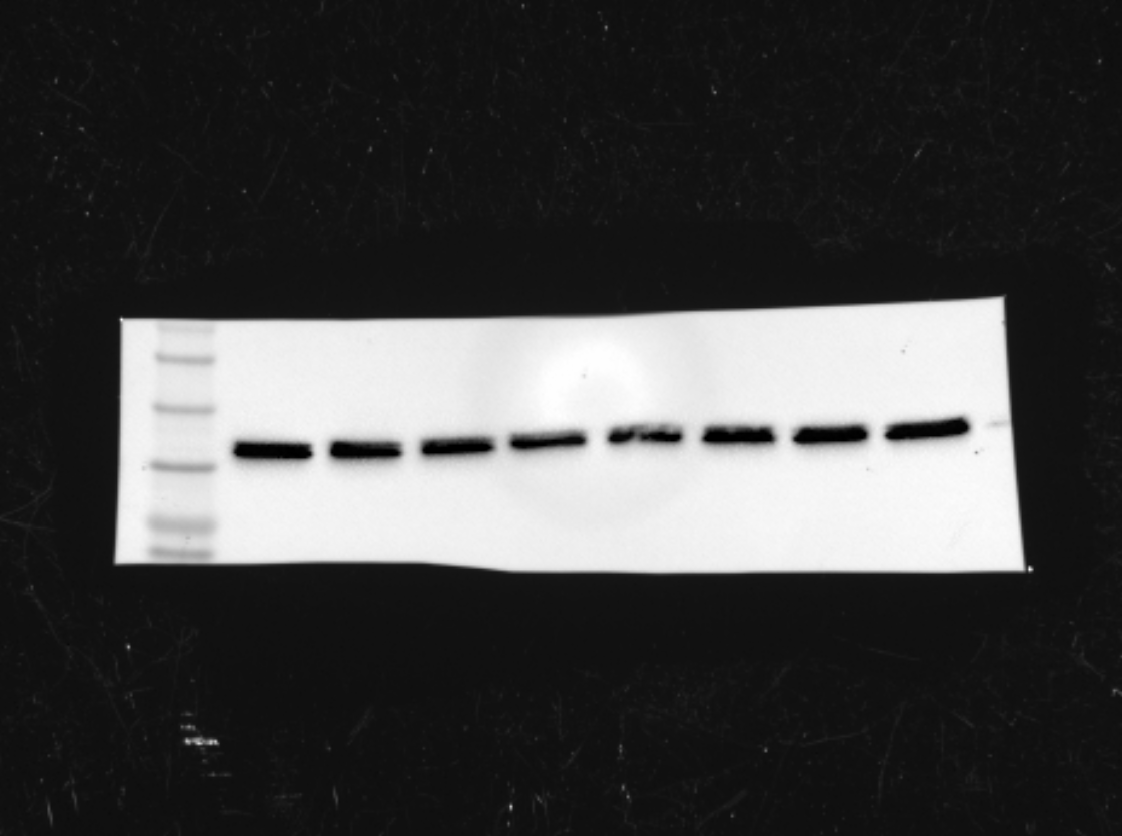

Supplement: Figure 2—source data 1. [file elife-85595-fig2-data1.zip › Figure 2-source data 1/Original_files/Figure 2D_GAPDH_input.tif]

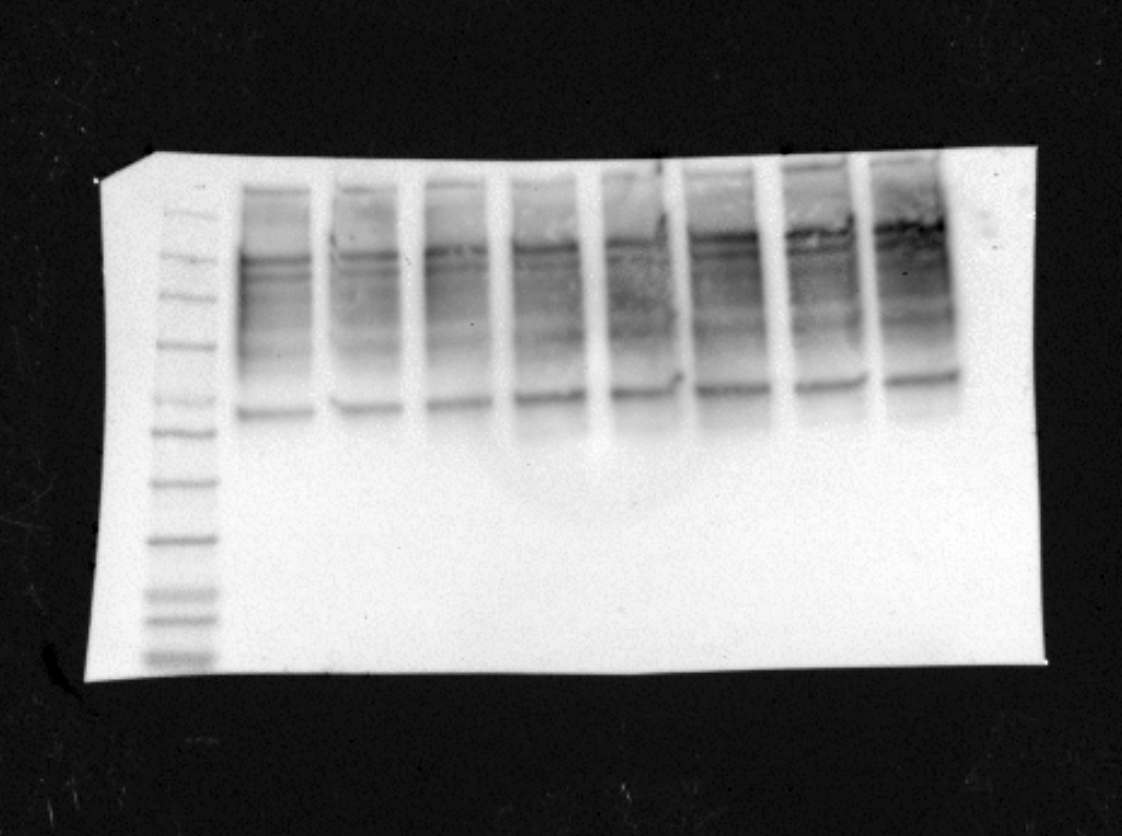

Supplement: Figure 2—source data 1. [file elife-85595-fig2-data1.zip › Figure 2-source data 1/Original_files/Figure 2D_O-GlcNAc_input.tif]

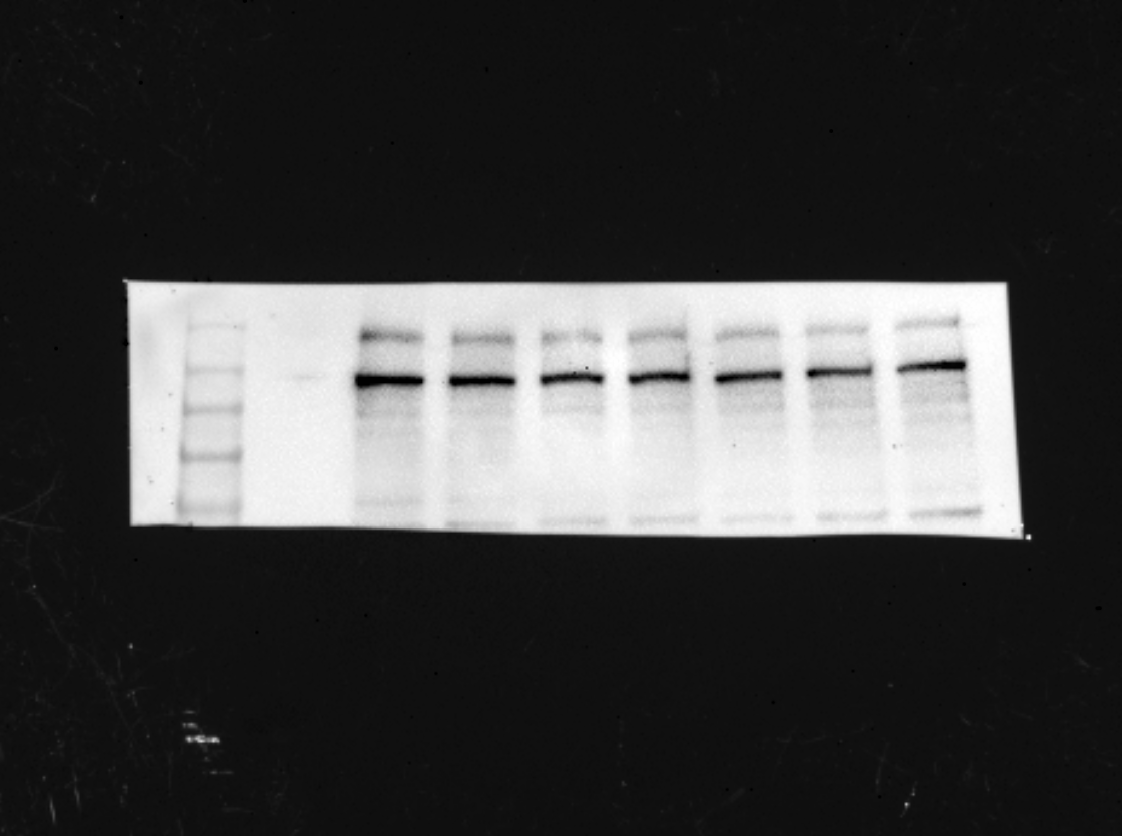

Supplement: Figure 2—source data 1. [file elife-85595-fig2-data1.zip › Figure 2-source data 1/Original_files/Figure 2D_DNMT1_IP.tif]

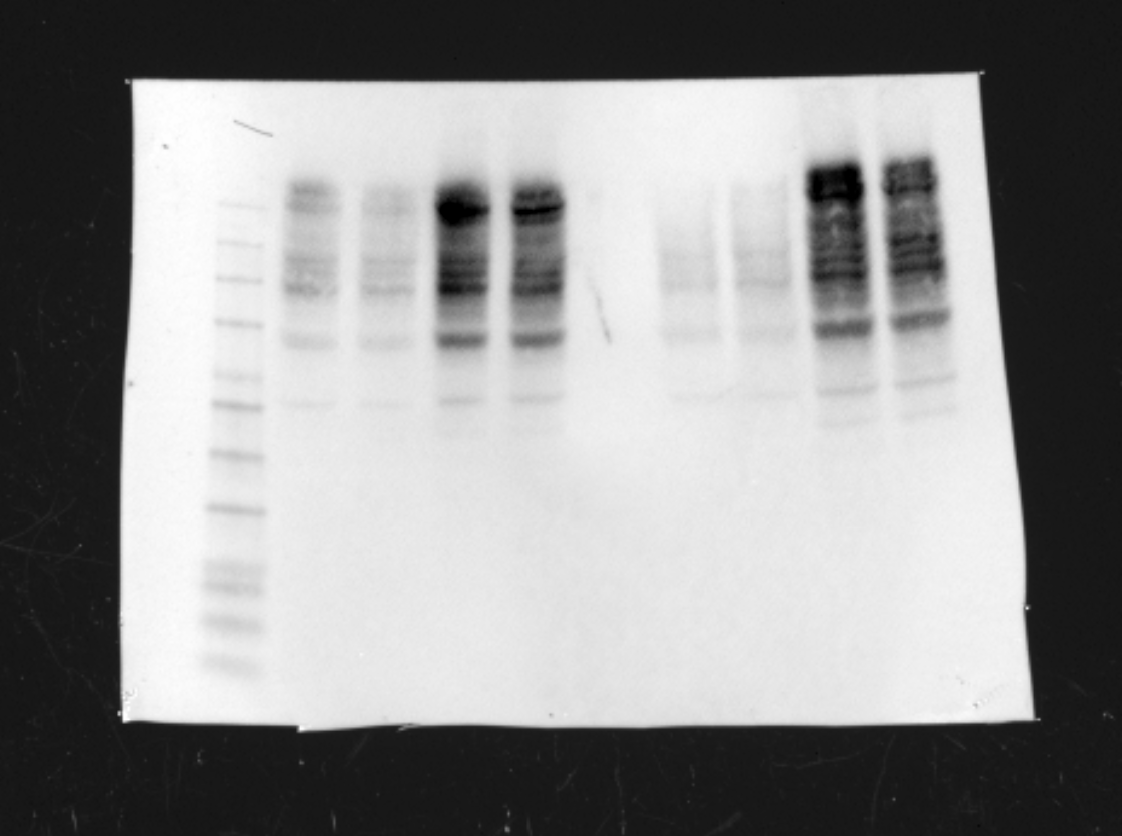

Supplement: Figure 2—figure supplement 1—source data 1. [file elife-85595-fig2-figsupp1-data1.zip › Figure 2-figure supplement 1-source data 1/Original_files/Figure 2-figure supplement 1_O-GlcNAc_input.tif]

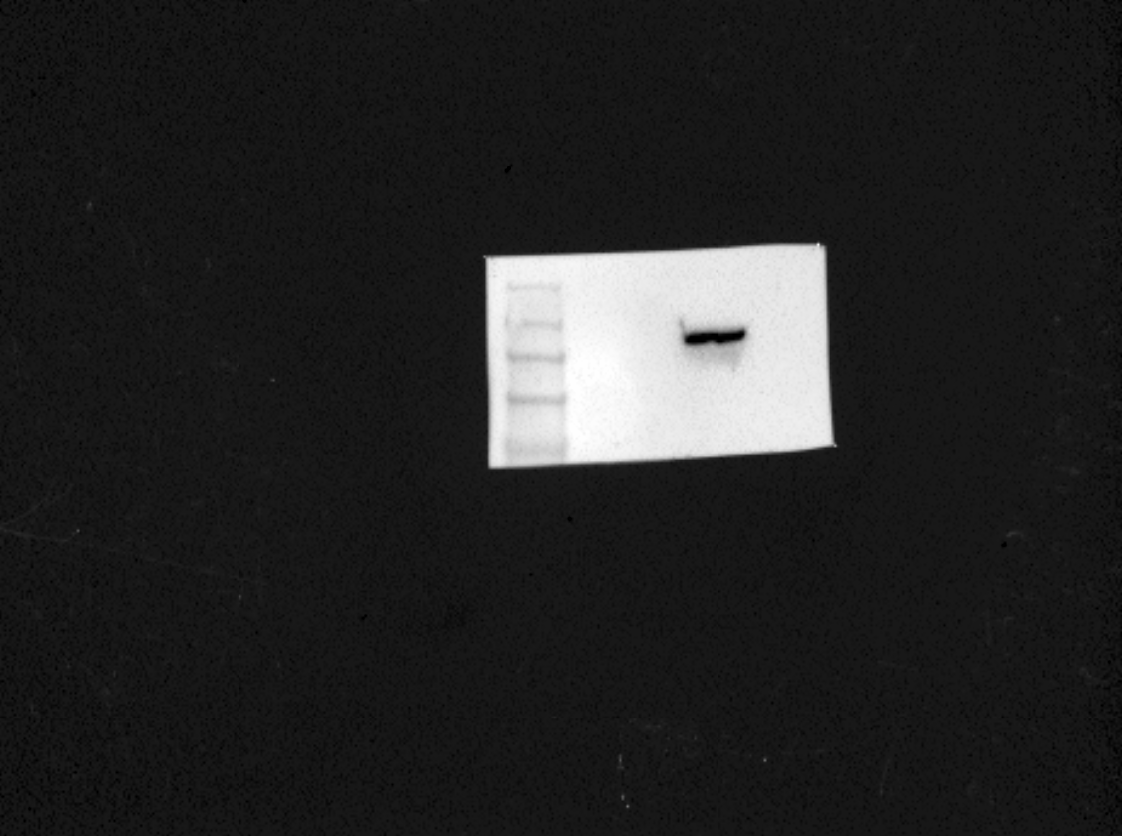

Supplement: Figure 2—figure supplement 1—source data 1. [file elife-85595-fig2-figsupp1-data1.zip › Figure 2-figure supplement 1-source data 1/Original_files/Figure 2-figure supplement 1_myc_input.tif]

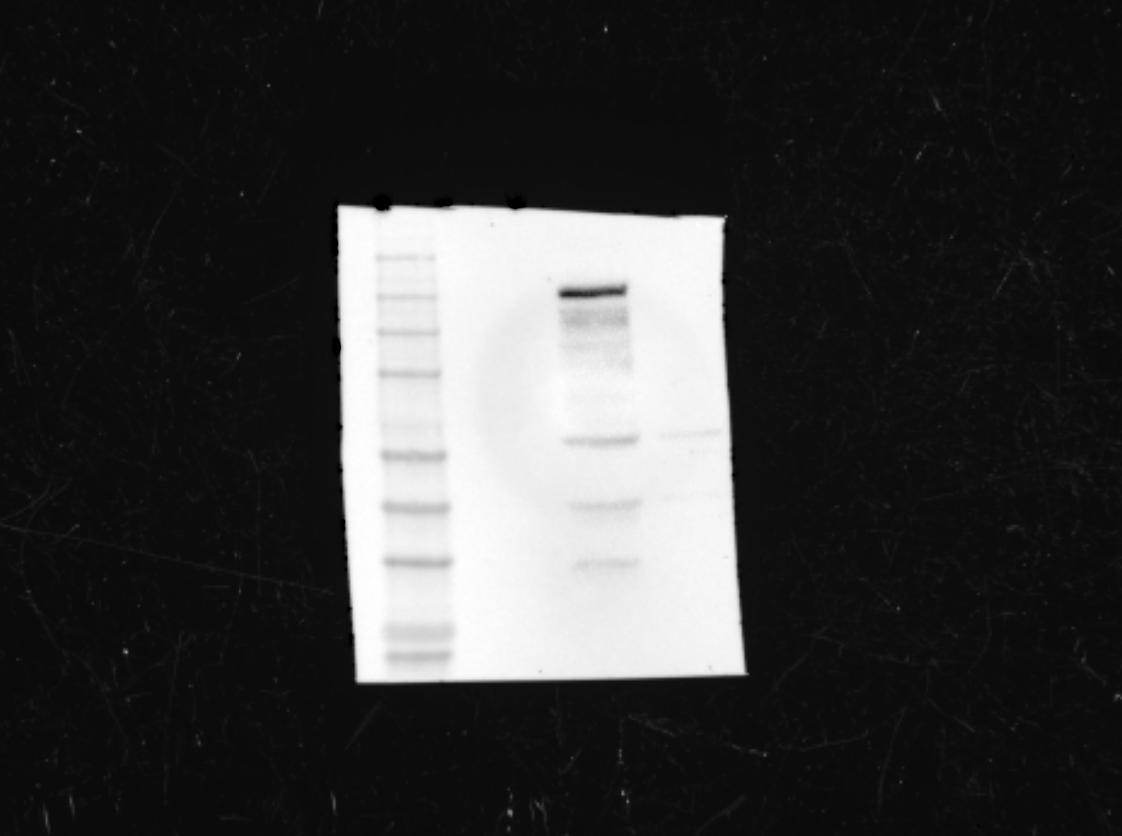

Supplement: Figure 2—figure supplement 1—source data 1. [file elife-85595-fig2-figsupp1-data1.zip › Figure 2-figure supplement 1-source data 1/Original_files/Figure 2-figure supplement 1_myc_IP.tif]

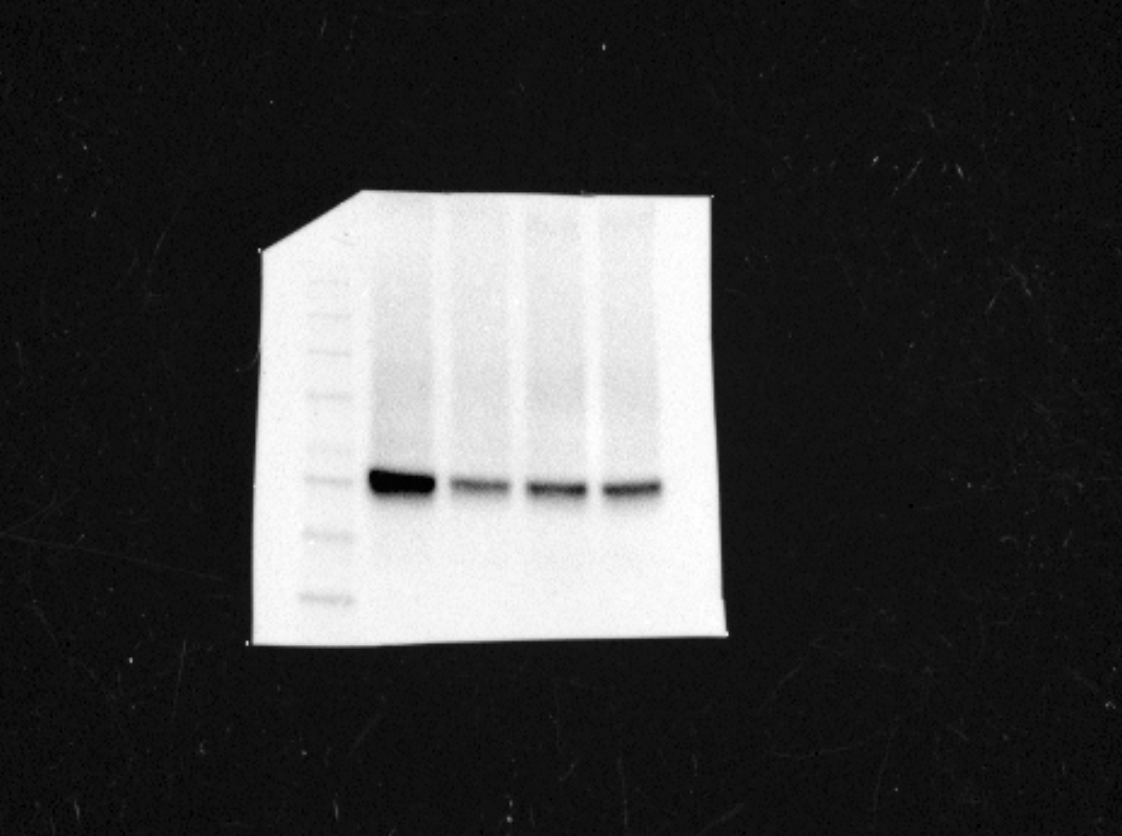

Supplement: Figure 2—figure supplement 1—source data 1. [file elife-85595-fig2-figsupp1-data1.zip › Figure 2-figure supplement 1-source data 1/Original_files/Figure 2-figure supplement 1_Tubulin_input.tif]

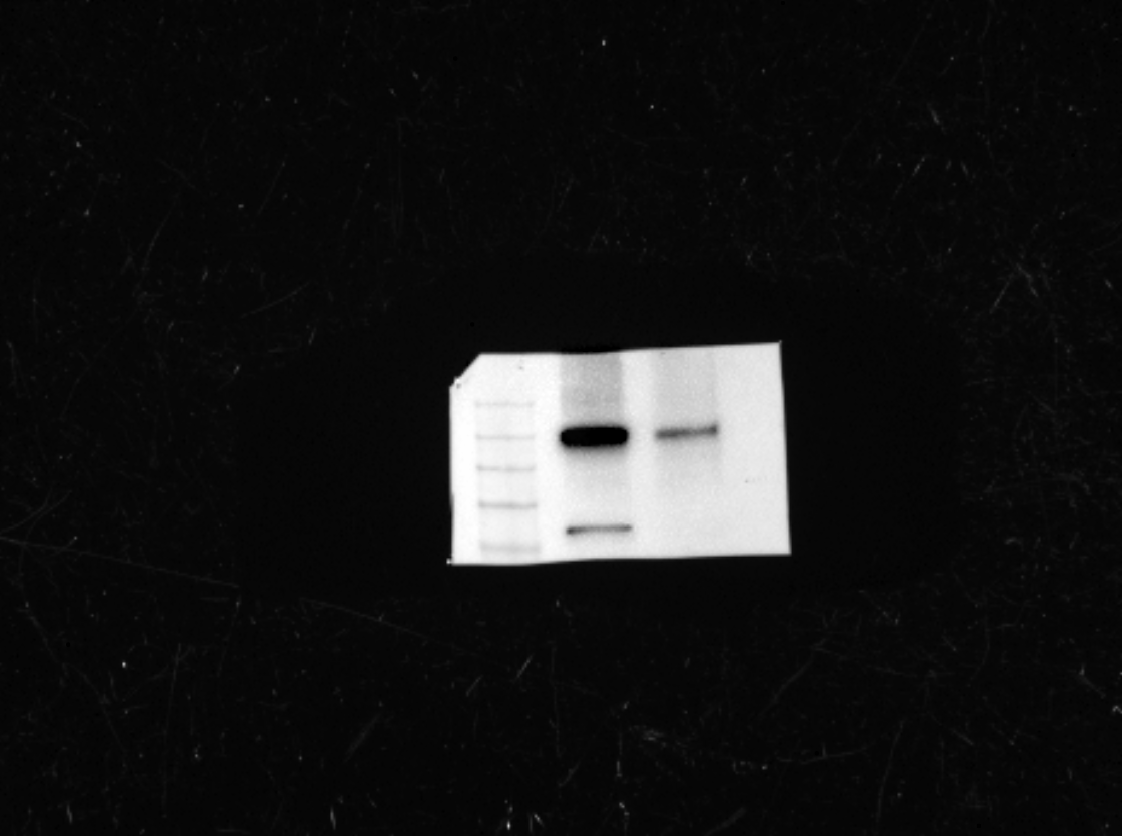

Supplement: Figure 2—figure supplement 1—source data 1. [file elife-85595-fig2-figsupp1-data1.zip › Figure 2-figure supplement 1-source data 1/Original_files/Figure 2-figure supplement 1_DNMT1_input.tif]

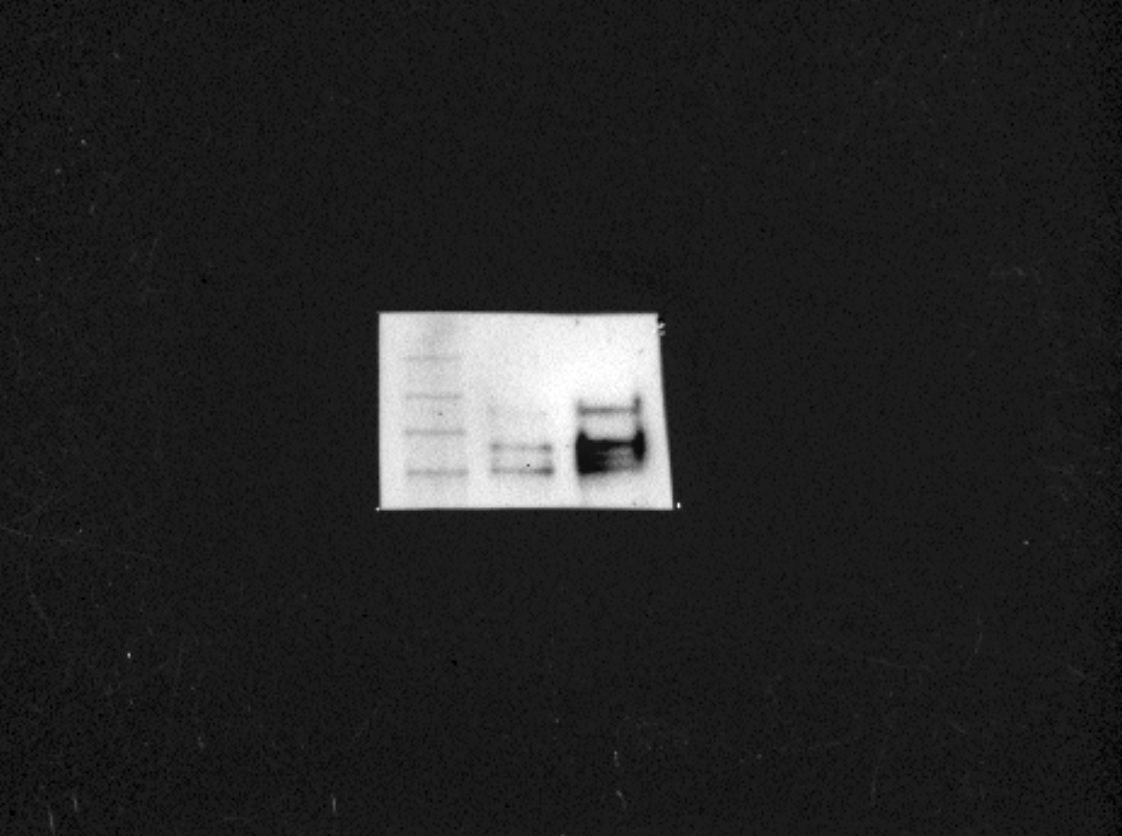

Supplement: Figure 2—figure supplement 1—source data 1. [file elife-85595-fig2-figsupp1-data1.zip › Figure 2-figure supplement 1-source data 1/Original_files/Figure 2-figure supplement 1_O-GlcNAc_IP.tif]

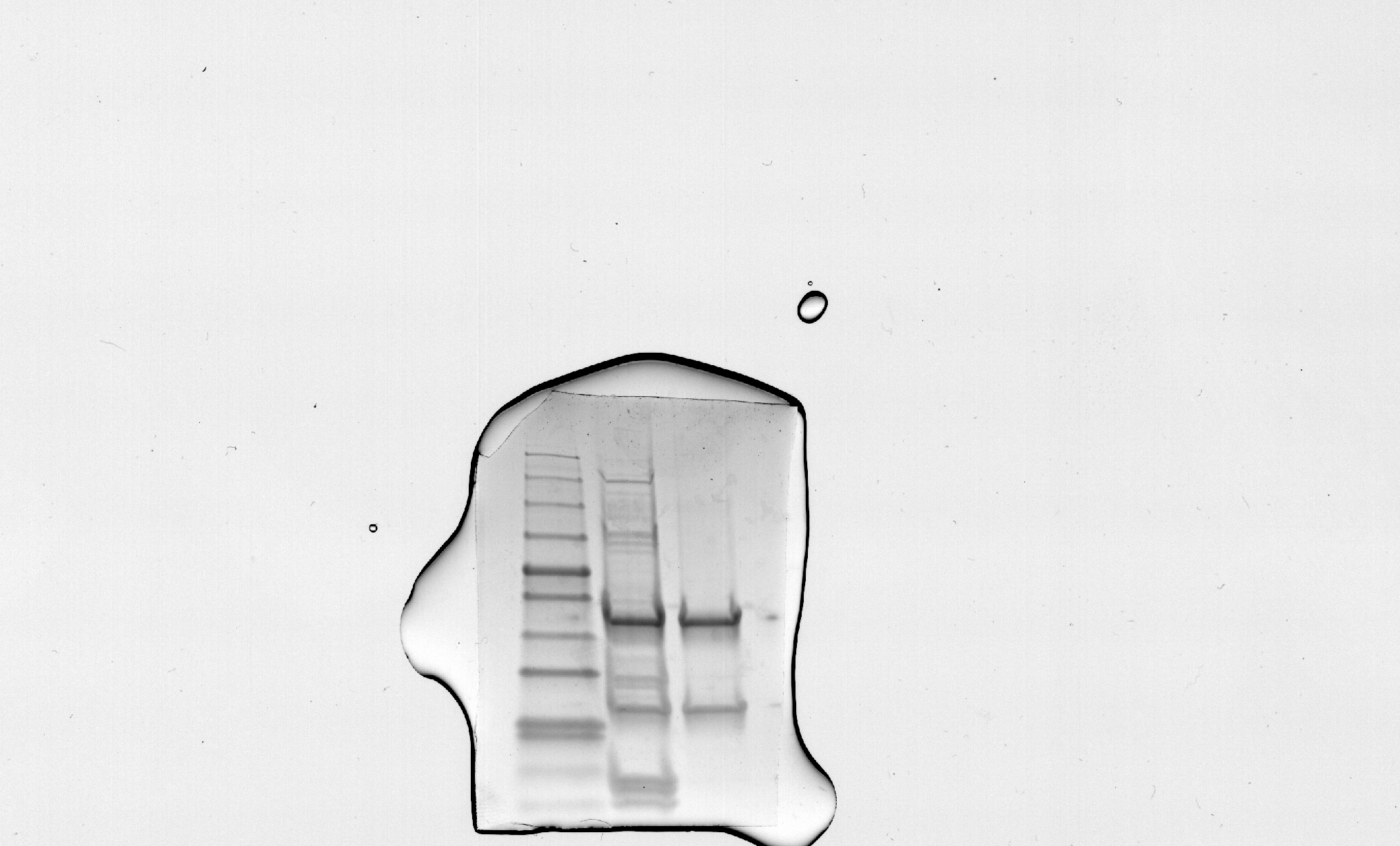

Supplement: Figure 2—figure supplement 1—source data 1. [file elife-85595-fig2-figsupp1-data1.zip › Figure 2-figure supplement 1-source data 1/Original_files/Figure 2-figure supplement 1_CBB_IP.tif]

Figure 3—figure supplement 3B

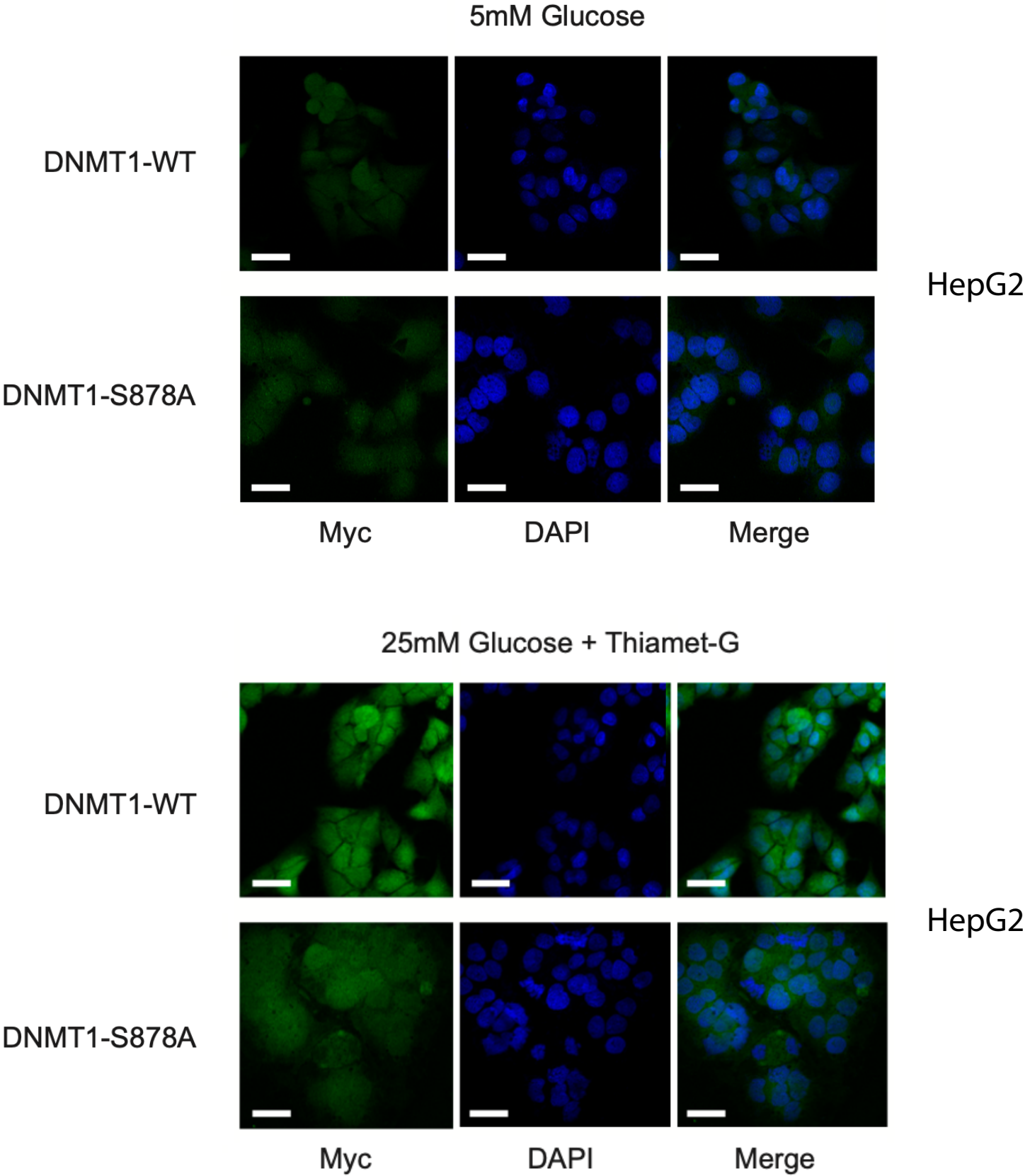

Supplement: Figure 3—figure supplement 3—source data 1. [file elife-85595-fig3-figsupp3-data1.zip › Figure 3-figure supplement 3-source data 1/Labeled_file/Figure 3-figure supplement 3B-source data 1.pdf]

Figure 3—figure supplement 3A

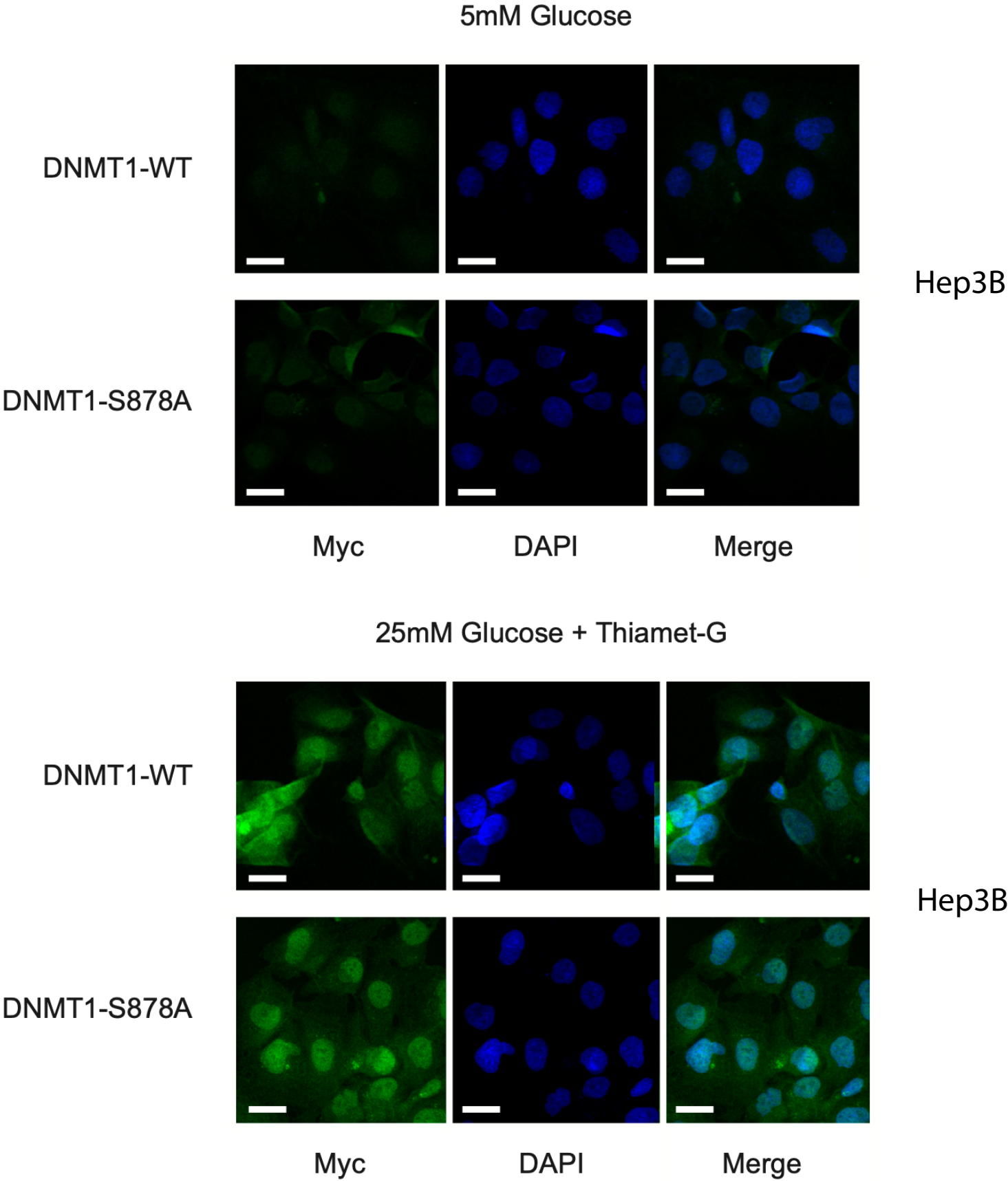

Supplement: Figure 3—figure supplement 3—source data 1. [file elife-85595-fig3-figsupp3-data1.zip › Figure 3-figure supplement 3-source data 1/Labeled_file/Figure 3-figure supplement 3A-source data 1.pdf]

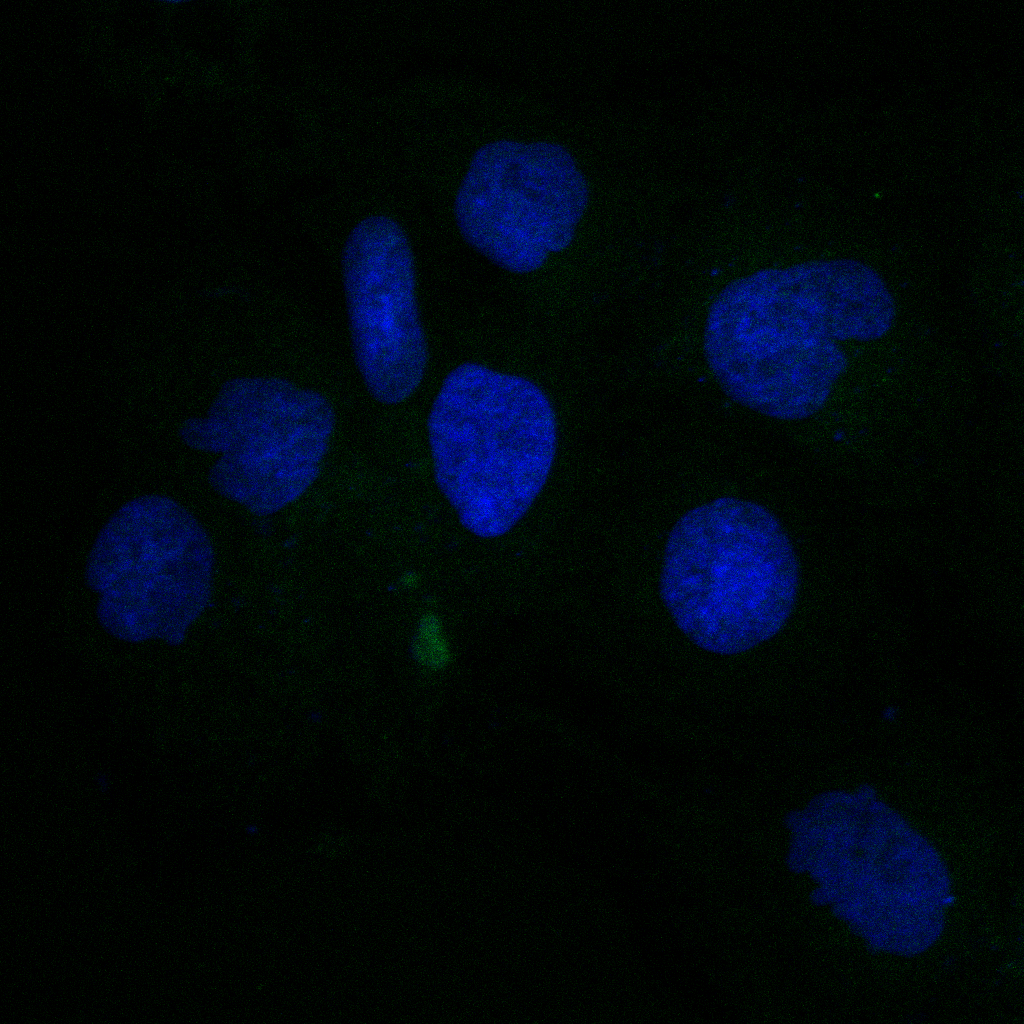

Supplement: Figure 3—figure supplement 3—source data 1. [file elife-85595-fig3-figsupp3-data1.zip › Figure 3-figure supplement 3-source data 1/Original_files/Figure 3-figure supplement 3A_WT_CTRL_merge.tif]

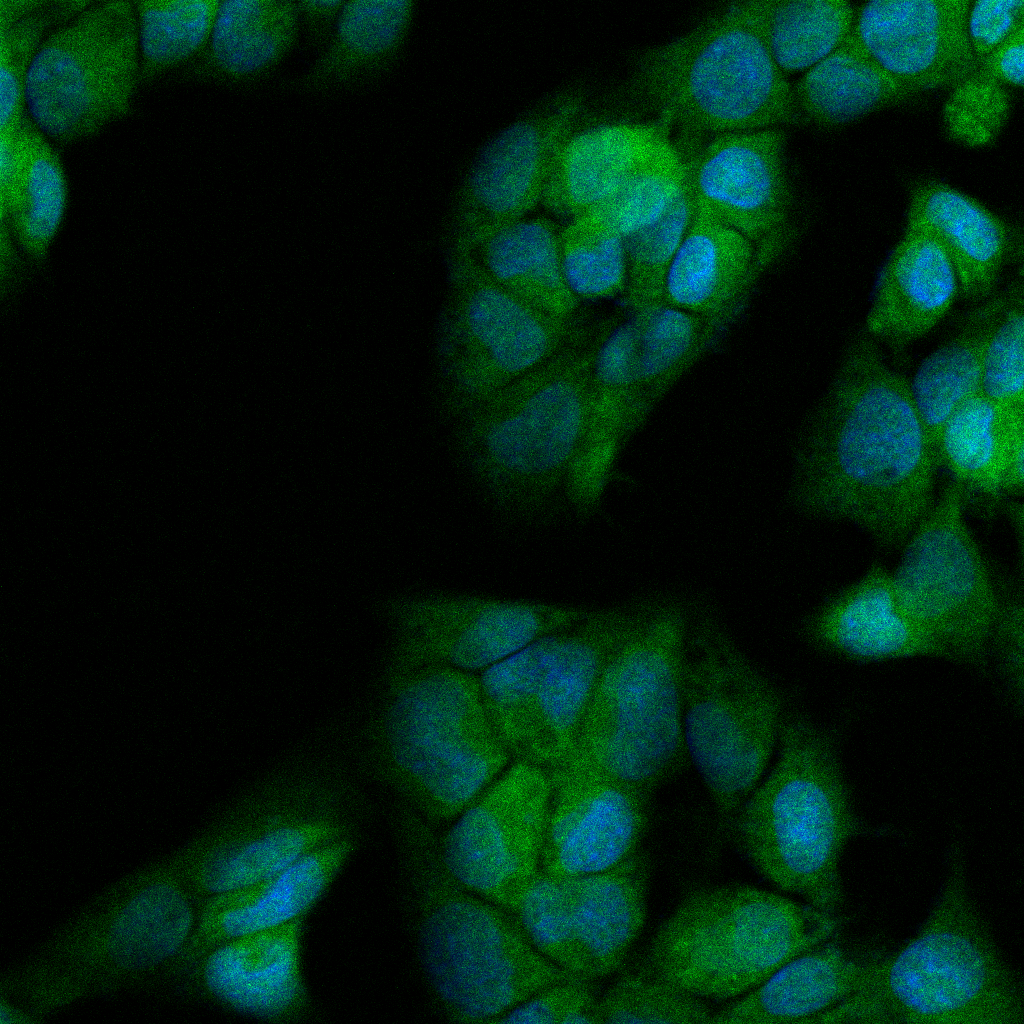

Supplement: Figure 3—figure supplement 3—source data 1. [file elife-85595-fig3-figsupp3-data1.zip › Figure 3-figure supplement 3-source data 1/Original_files/Figure 3-figure supplement 3B_WT_O-GlcNAc_merge.tif]

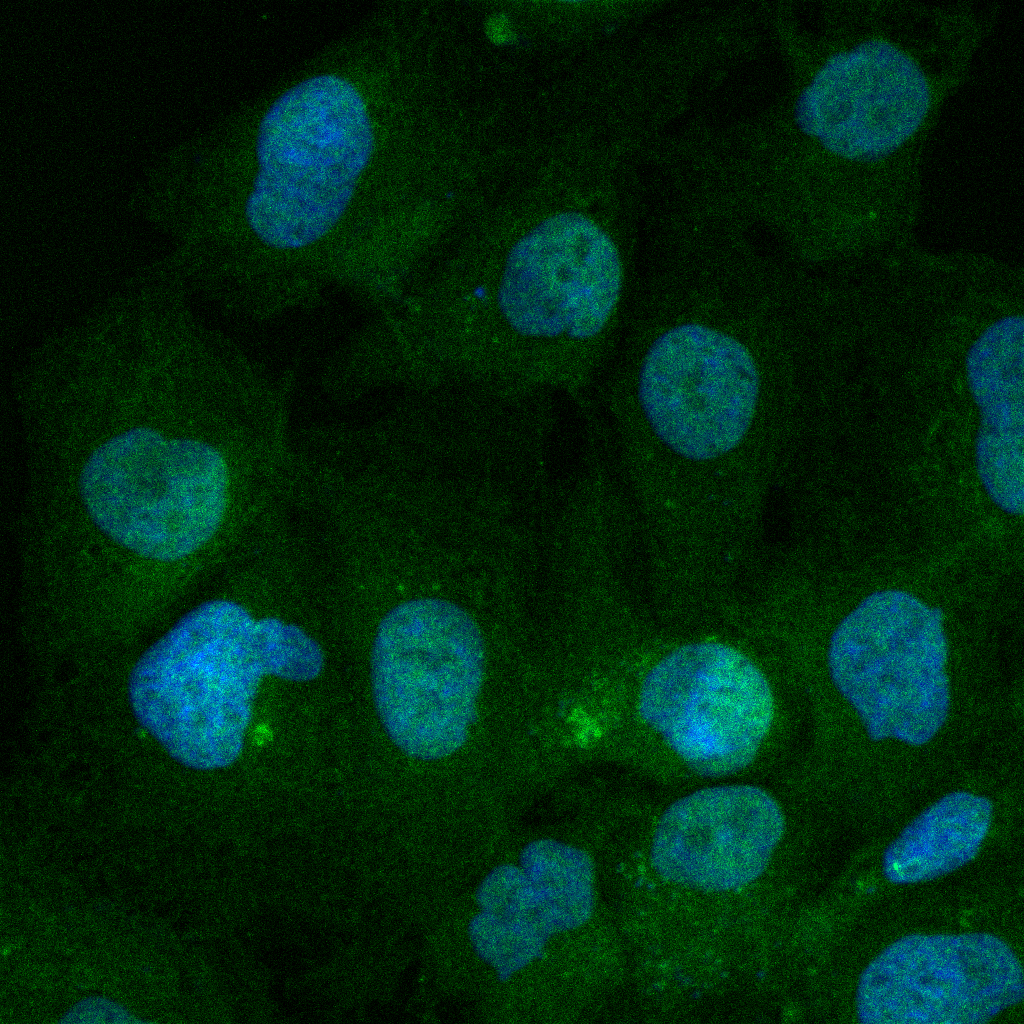

Supplement: Figure 3—figure supplement 3—source data 1. [file elife-85595-fig3-figsupp3-data1.zip › Figure 3-figure supplement 3-source data 1/Original_files/Figure 3-figure supplement 3A_S878A_O-GlcNAc_merge.tif]

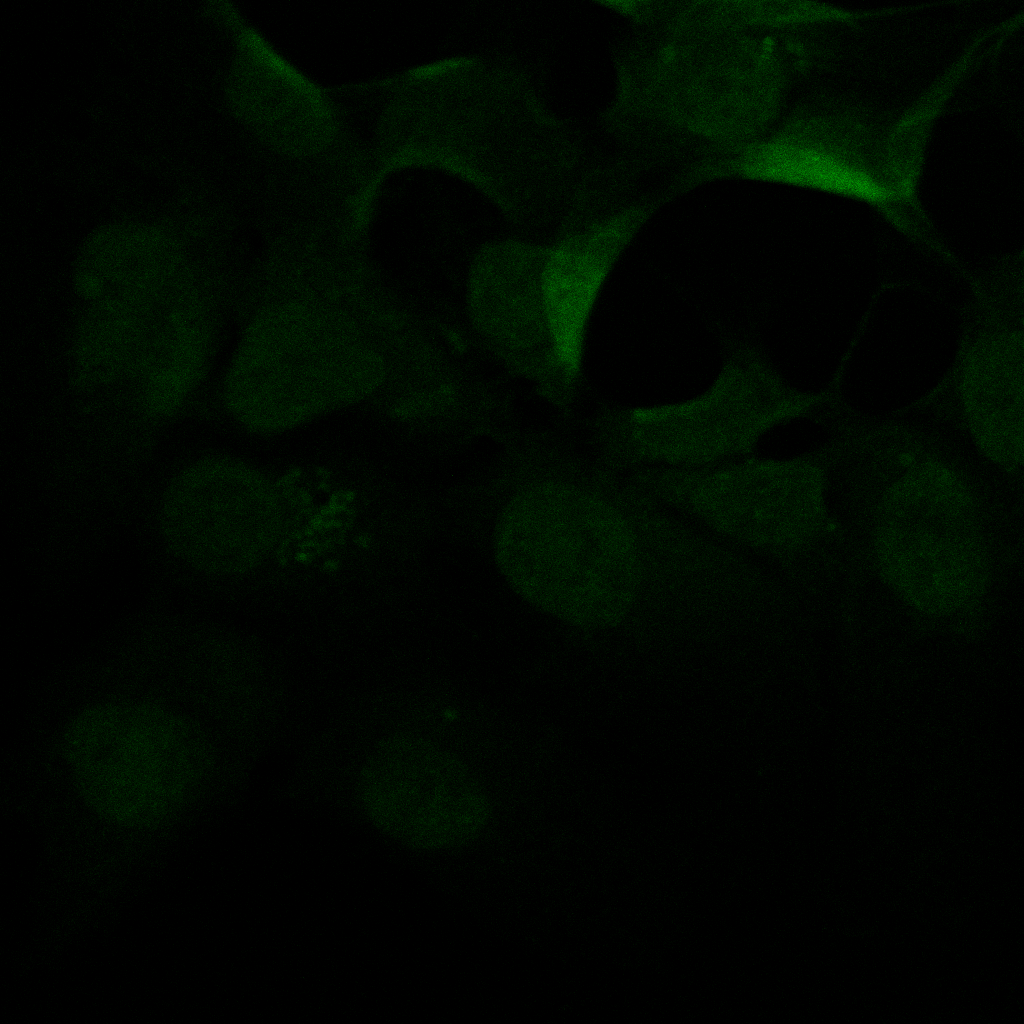

Supplement: Figure 3—figure supplement 3—source data 1. [file elife-85595-fig3-figsupp3-data1.zip › Figure 3-figure supplement 3-source data 1/Original_files/Figure 3-figure supplement 3A_S878A_CTRL_myc.tif]

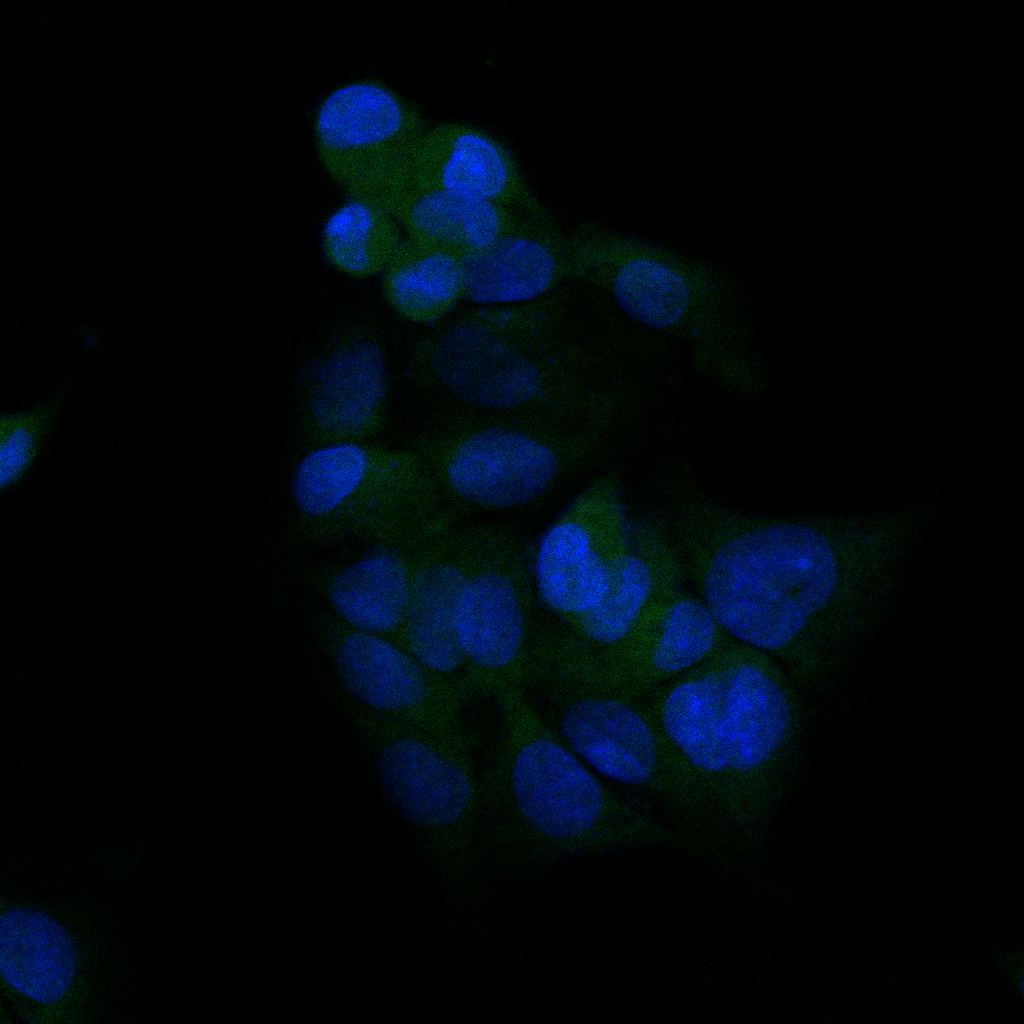

Supplement: Figure 3—figure supplement 3—source data 1. [file elife-85595-fig3-figsupp3-data1.zip › Figure 3-figure supplement 3-source data 1/Original_files/Figure 3-figure supplement 3B_WT_CTRL_merge.tif]

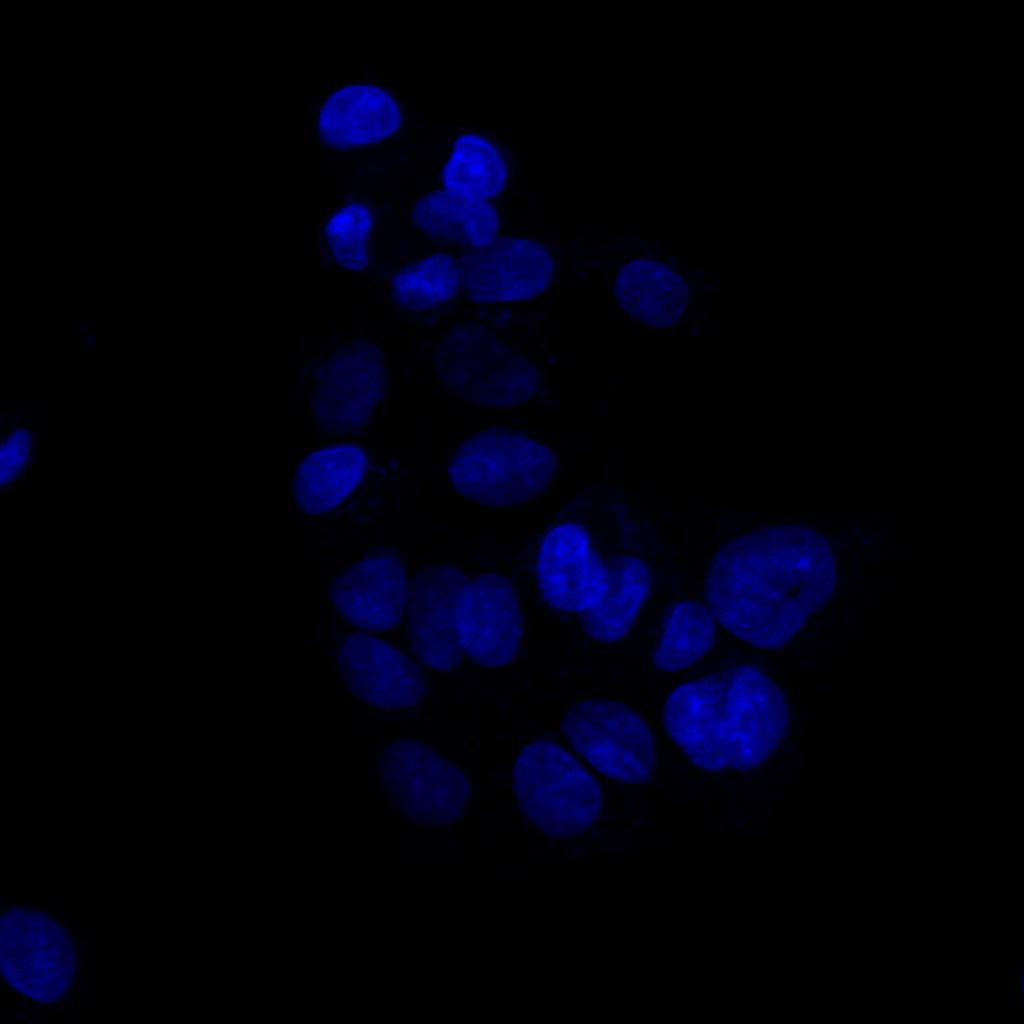

Supplement: Figure 3—figure supplement 3—source data 1. [file elife-85595-fig3-figsupp3-data1.zip › Figure 3-figure supplement 3-source data 1/Original_files/Figure 3-figure supplement 3B_WT_CTRL_DAPI.tif]

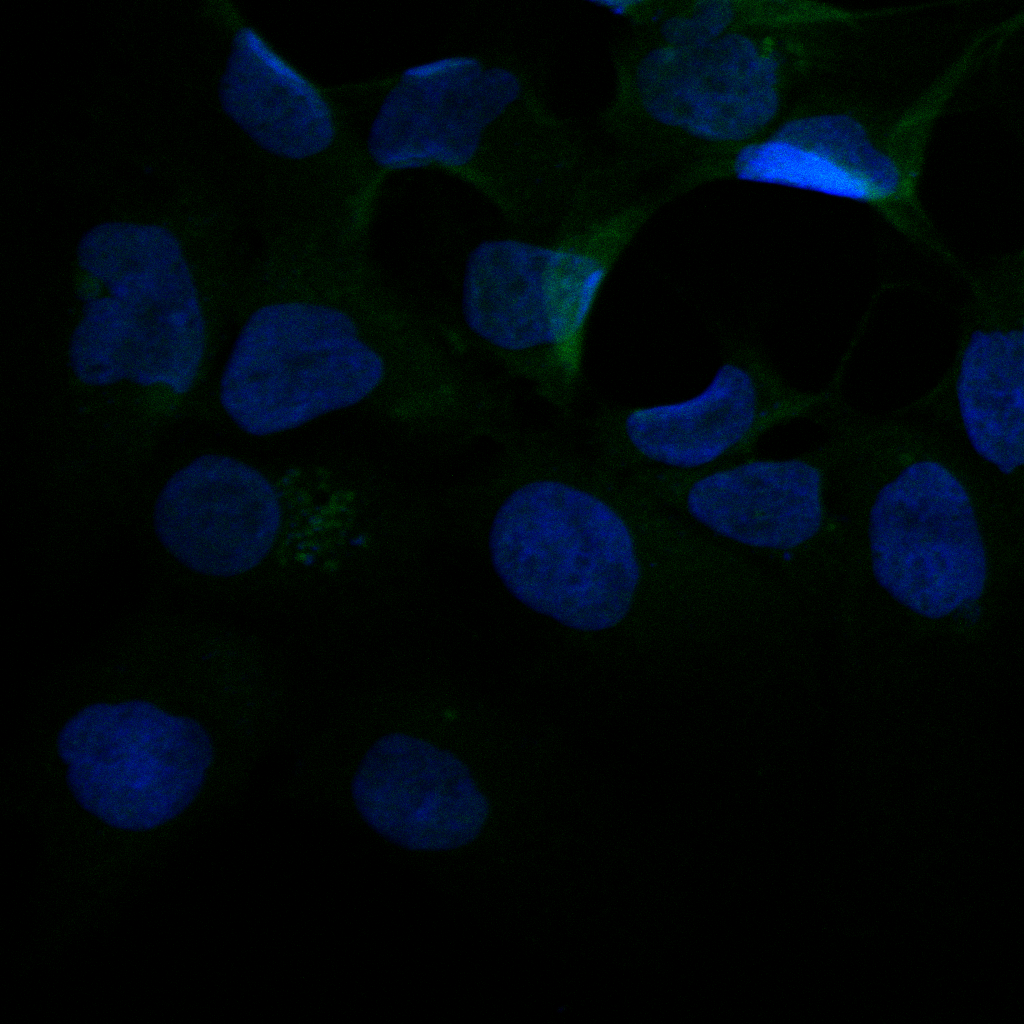

Supplement: Figure 3—figure supplement 3—source data 1. [file elife-85595-fig3-figsupp3-data1.zip › Figure 3-figure supplement 3-source data 1/Original_files/Figure 3-figure supplement 3A_S878A_CTRL_merge.tif]

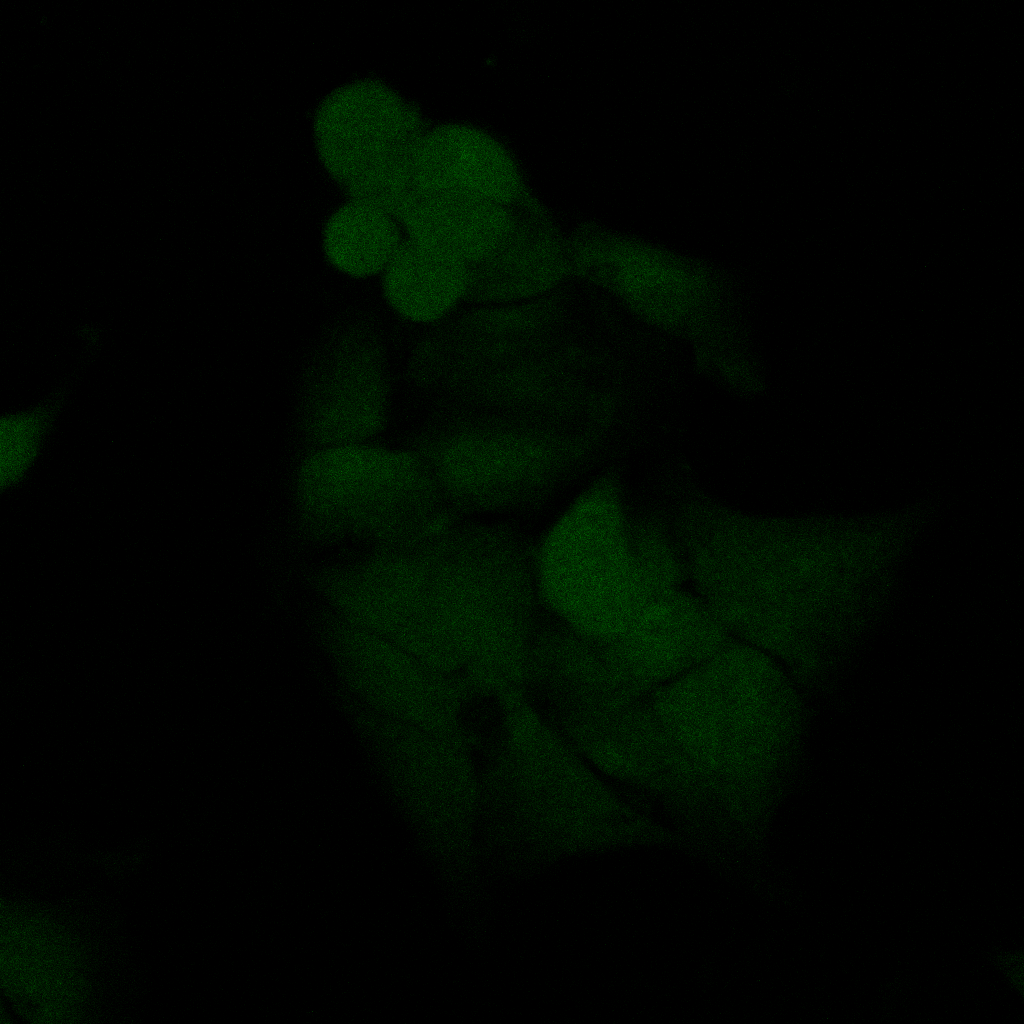

Supplement: Figure 3—figure supplement 3—source data 1. [file elife-85595-fig3-figsupp3-data1.zip › Figure 3-figure supplement 3-source data 1/Original_files/Figure 3-figure supplement 3B_WT_CTRL_myc.tif]

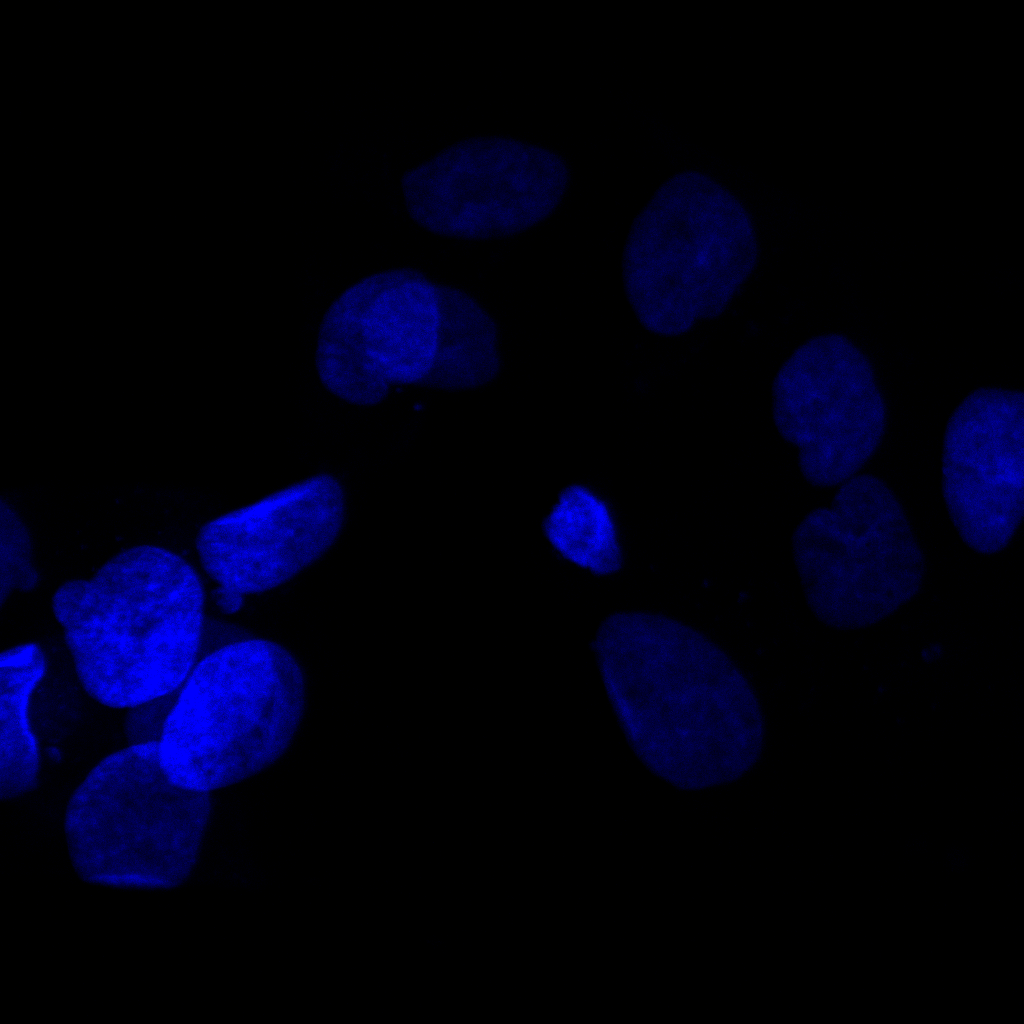

Supplement: Figure 3—figure supplement 3—source data 1. [file elife-85595-fig3-figsupp3-data1.zip › Figure 3-figure supplement 3-source data 1/Original_files/Figure 3-figure supplement 3A_WT_O-GlcNAc_DAPI.tif]

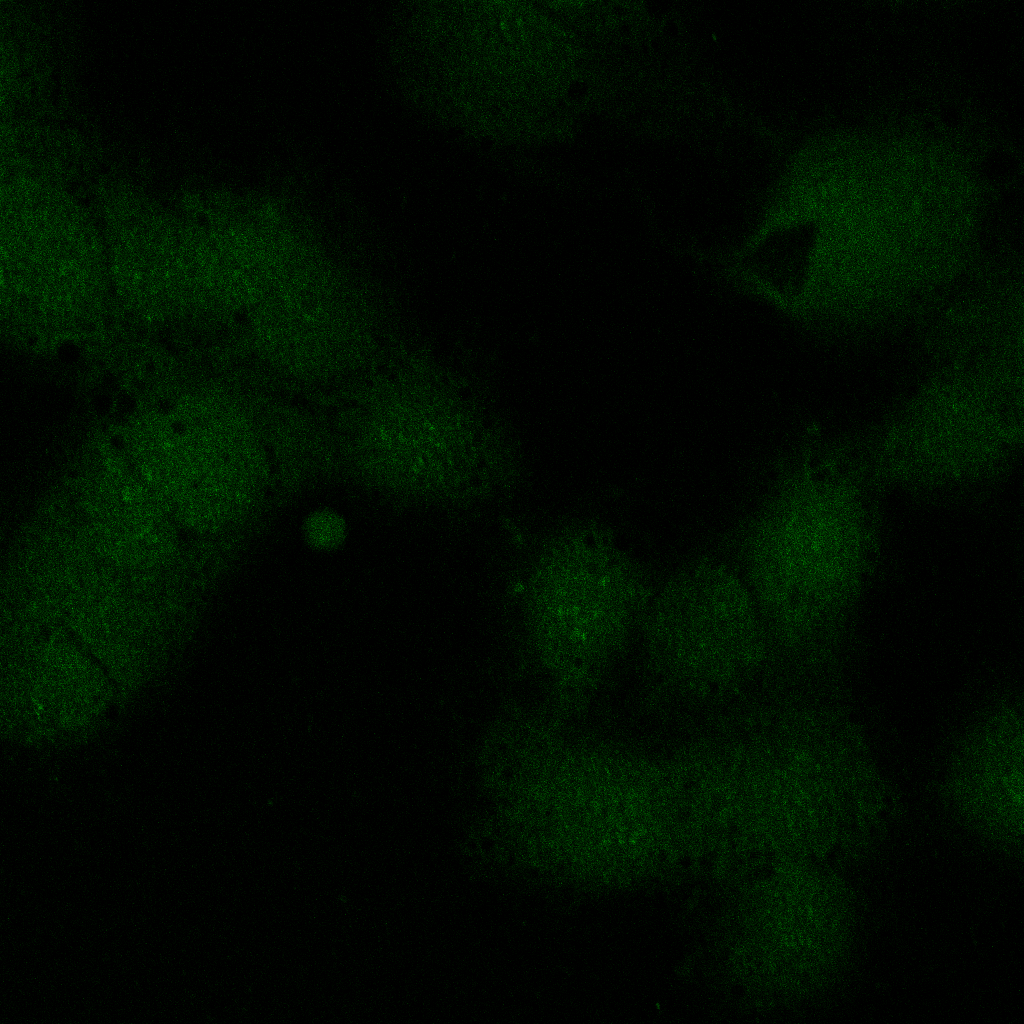

Supplement: Figure 3—figure supplement 3—source data 1. [file elife-85595-fig3-figsupp3-data1.zip › Figure 3-figure supplement 3-source data 1/Original_files/Figure 3-figure supplement 3B_S878A_CTRL_myc.tif]

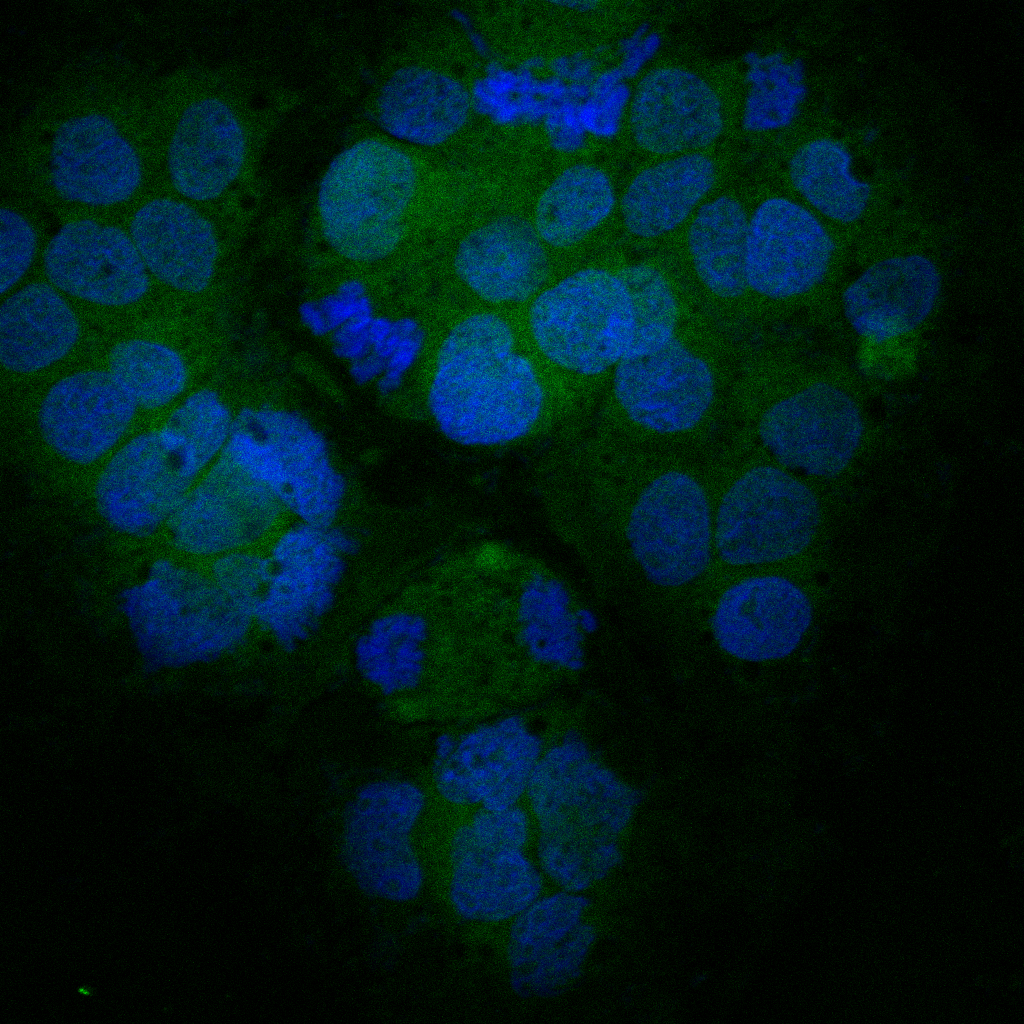

Supplement: Figure 3—figure supplement 3—source data 1. [file elife-85595-fig3-figsupp3-data1.zip › Figure 3-figure supplement 3-source data 1/Original_files/Figure 3-figure supplement 3B_S878A_O-GlcNAc_merge.tif]

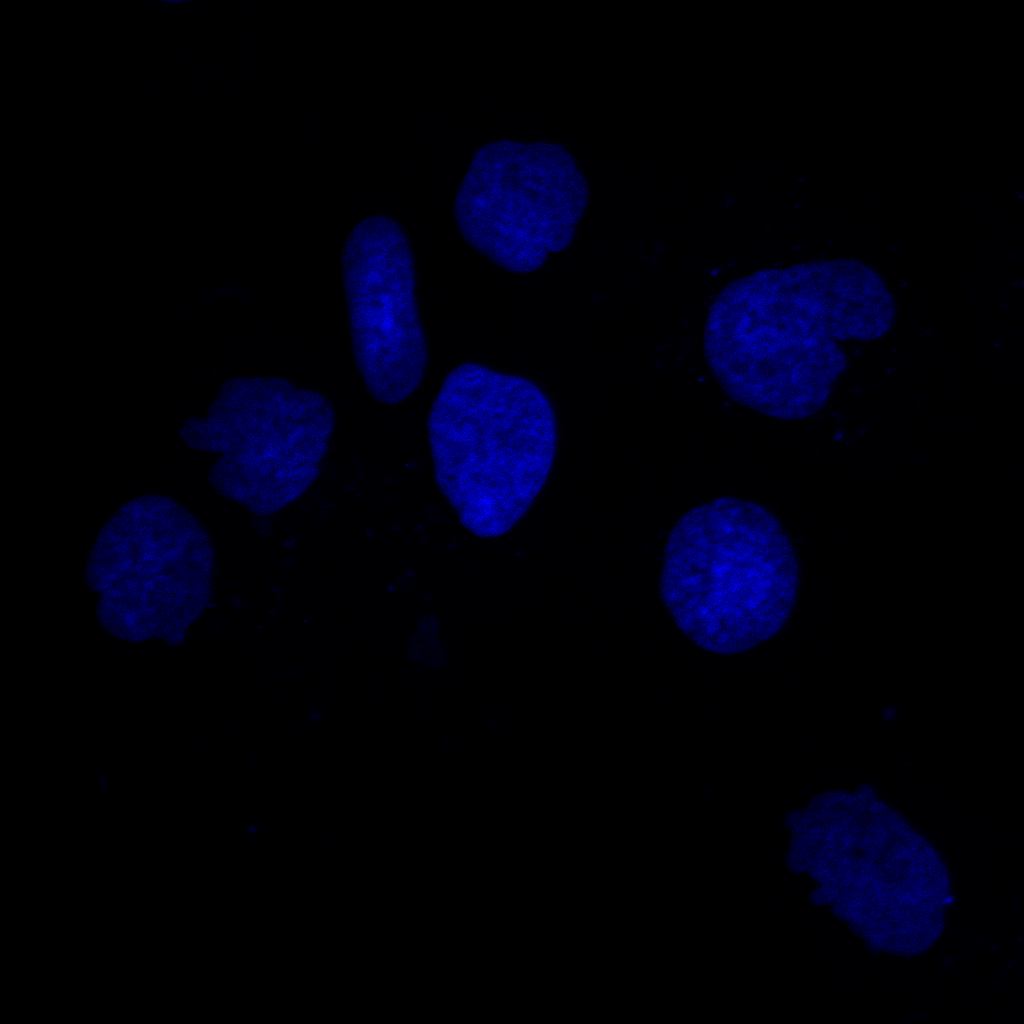

Supplement: Figure 3—figure supplement 3—source data 1. [file elife-85595-fig3-figsupp3-data1.zip › Figure 3-figure supplement 3-source data 1/Original_files/Figure 3-figure supplement 3A_WT_CTRL_DAPI.tif]

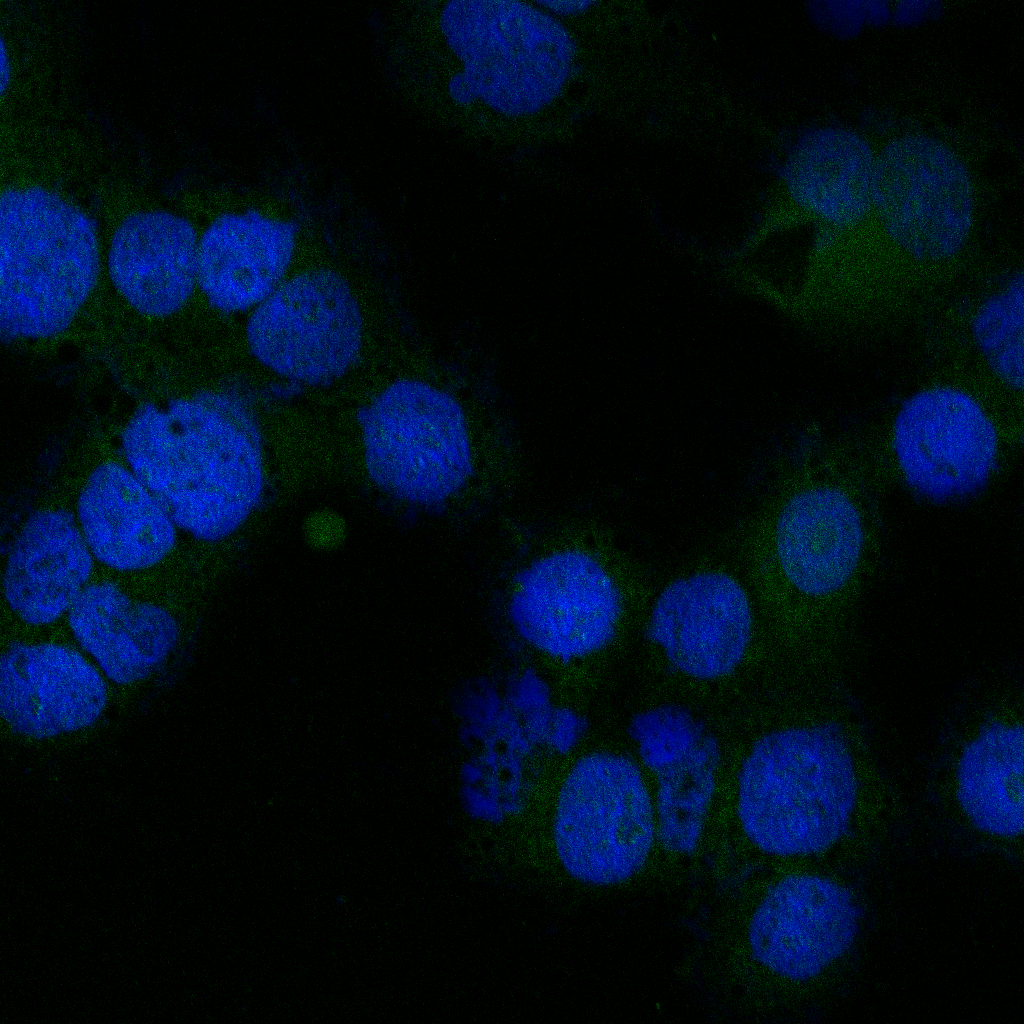

Supplement: Figure 3—figure supplement 3—source data 1. [file elife-85595-fig3-figsupp3-data1.zip › Figure 3-figure supplement 3-source data 1/Original_files/Figure 3-figure supplement 3B_S878A_CTRL_merge.tif]

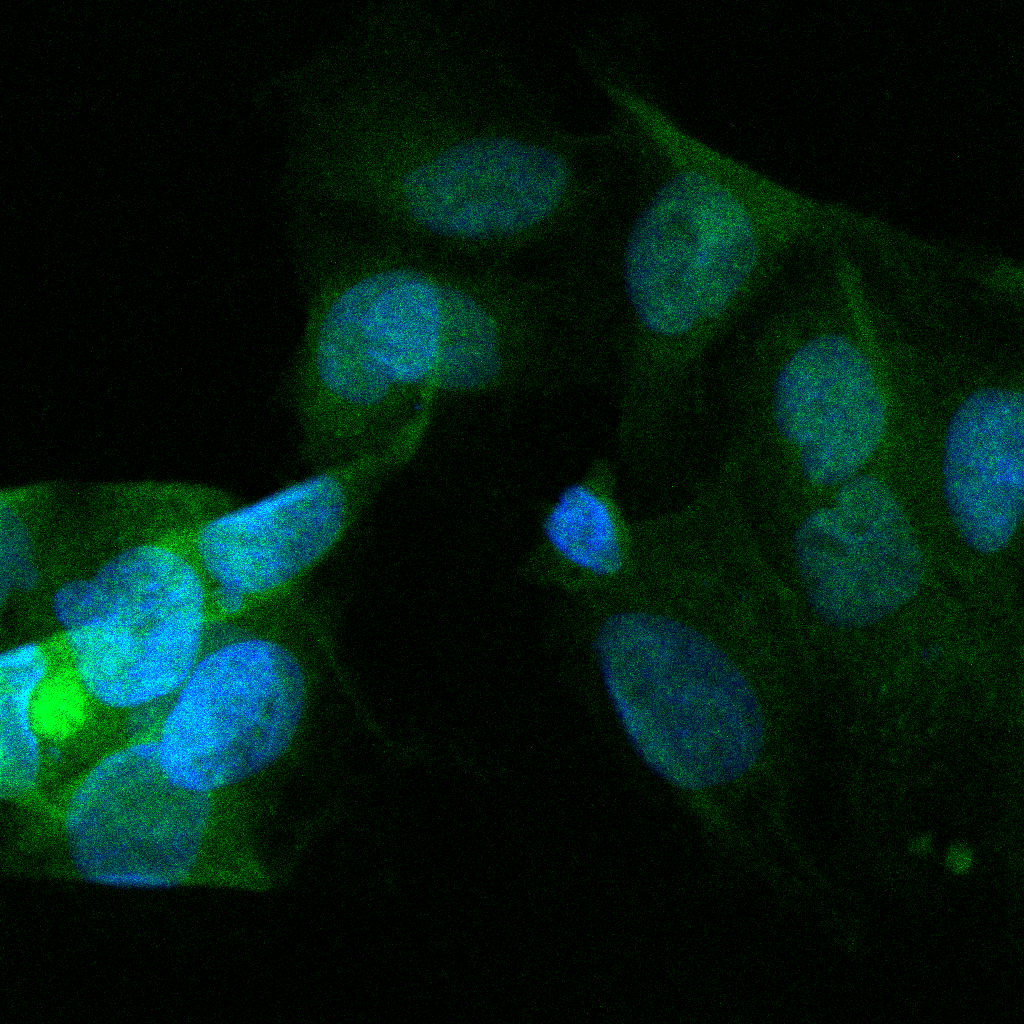

Supplement: Figure 3—figure supplement 3—source data 1. [file elife-85595-fig3-figsupp3-data1.zip › Figure 3-figure supplement 3-source data 1/Original_files/Figure 3-figure supplement 3A_WT_O-GlcNAc_merge.tif]

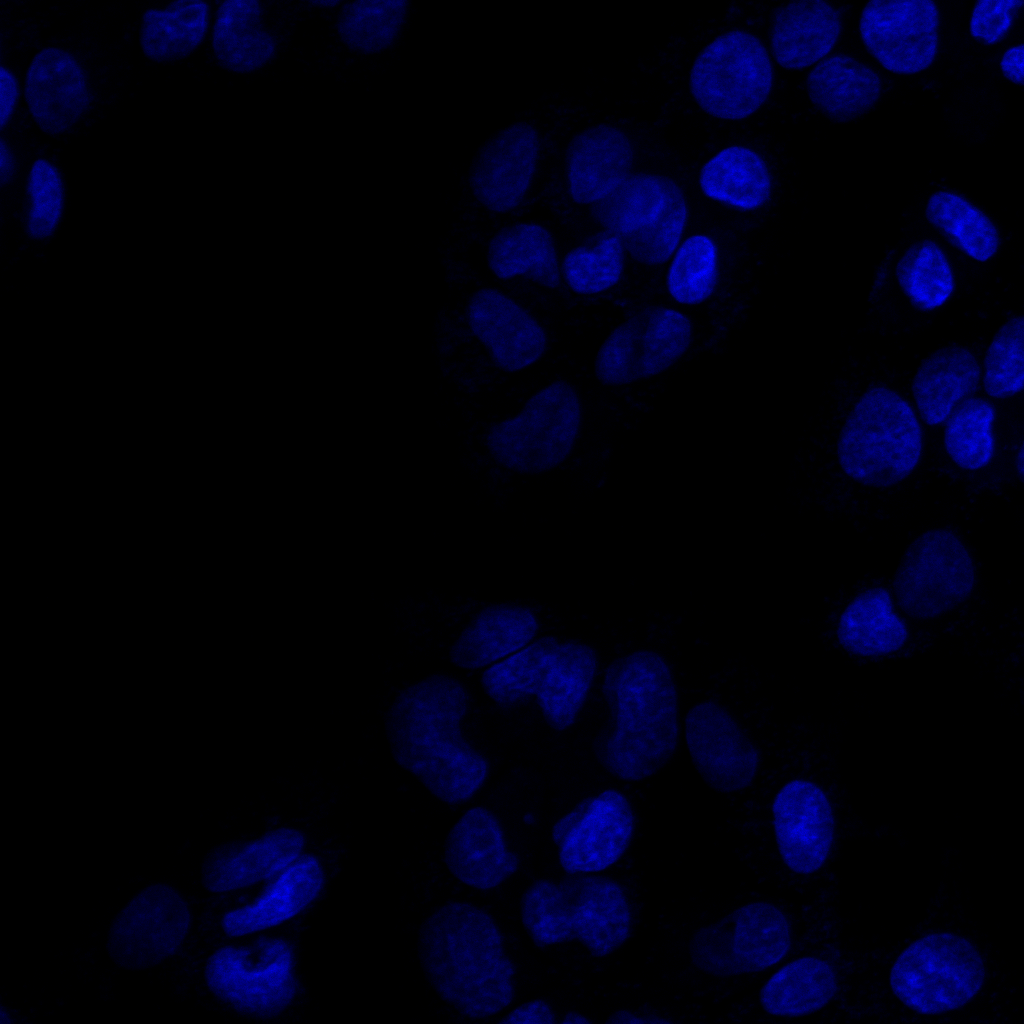

Supplement: Figure 3—figure supplement 3—source data 1. [file elife-85595-fig3-figsupp3-data1.zip › Figure 3-figure supplement 3-source data 1/Original_files/Figure 3-figure supplement 3B_WT_O-GlcNAc_DAPI.tif]

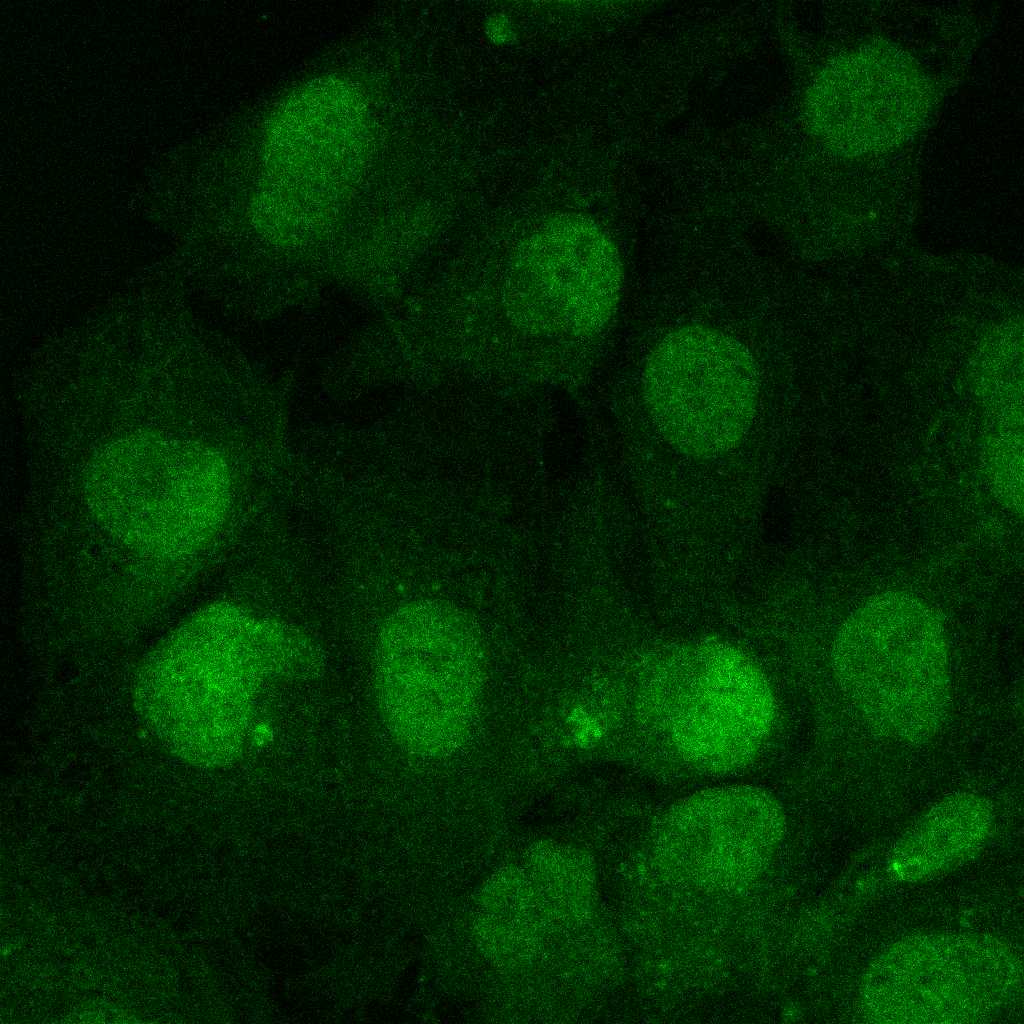

Supplement: Figure 3—figure supplement 3—source data 1. [file elife-85595-fig3-figsupp3-data1.zip › Figure 3-figure supplement 3-source data 1/Original_files/Figure 3-figure supplement 3A_S878A_O-GlcNAc_myc.tif]

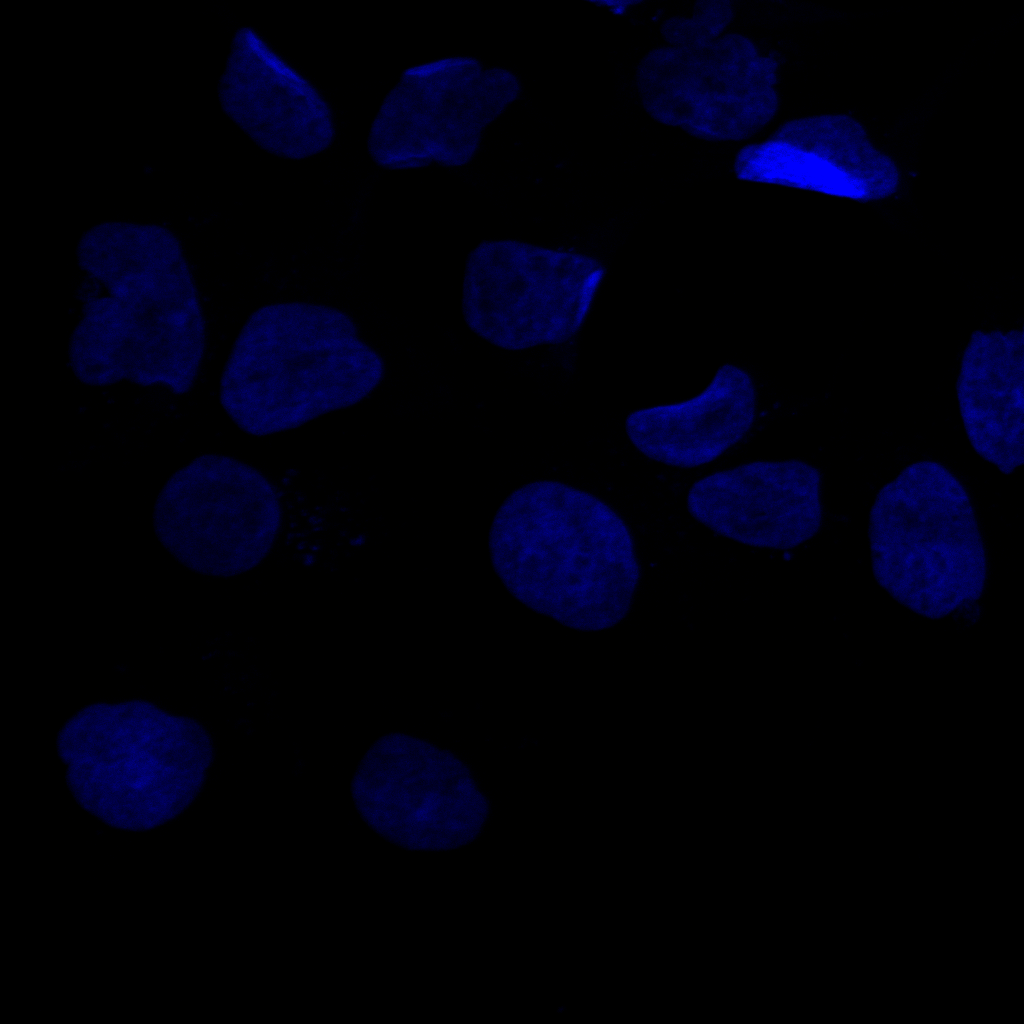

Supplement: Figure 3—figure supplement 3—source data 1. [file elife-85595-fig3-figsupp3-data1.zip › Figure 3-figure supplement 3-source data 1/Original_files/Figure 3-figure supplement 3A_S878A_CTRL_DAPI.tif]

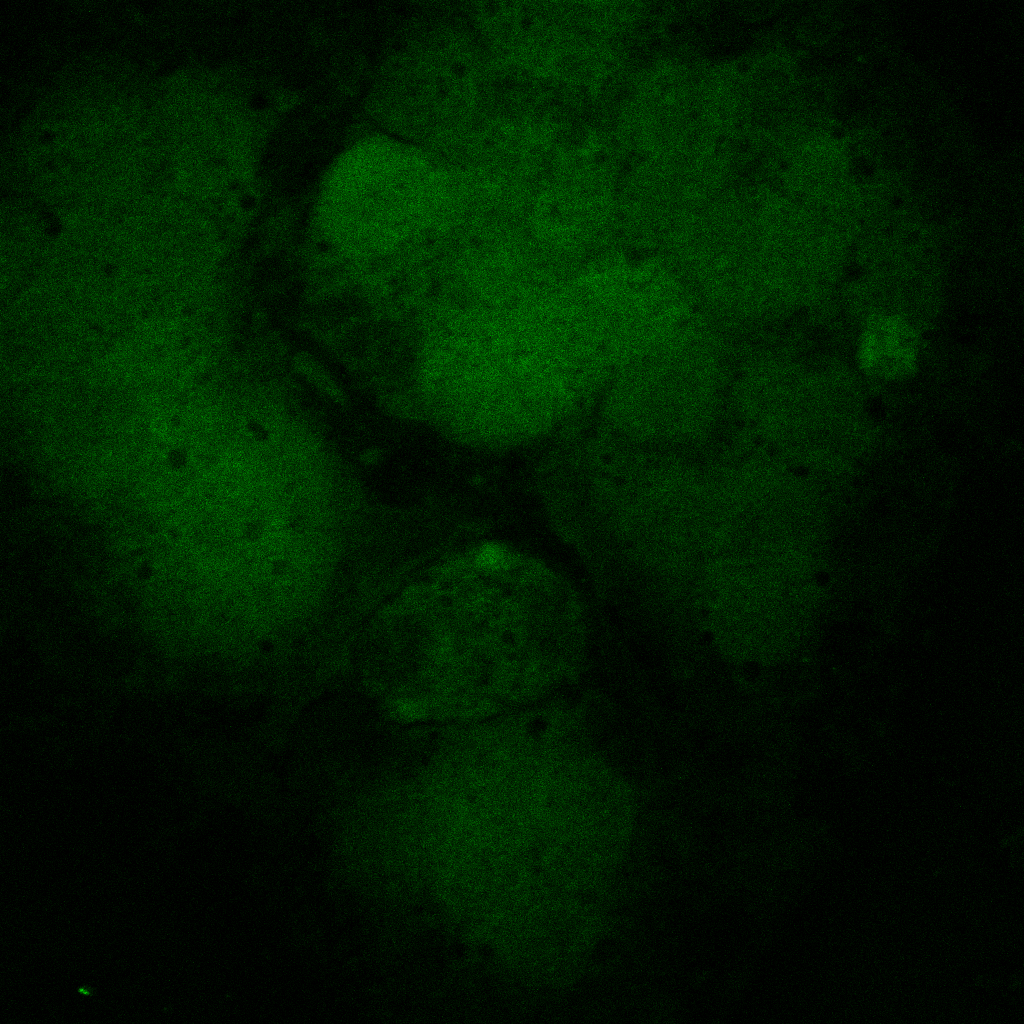

Supplement: Figure 3—figure supplement 3—source data 1. [file elife-85595-fig3-figsupp3-data1.zip › Figure 3-figure supplement 3-source data 1/Original_files/Figure 3-figure supplement 3B_S878A_O-GlcNAc_myc.tif]

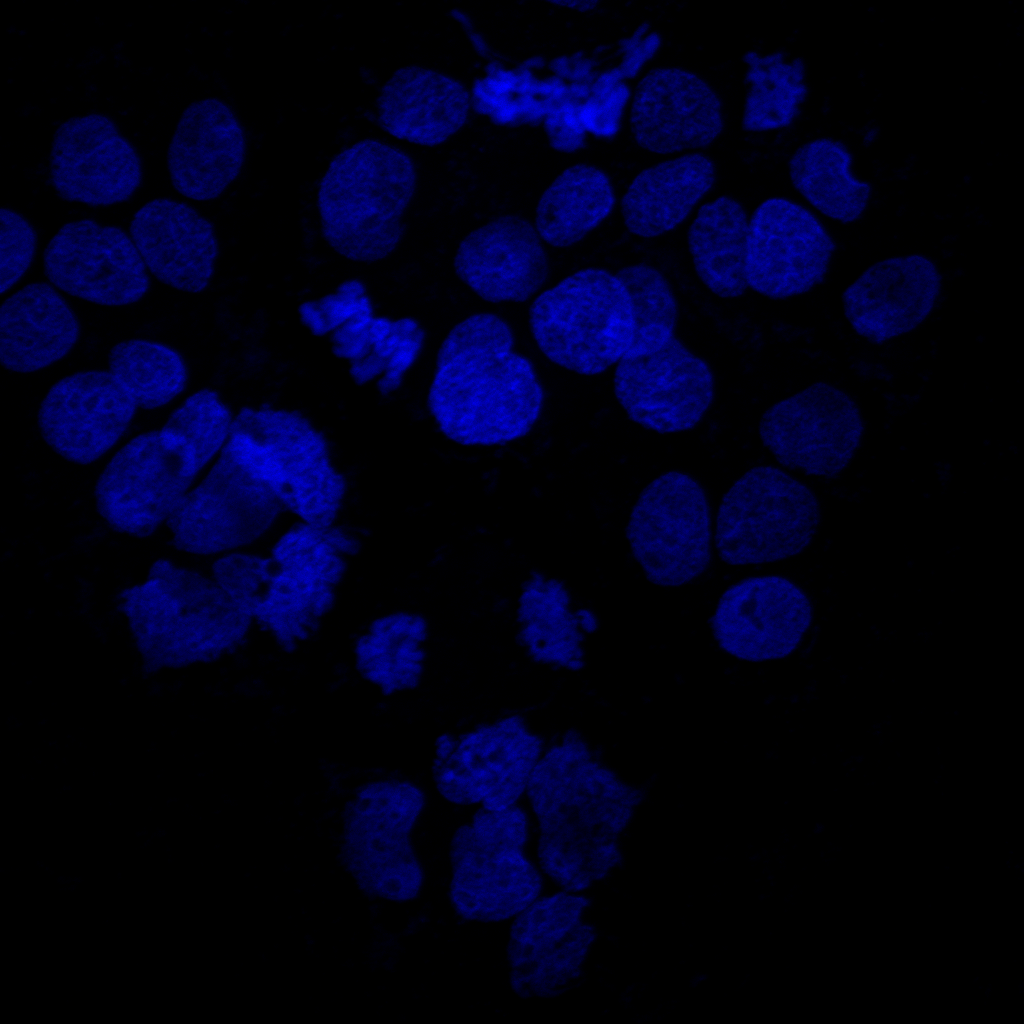

Supplement: Figure 3—figure supplement 3—source data 1. [file elife-85595-fig3-figsupp3-data1.zip › Figure 3-figure supplement 3-source data 1/Original_files/Figure 3-figure supplement 3B_S878A_O-GlcNAc_DAPI.tif]

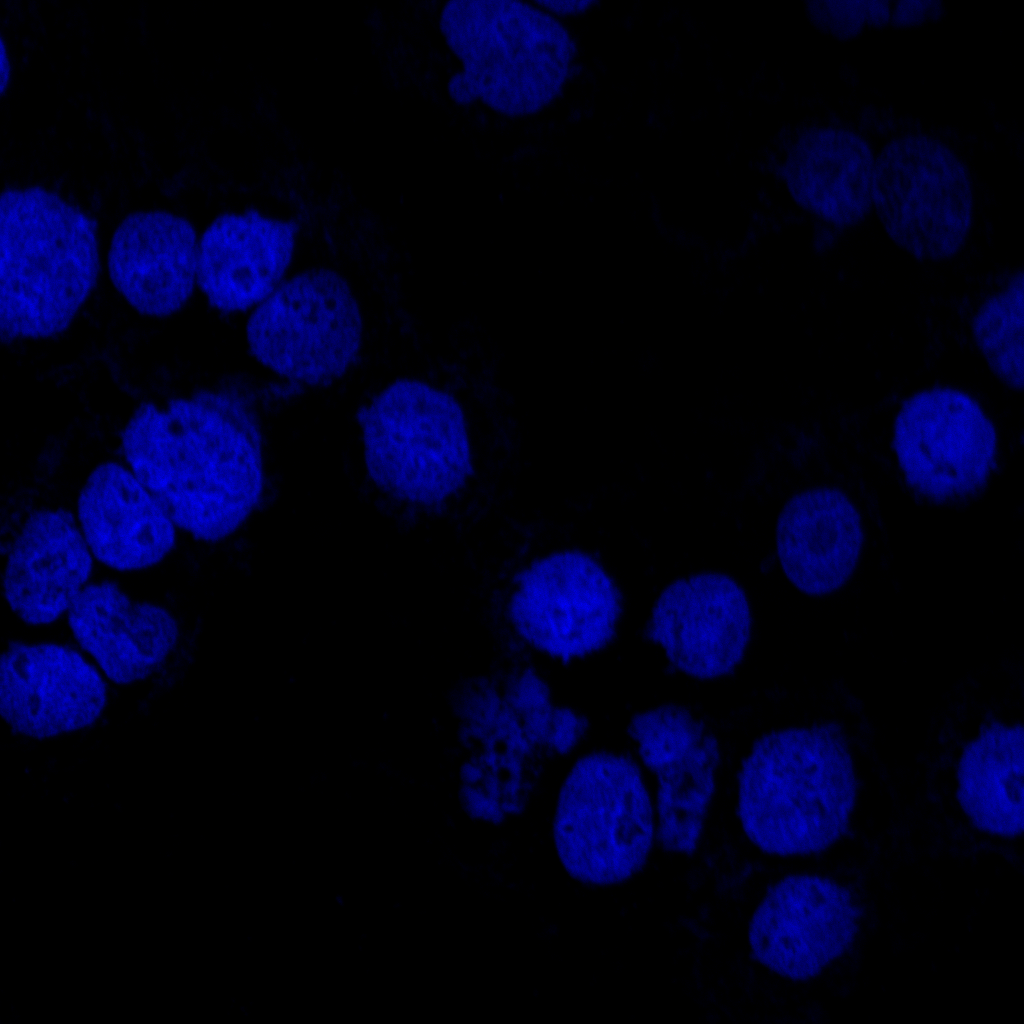

Supplement: Figure 3—figure supplement 3—source data 1. [file elife-85595-fig3-figsupp3-data1.zip › Figure 3-figure supplement 3-source data 1/Original_files/Figure 3-figure supplement 3B_S878A_CTRL_DAPI.tif]

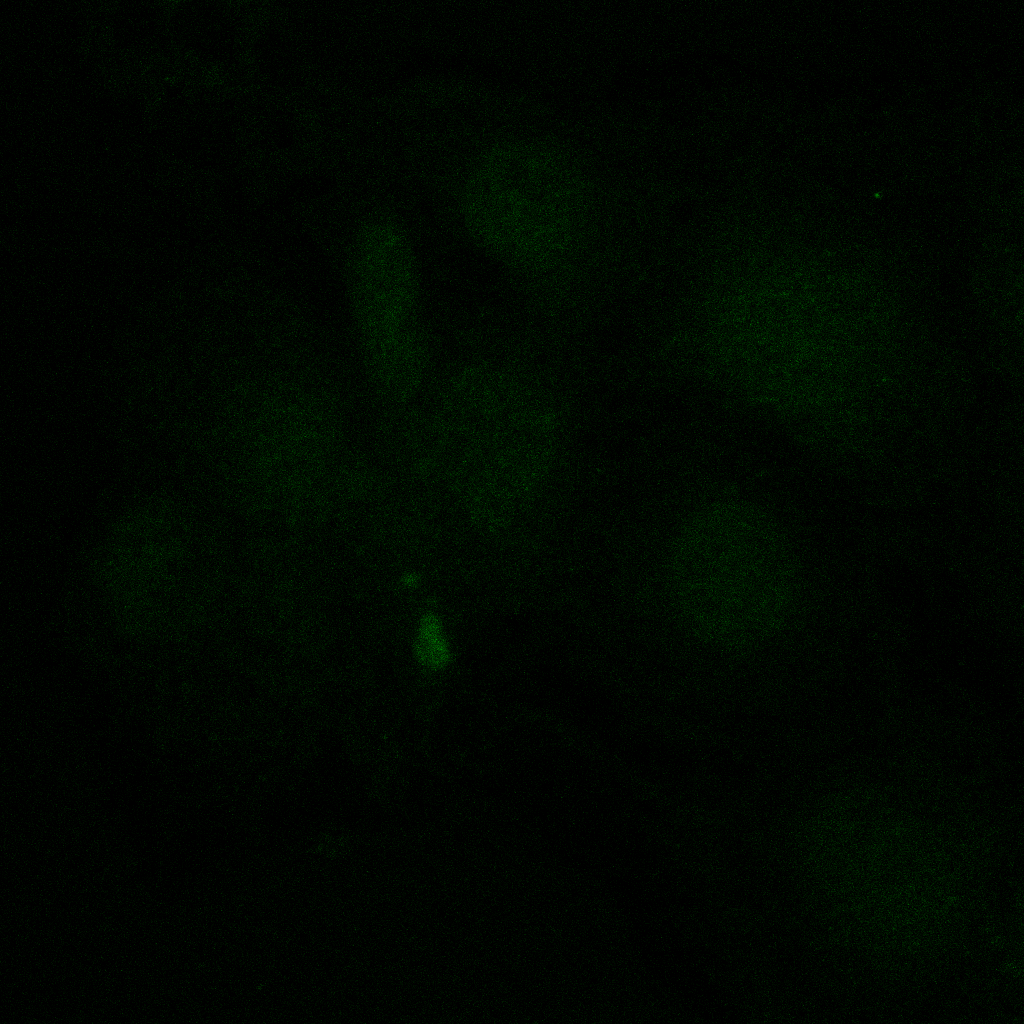

Supplement: Figure 3—figure supplement 3—source data 1. [file elife-85595-fig3-figsupp3-data1.zip › Figure 3-figure supplement 3-source data 1/Original_files/Figure 3-figure supplement 3A_WT_CTRL_myc.tif]

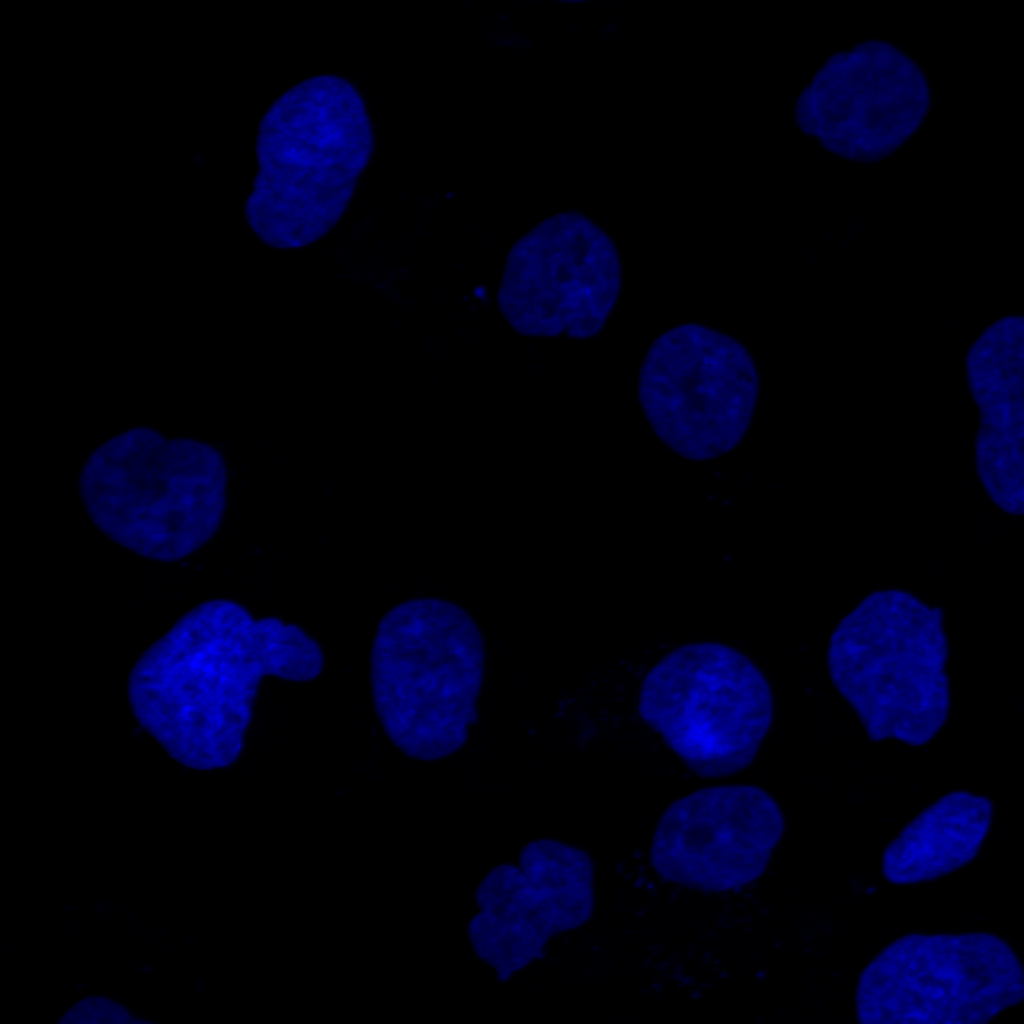

Supplement: Figure 3—figure supplement 3—source data 1. [file elife-85595-fig3-figsupp3-data1.zip › Figure 3-figure supplement 3-source data 1/Original_files/Figure 3-figure supplement 3A_S878A_O-GlcNAc_DAPI.tif]

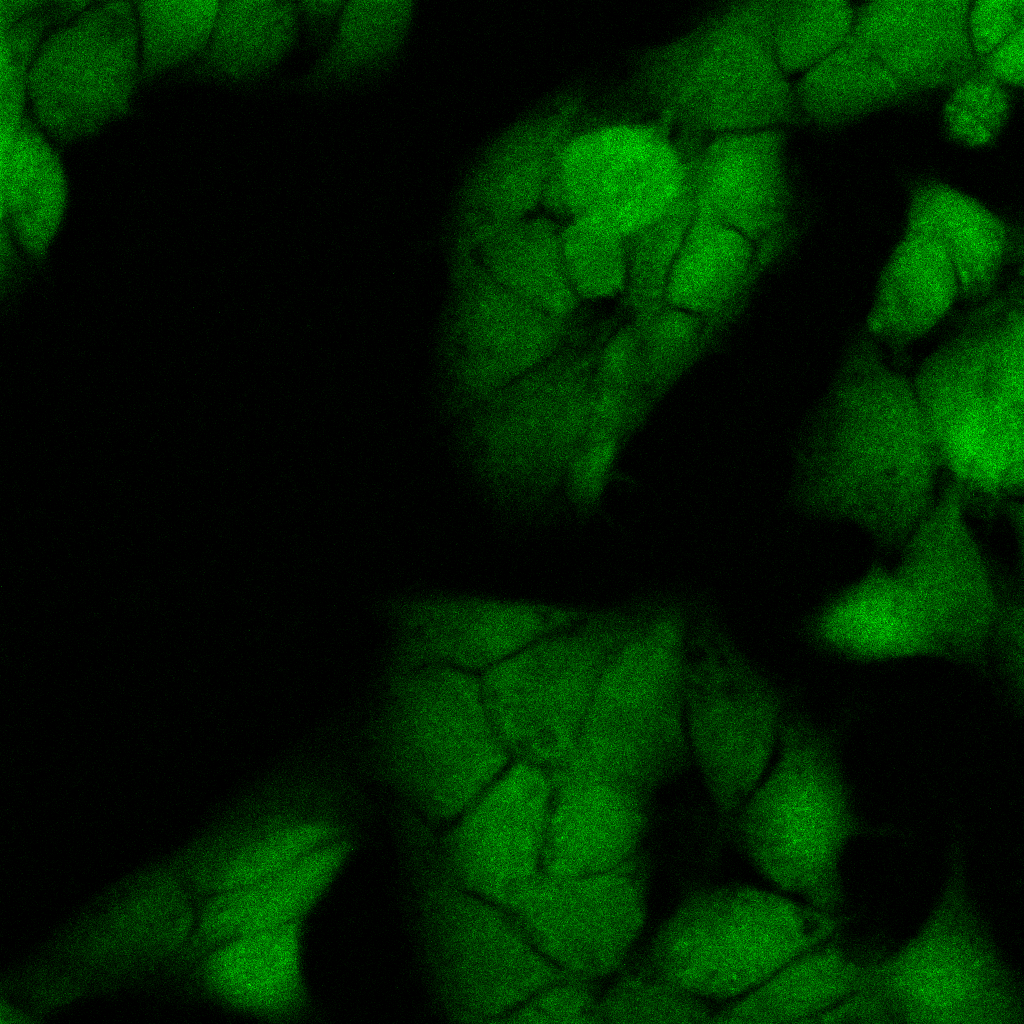

Supplement: Figure 3—figure supplement 3—source data 1. [file elife-85595-fig3-figsupp3-data1.zip › Figure 3-figure supplement 3-source data 1/Original_files/Figure 3-figure supplement 3B_WT_O-GlcNAc_myc.tif]

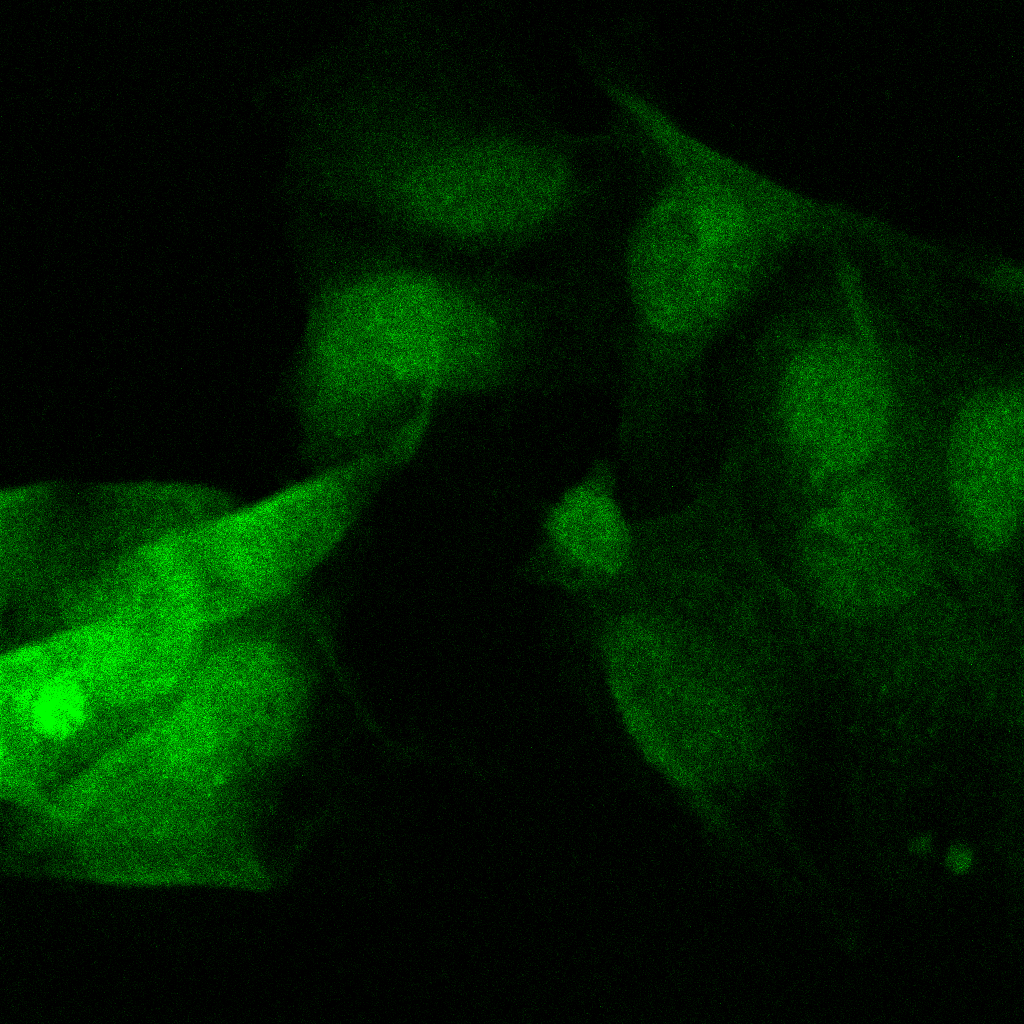

Supplement: Figure 3—figure supplement 3—source data 1. [file elife-85595-fig3-figsupp3-data1.zip › Figure 3-figure supplement 3-source data 1/Original_files/Figure 3-figure supplement 3A_WT_O-GlcNAc_myc.tif]

Figure 5A

DCFH-DA(ROS)

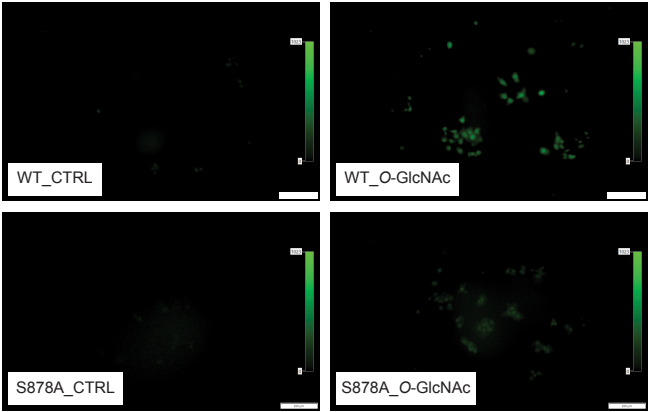

WT\_CTRL

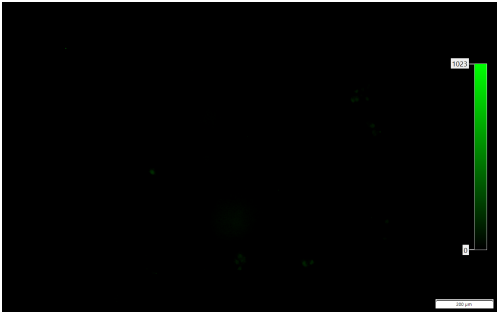

WT\_O-GlcNAc

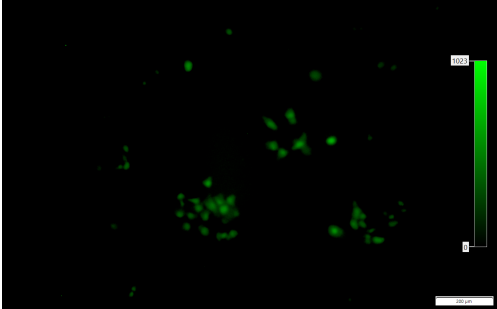

S878A\_CTRL

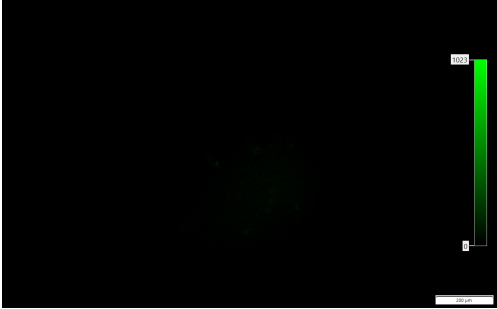

S878A\_O-GlcNAc

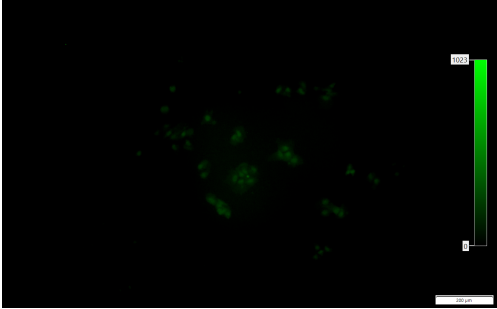

Supplement: Figure 5—source data 1. [file elife-85595-fig5-data1.zip › Figure 5-source data 1/Labeled_file/Figure 5A-source data 1.pdf]

Figure 5C

PI Staining (Cell Death)

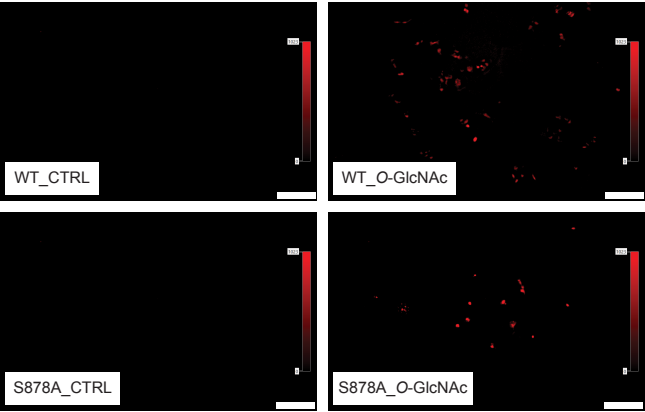

WT\_CTRL

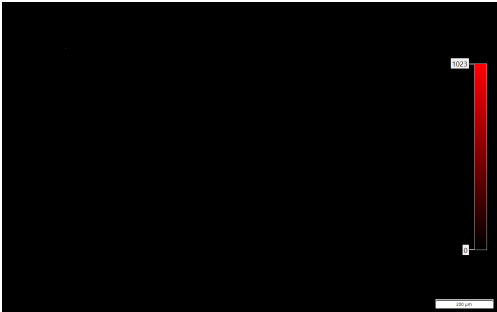

WT\_O-GlcNAc

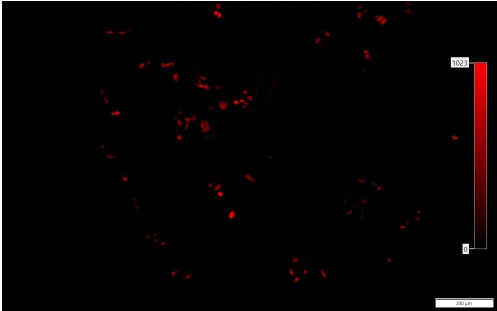

S878A\_CTRL

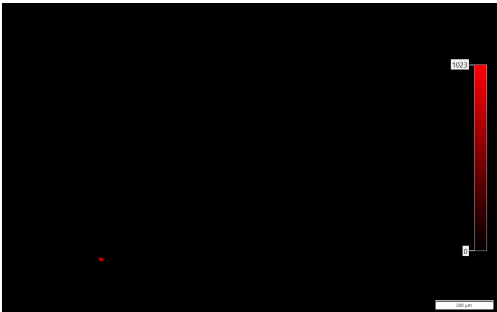

S878A\_O-GlcNAc

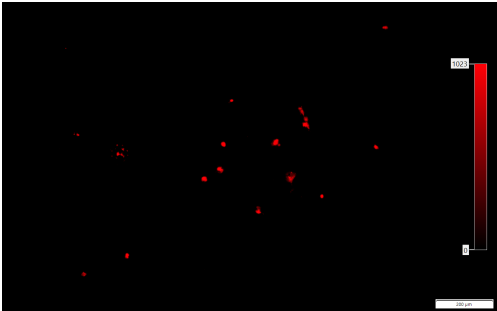

Supplement: Figure 5—source data 1. [file elife-85595-fig5-data1.zip › Figure 5-source data 1/Labeled_file/Figure 5C-source data 1.pdf]

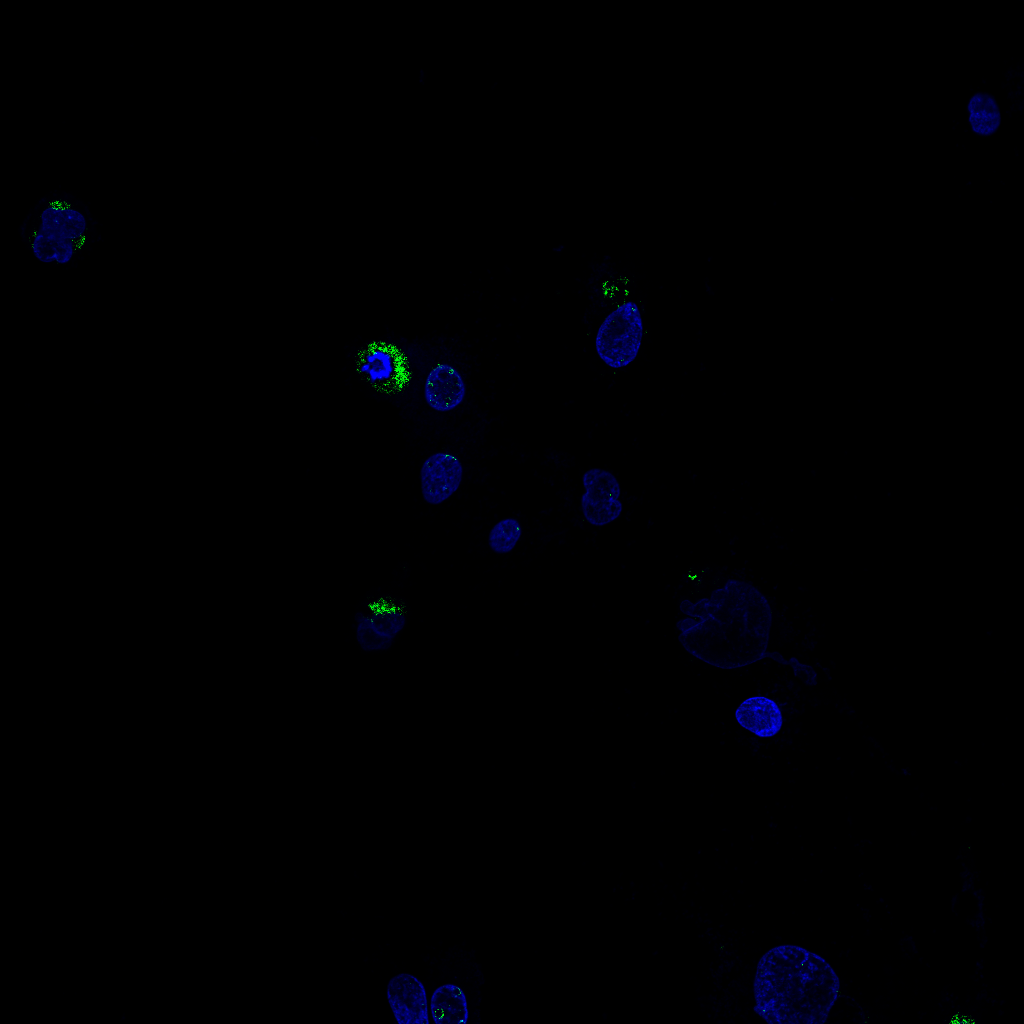

Supplement: Figure 5—source data 1. [file elife-85595-fig5-data1.zip › Figure 5-source data 1/Original_files/Figure 5B_S878A_O-GlcNAc_merge.tif]

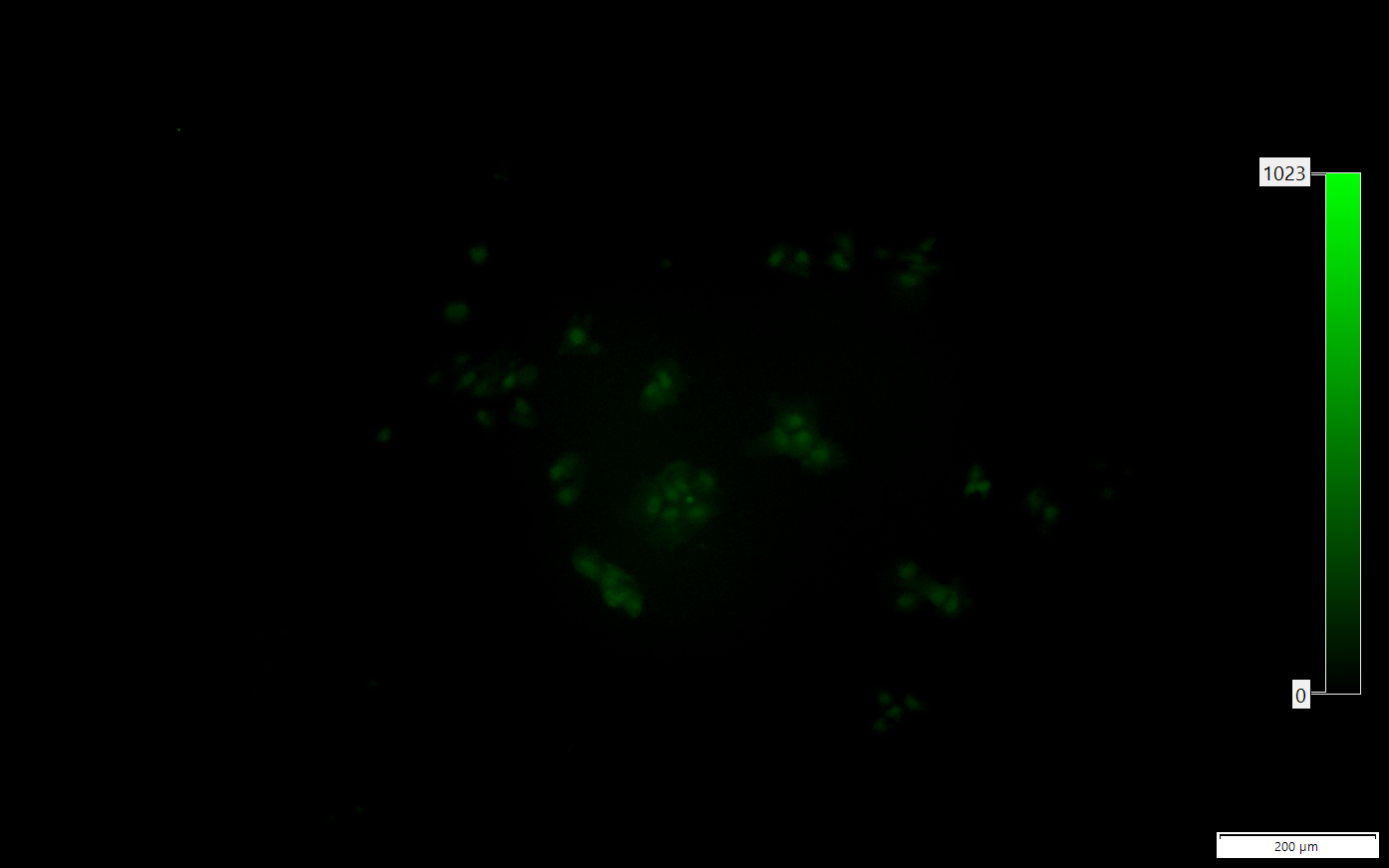

Supplement: Figure 5—source data 1. [file elife-85595-fig5-data1.zip › Figure 5-source data 1/Original_files/Figure 5A_S878A_O-GlcNAc.tif]

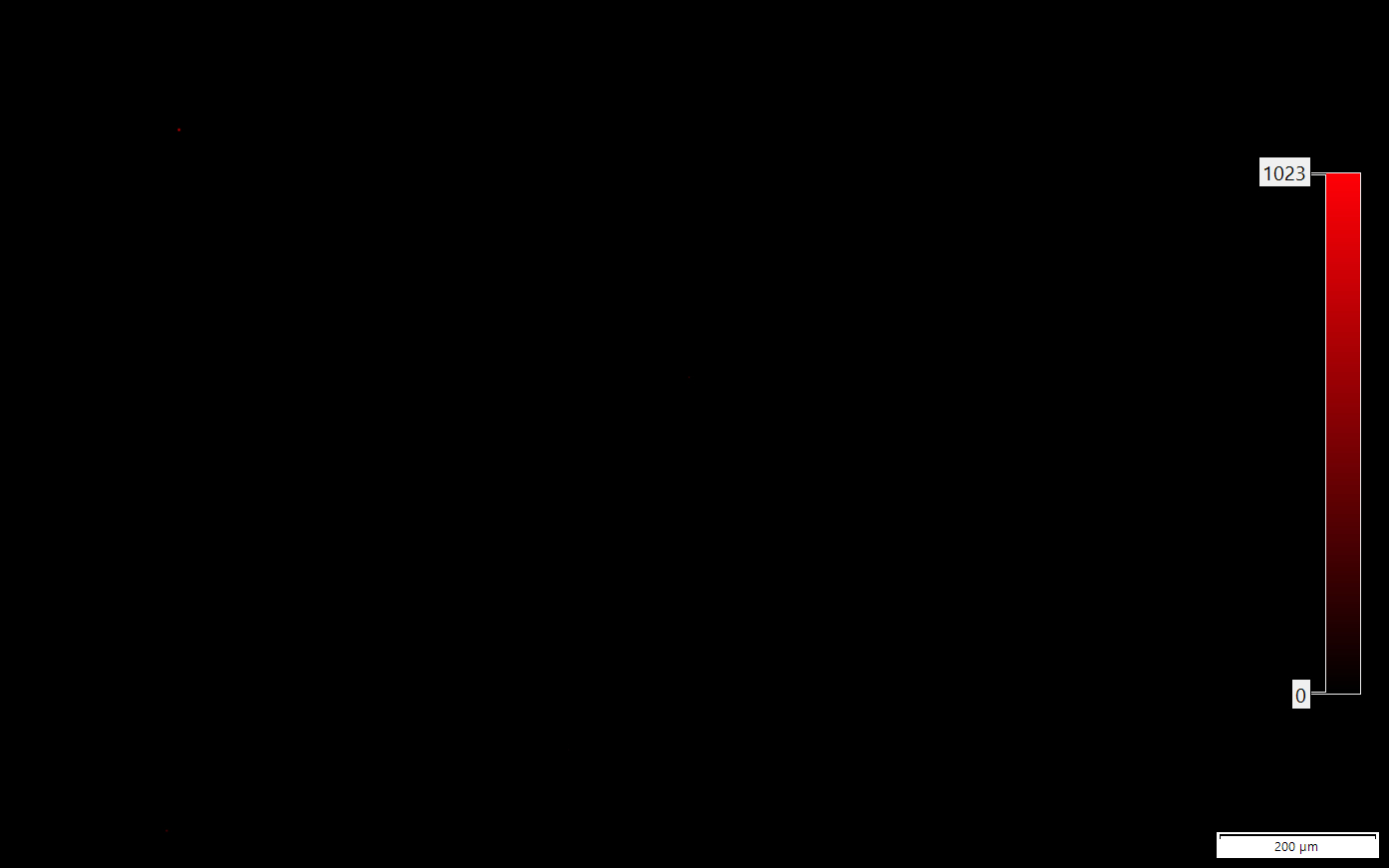

Supplement: Figure 5—source data 1. [file elife-85595-fig5-data1.zip › Figure 5-source data 1/Original_files/Figure 5C_WT_CTRL.tif]

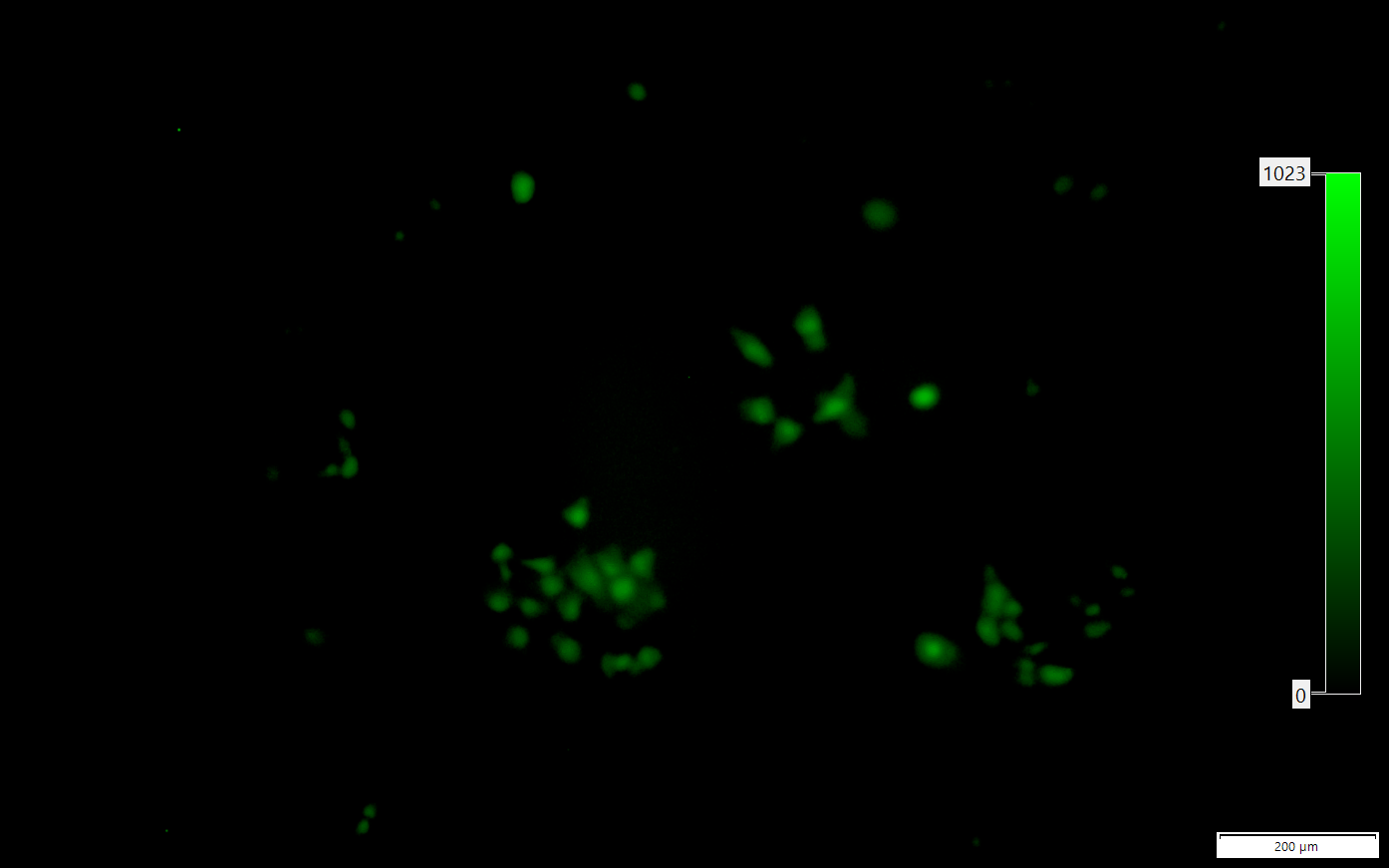

Supplement: Figure 5—source data 1. [file elife-85595-fig5-data1.zip › Figure 5-source data 1/Original_files/Figure 5A_WT_O-GlcNAc.tif]

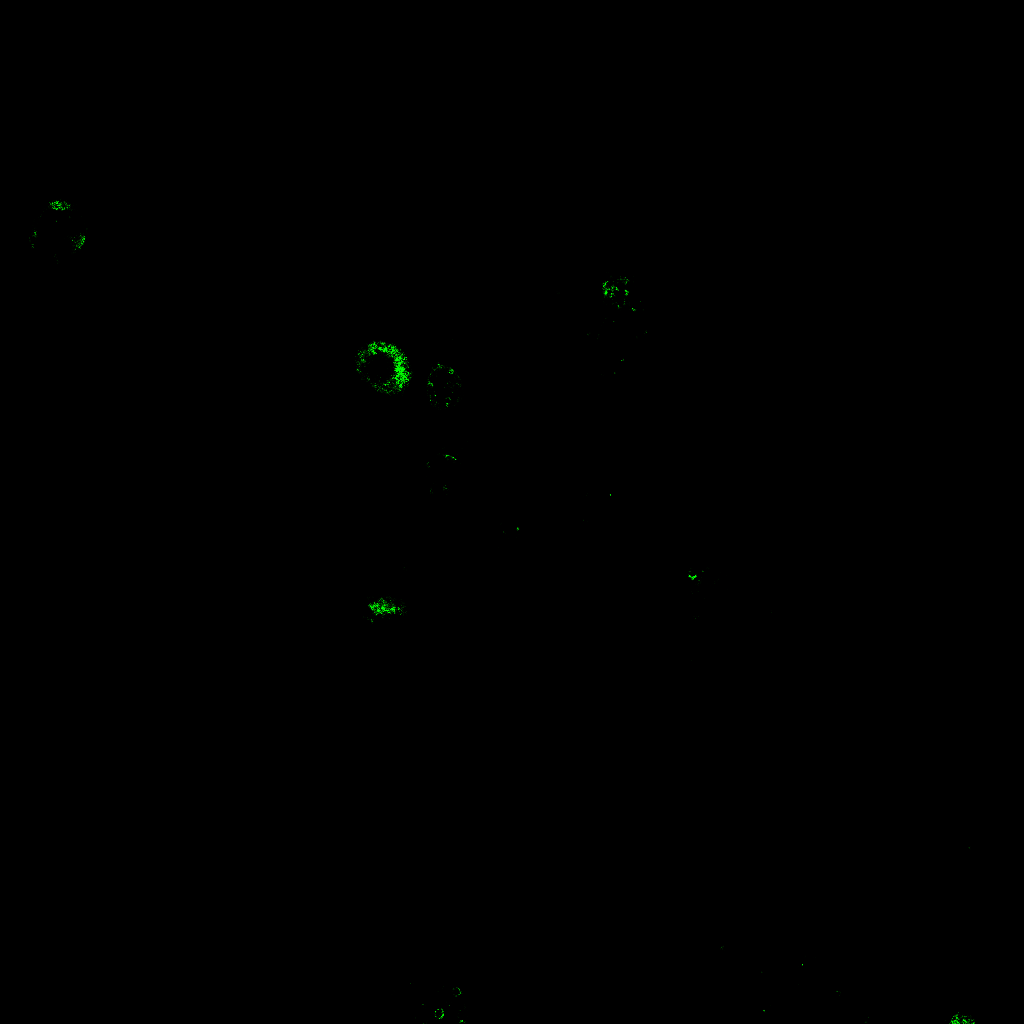

Supplement: Figure 5—source data 1. [file elife-85595-fig5-data1.zip › Figure 5-source data 1/Original_files/Figure 5B_S878A_O-GlcNAc_rH2AX.tif]

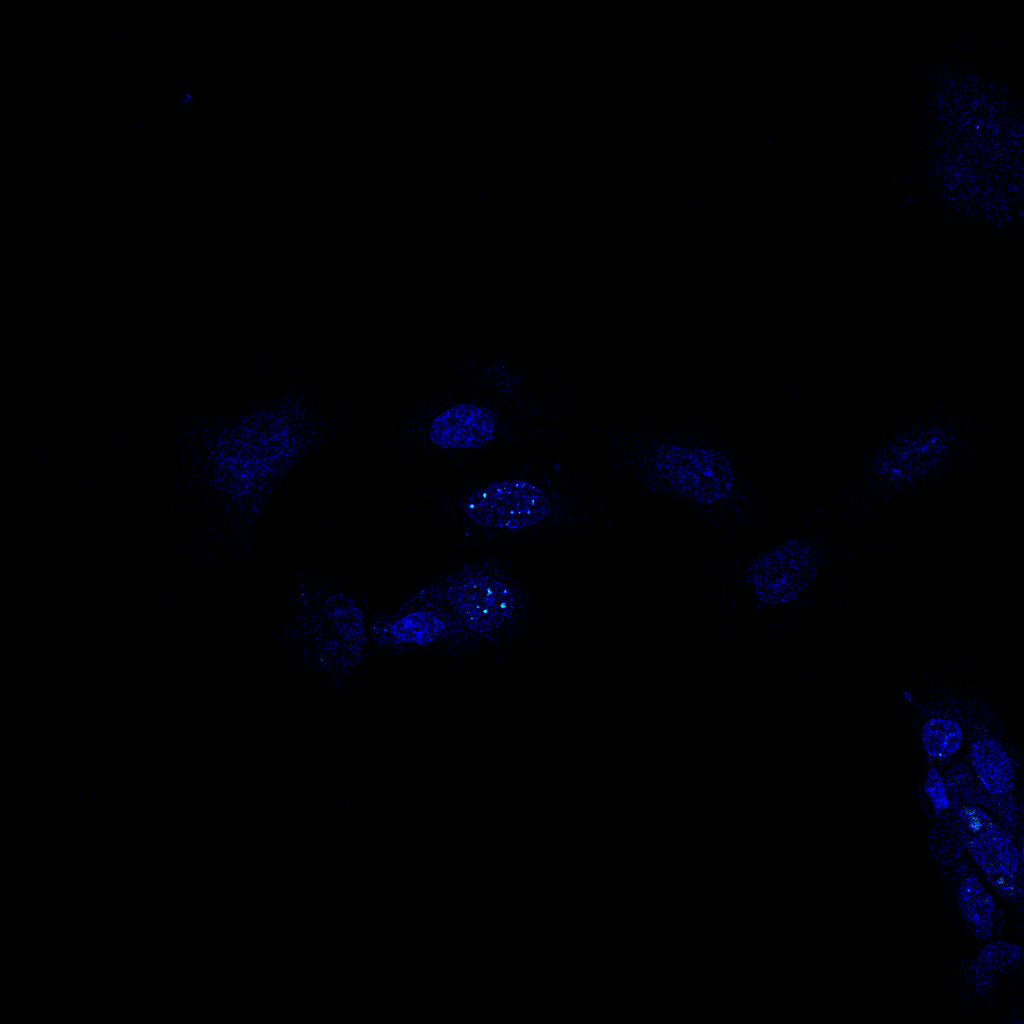

Supplement: Figure 5—source data 1. [file elife-85595-fig5-data1.zip › Figure 5-source data 1/Original_files/Figure 5B_S878A_CTRL_merge.tif]
