# Supplementary material for: Inhibition of DNMT1 methyltransferase activity via glucose-regulated O-GlcNAcylation alters the epigenome
Source: eLife. 2023 Jul 20;12:e85595. doi: 10.7554/eLife.85595 (PMC10390045; doi:10.7554/eLife.85595)

Figure 2—figure supplement 1

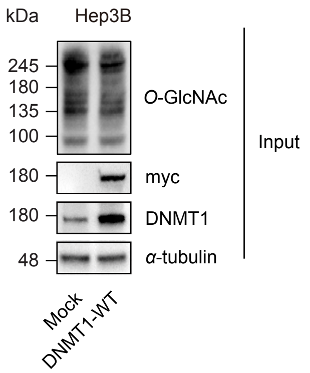

Input: *O*-GlcNAc

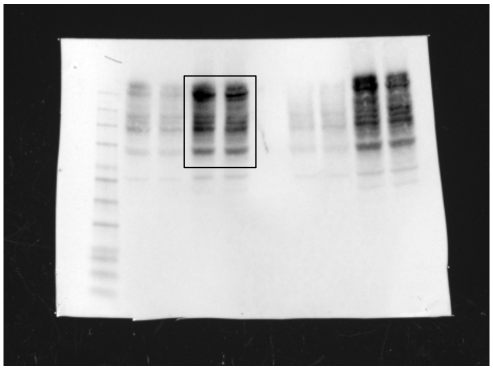

Input: myc

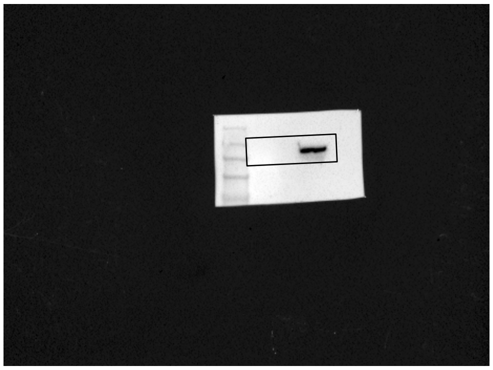

Input: Dnmt1

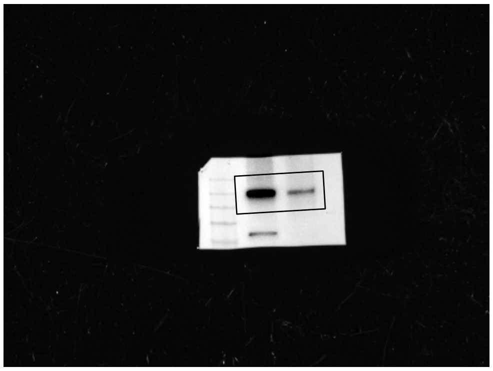

Input: *α*-Tubulin

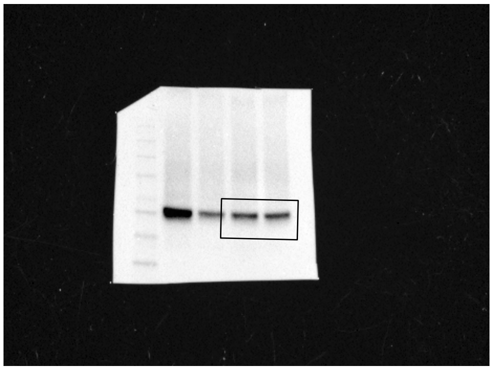

Figure 2—figure supplement 1

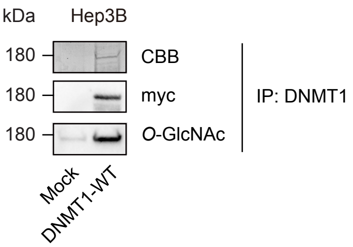

CBB

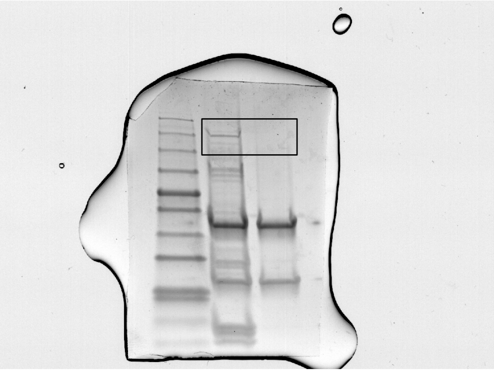

IP: myc

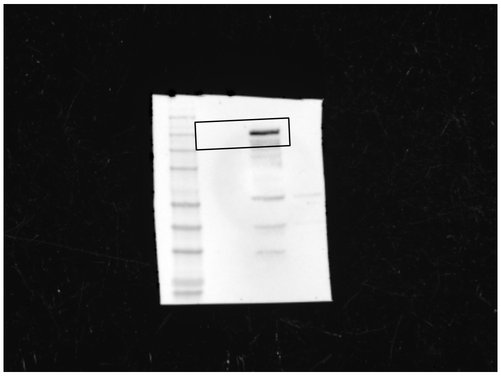

IP: *O*-GlcNAc

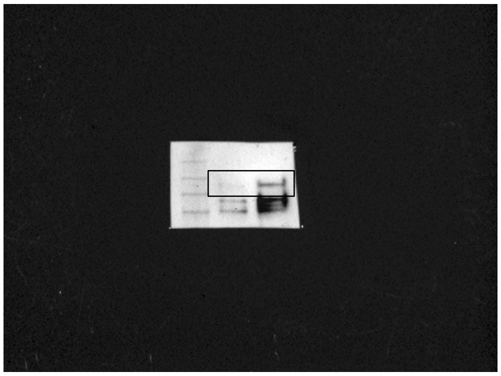

Supplement: Figure 2—figure supplement 1—source data 1. [file elife-85595-fig2-figsupp1-data1.zip › Figure 2-figure supplement 1-source data 1/Labeled_file/Figure 2-figure supplement 1-source data 1.pdf]
