# Supplementary material for: Inhibition of DNMT1 methyltransferase activity via glucose-regulated O-GlcNAcylation alters the epigenome
Source: eLife. 2023 Jul 20;12:e85595. doi: 10.7554/eLife.85595 (PMC10390045; doi:10.7554/eLife.85595)

Figure 5B_γH2A.X

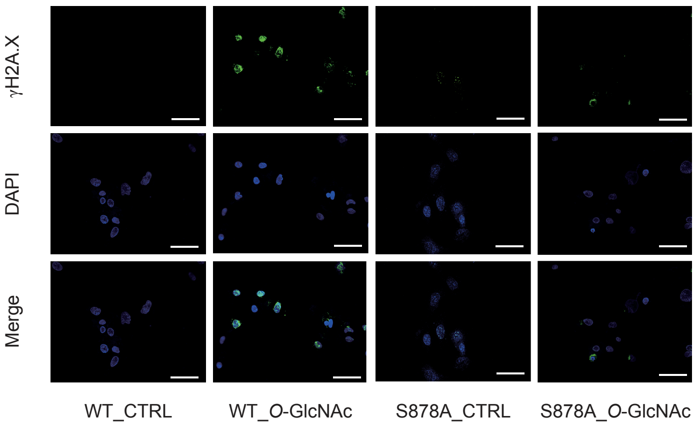

WT_CTRL

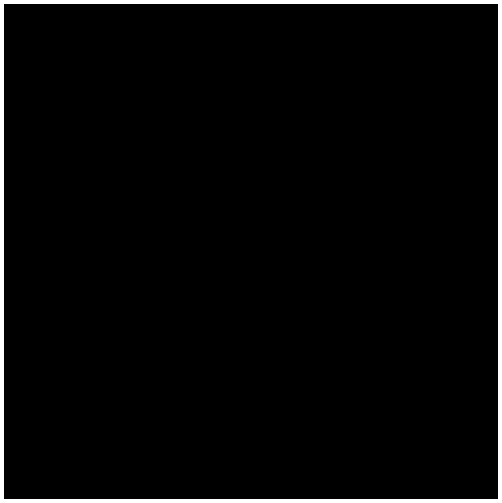

WT_*O*-GlcNAc

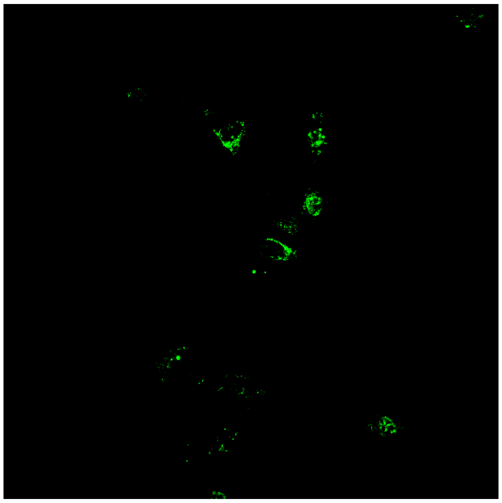

S878A_CTRL

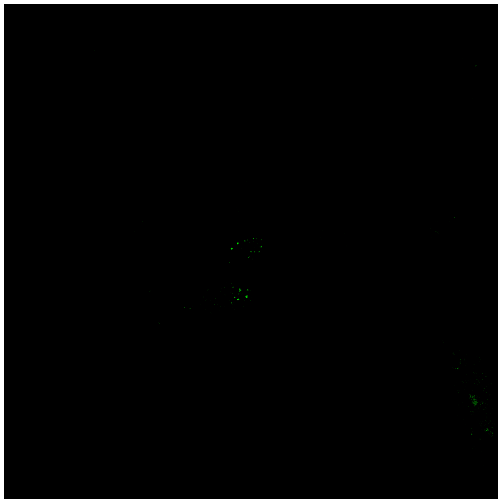

S878A_*O*-GlcNAc

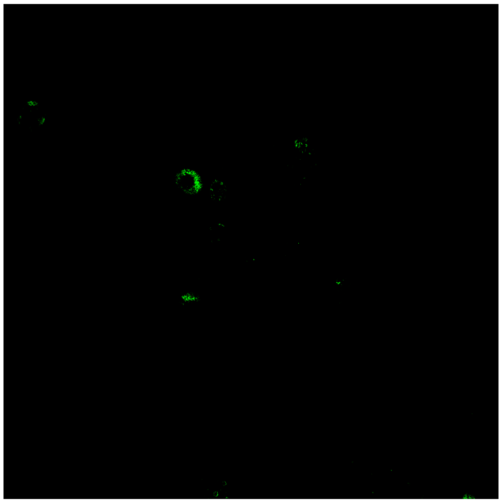

Figure 5B_DAPI

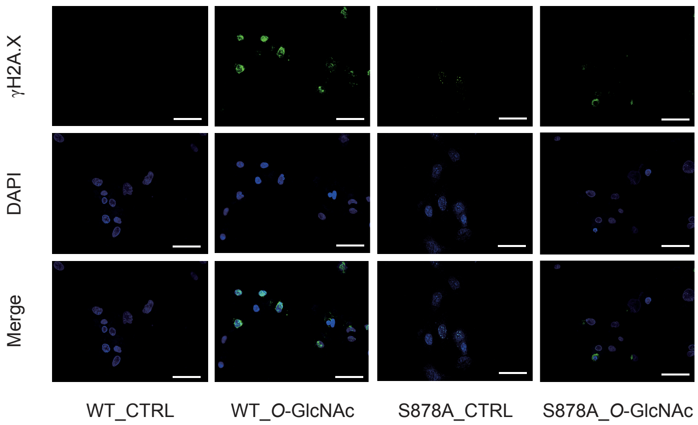

WT_CTRL

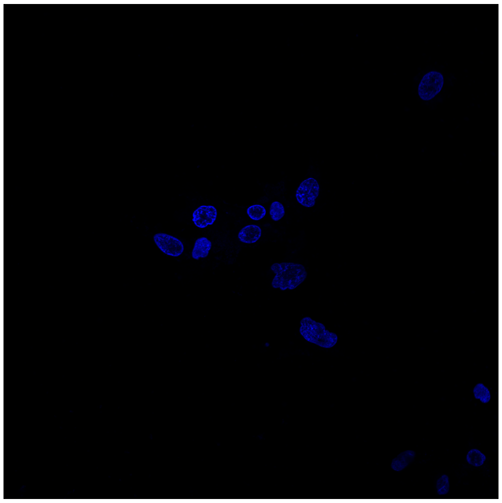

WT_*O*-GlcNAc

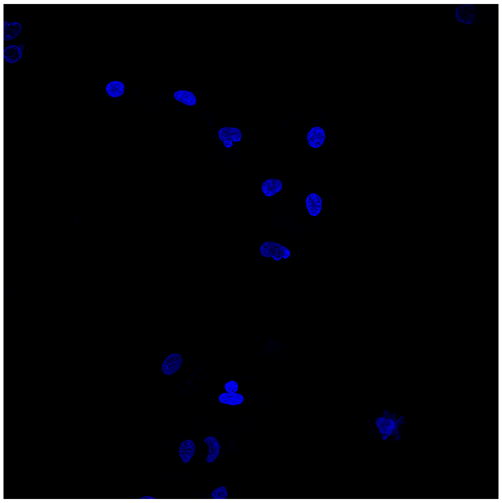

S878A_CTRL

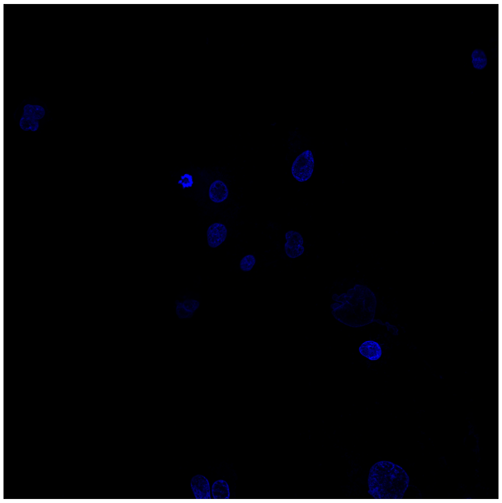

S878A_*O*-GlcNAc

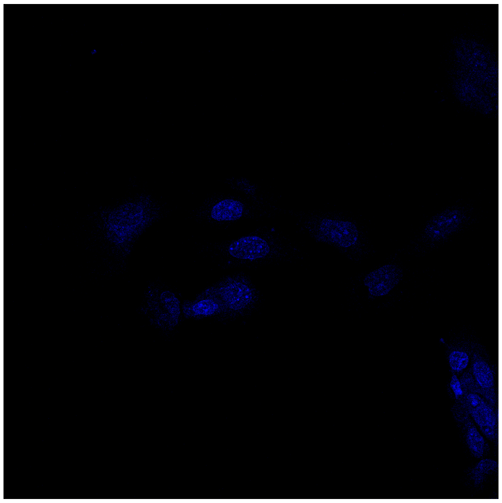

Figure 5B_merge

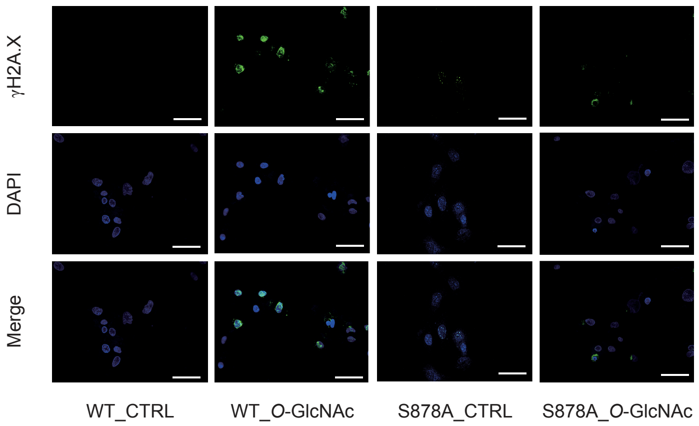

WT_CTRL

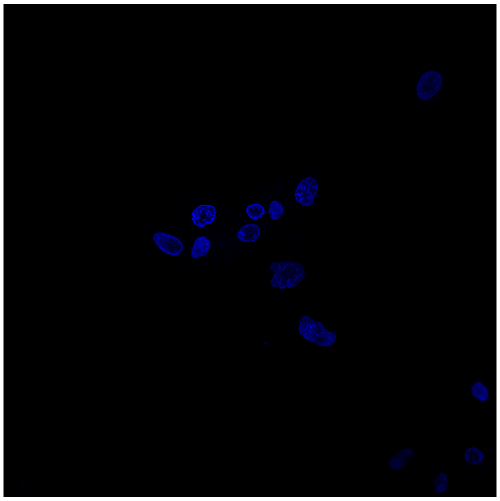

WT_*O*-GlcNAc

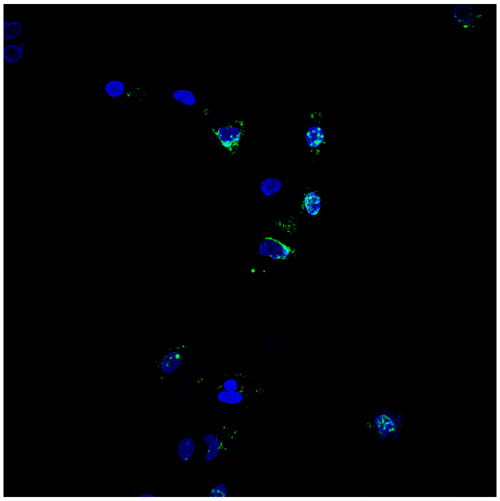

S878A_CTRL

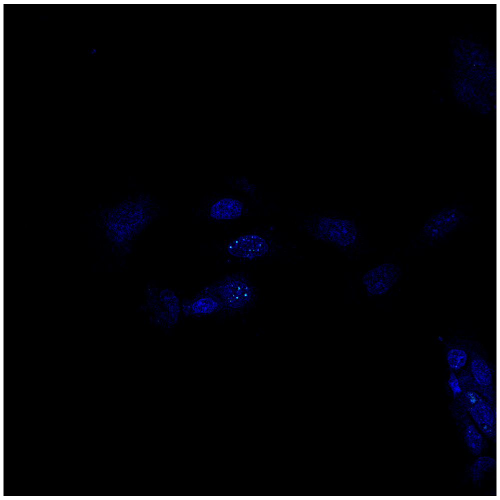

S878A_*O*-GlcNAc

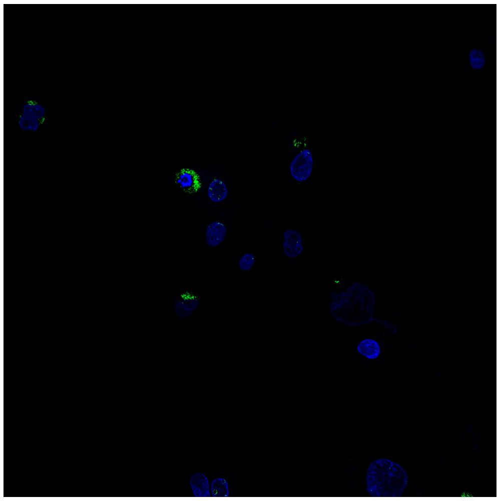

Supplement: Figure 5—source data 1. [file elife-85595-fig5-data1.zip › Figure 5-source data 1/Labeled_file/Figure 5B-source data 1.pdf]
