# Supplementary figures and images for: Inhibition of DNMT1 methyltransferase activity via glucose-regulated O-GlcNAcylation alters the epigenome (part 2 of 2)
Source: eLife. 2023 Jul 20;12:e85595. doi: 10.7554/eLife.85595 (PMC10390045; doi:10.7554/eLife.85595)

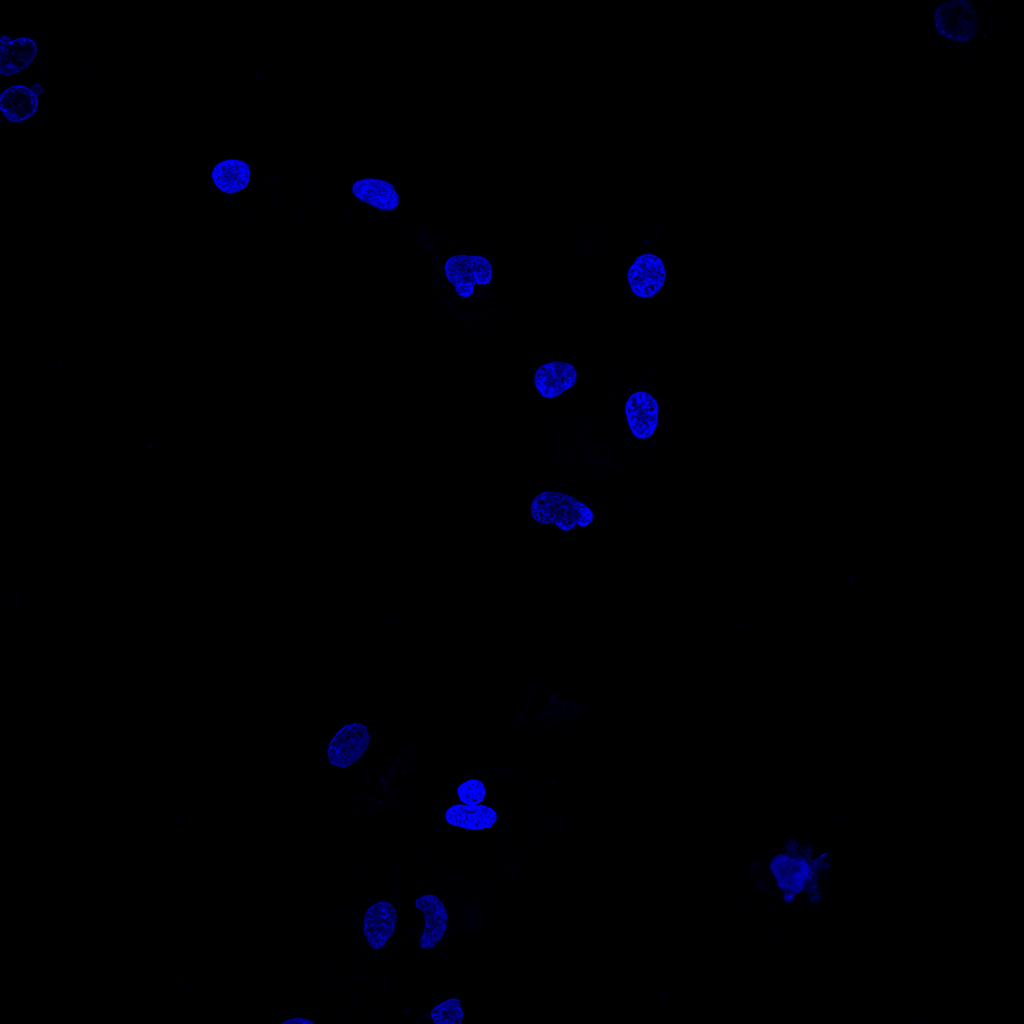

Supplement: Figure 5—source data 1. [file elife-85595-fig5-data1.zip › Figure 5-source data 1/Original_files/Figure 5B_WT_O-GlcNAc_DAPI.tif]

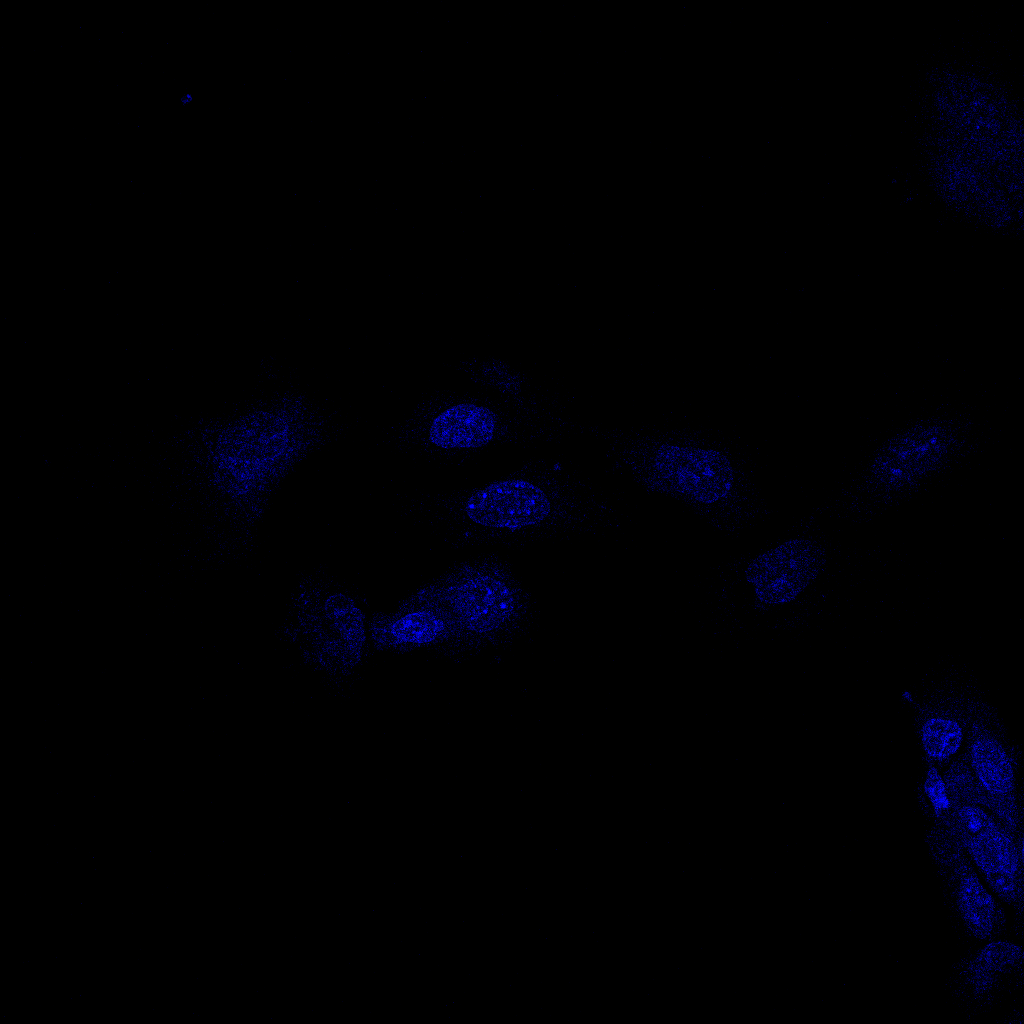

Supplement: Figure 5—source data 1. [file elife-85595-fig5-data1.zip › Figure 5-source data 1/Original_files/Figure 5B_S878A_CTRL_DAPI.tif]

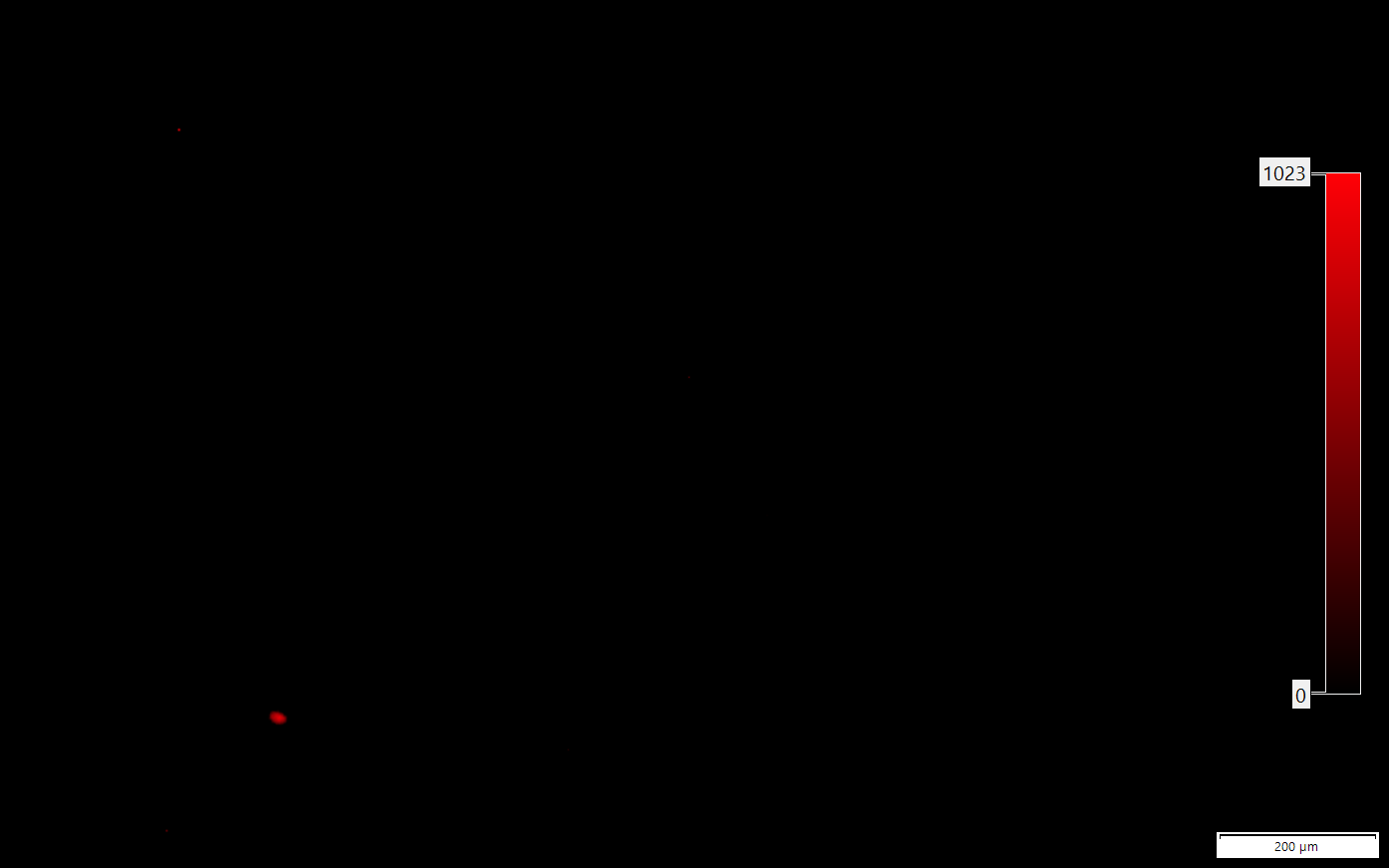

Supplement: Figure 5—source data 1. [file elife-85595-fig5-data1.zip › Figure 5-source data 1/Original_files/Figure 5C_S878A_CTRL.tif]

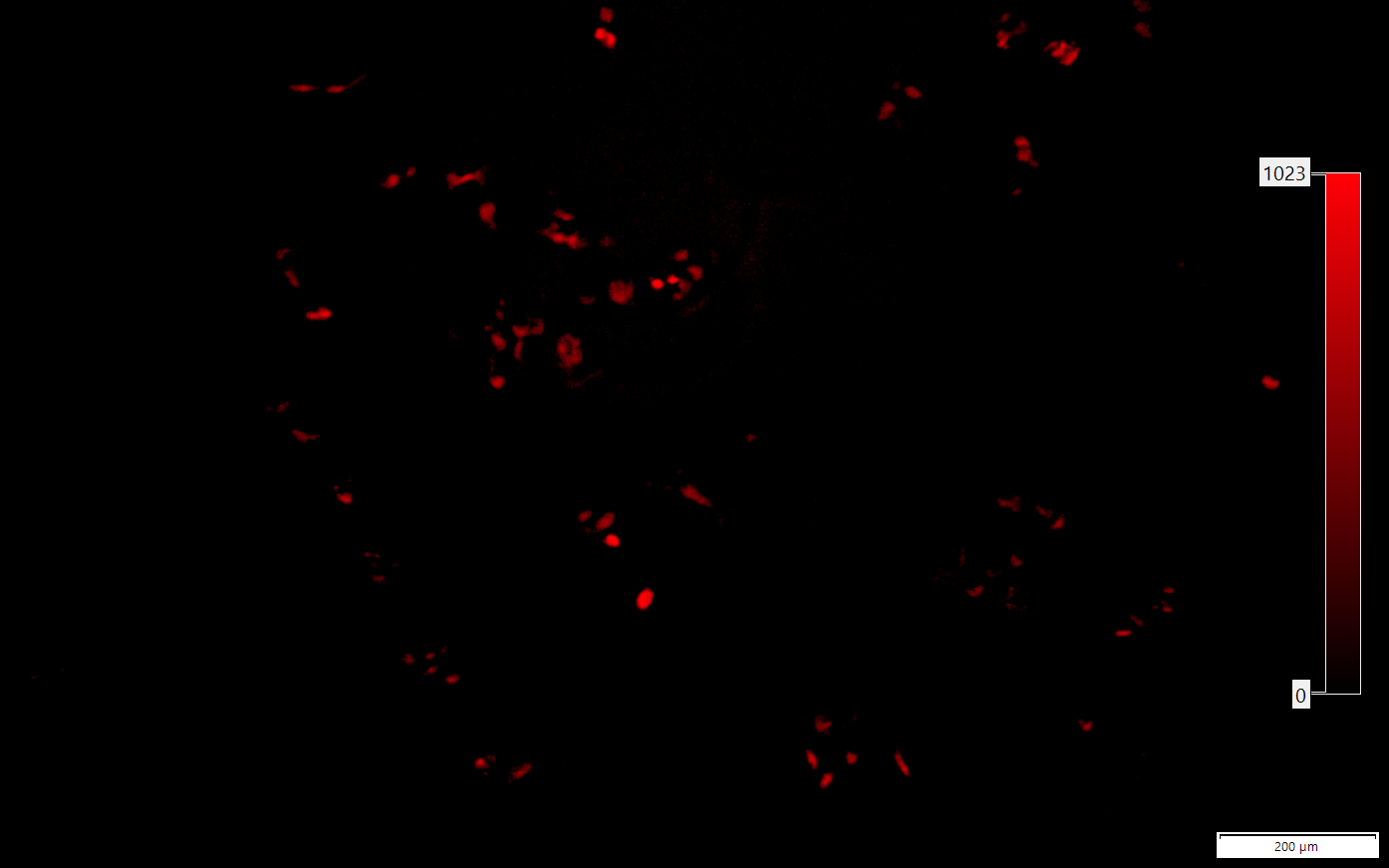

Supplement: Figure 5—source data 1. [file elife-85595-fig5-data1.zip › Figure 5-source data 1/Original_files/Figure 5C_WT_O-GlcNAc.tif]

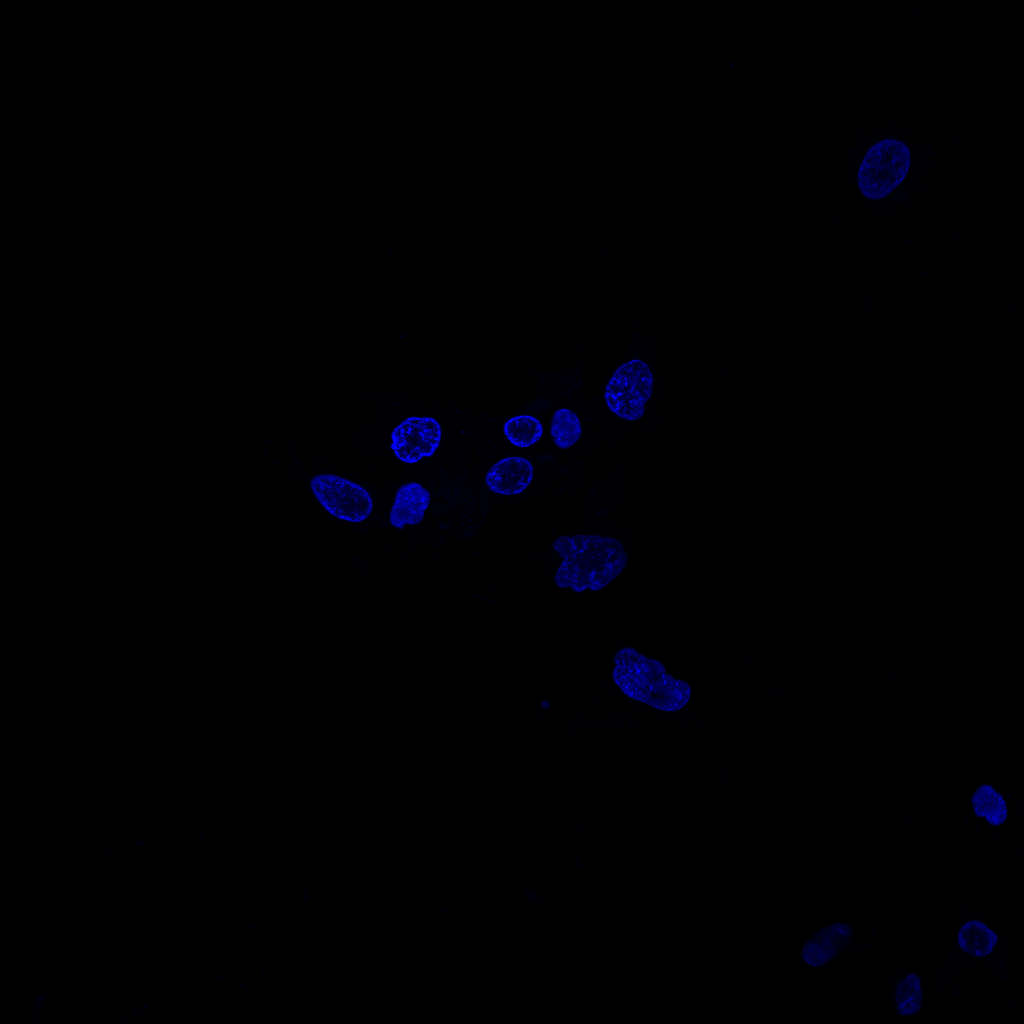

Supplement: Figure 5—source data 1. [file elife-85595-fig5-data1.zip › Figure 5-source data 1/Original_files/Figure 5B_WT_CTRL_merge.tif]

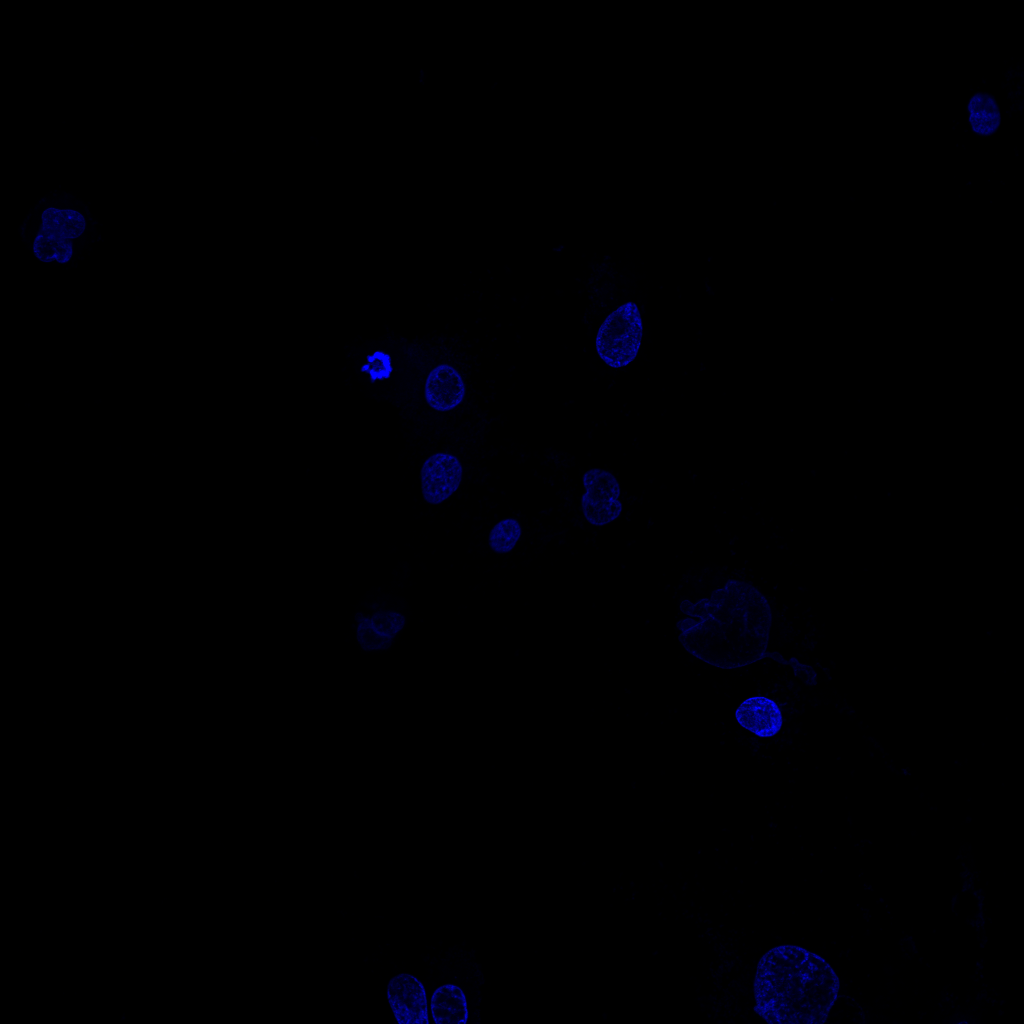

Supplement: Figure 5—source data 1. [file elife-85595-fig5-data1.zip › Figure 5-source data 1/Original_files/Figure 5B_S878A_O-GlcNAc_DAPI.tif]

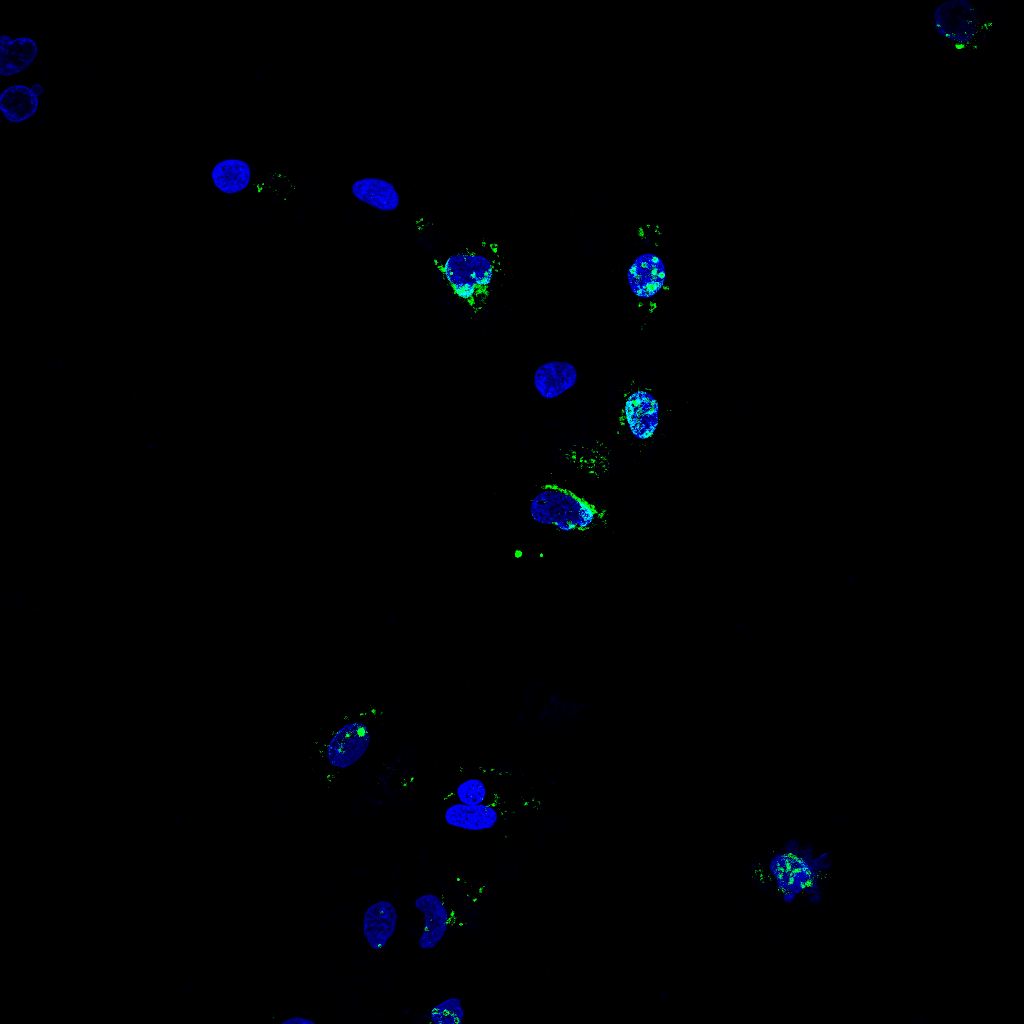

Supplement: Figure 5—source data 1. [file elife-85595-fig5-data1.zip › Figure 5-source data 1/Original_files/Figure 5B_WT_O-GlcNAc_merge.tif]

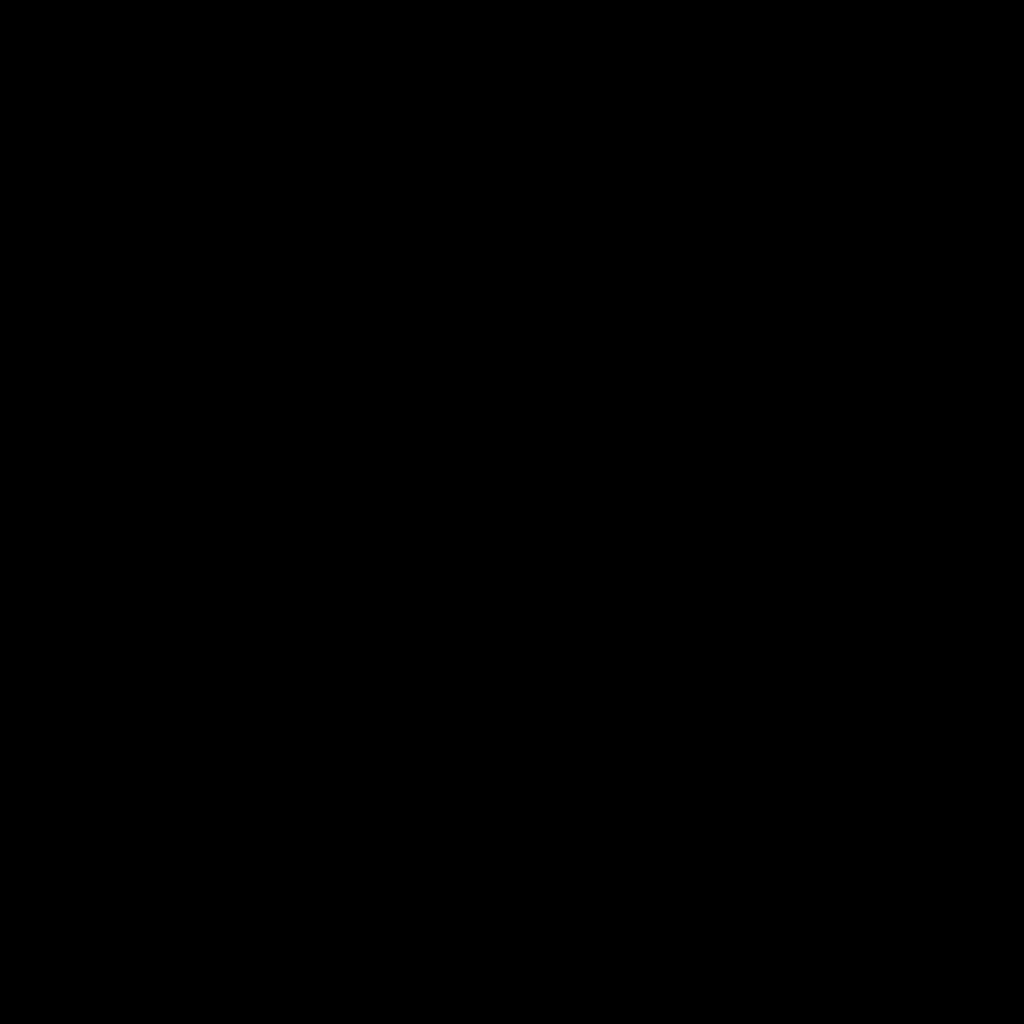

Supplement: Figure 5—source data 1. [file elife-85595-fig5-data1.zip › Figure 5-source data 1/Original_files/Figure 5B_WT_CTRL_rH2AX.tif]

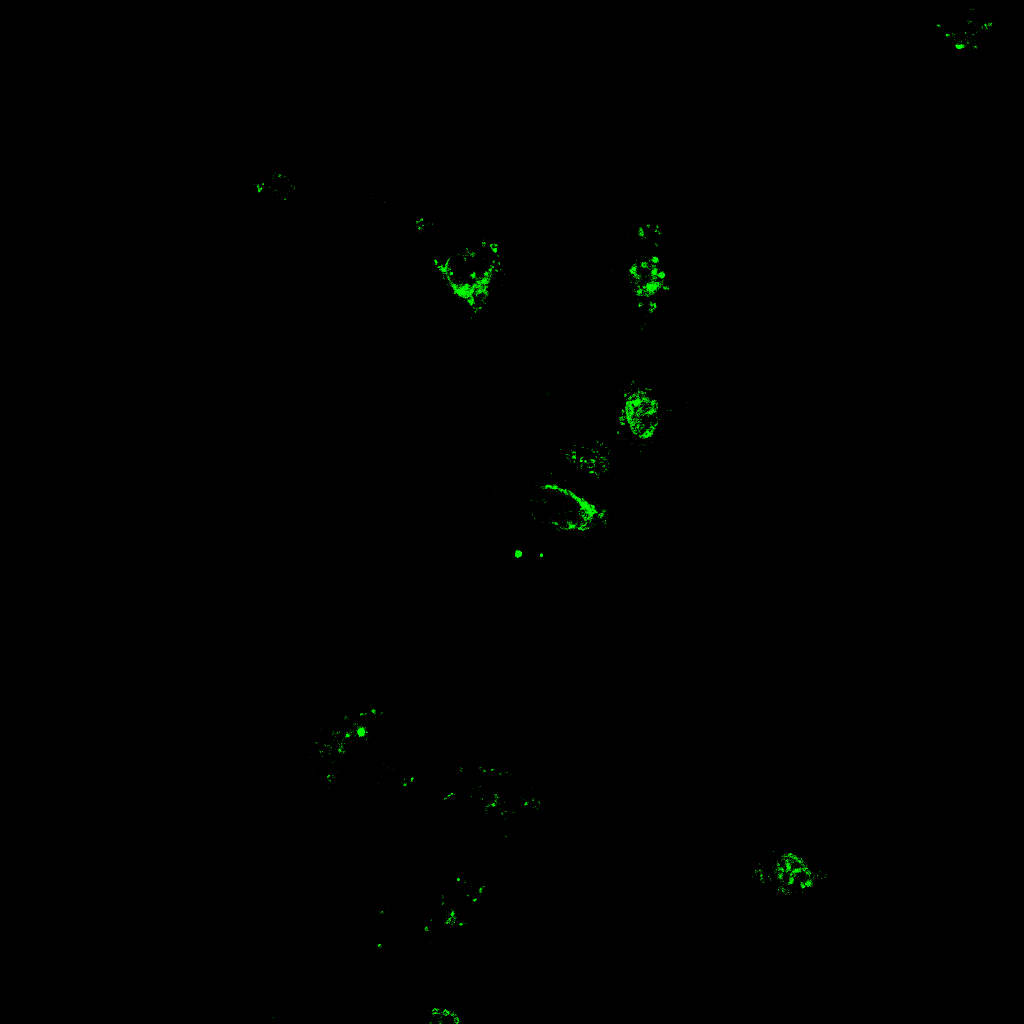

Supplement: Figure 5—source data 1. [file elife-85595-fig5-data1.zip › Figure 5-source data 1/Original_files/Figure 5B_WT_O-GlcNAc_rH2AX.tif]

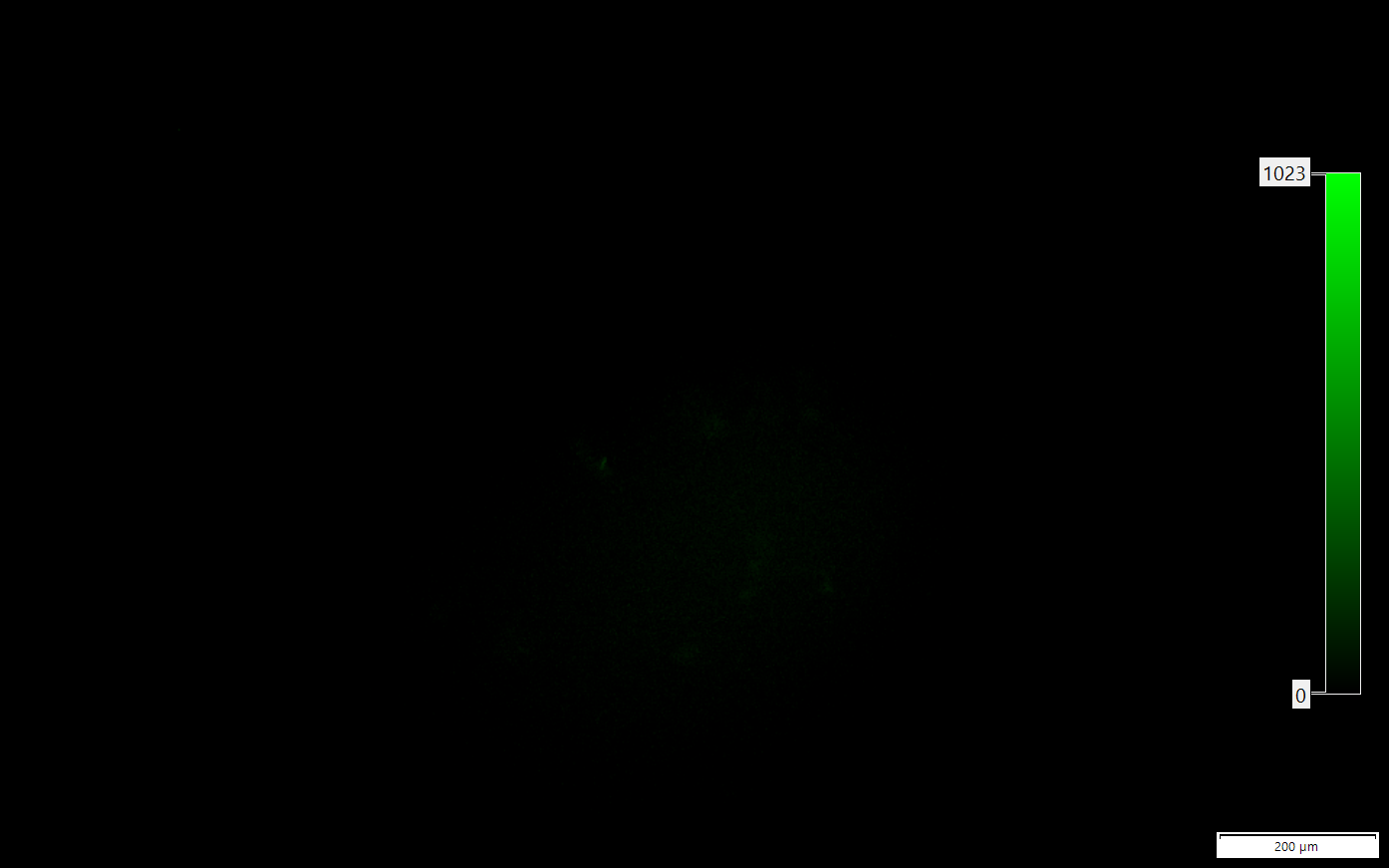

Supplement: Figure 5—source data 1. [file elife-85595-fig5-data1.zip › Figure 5-source data 1/Original_files/Figure 5A_S878A_CTRL.tif]

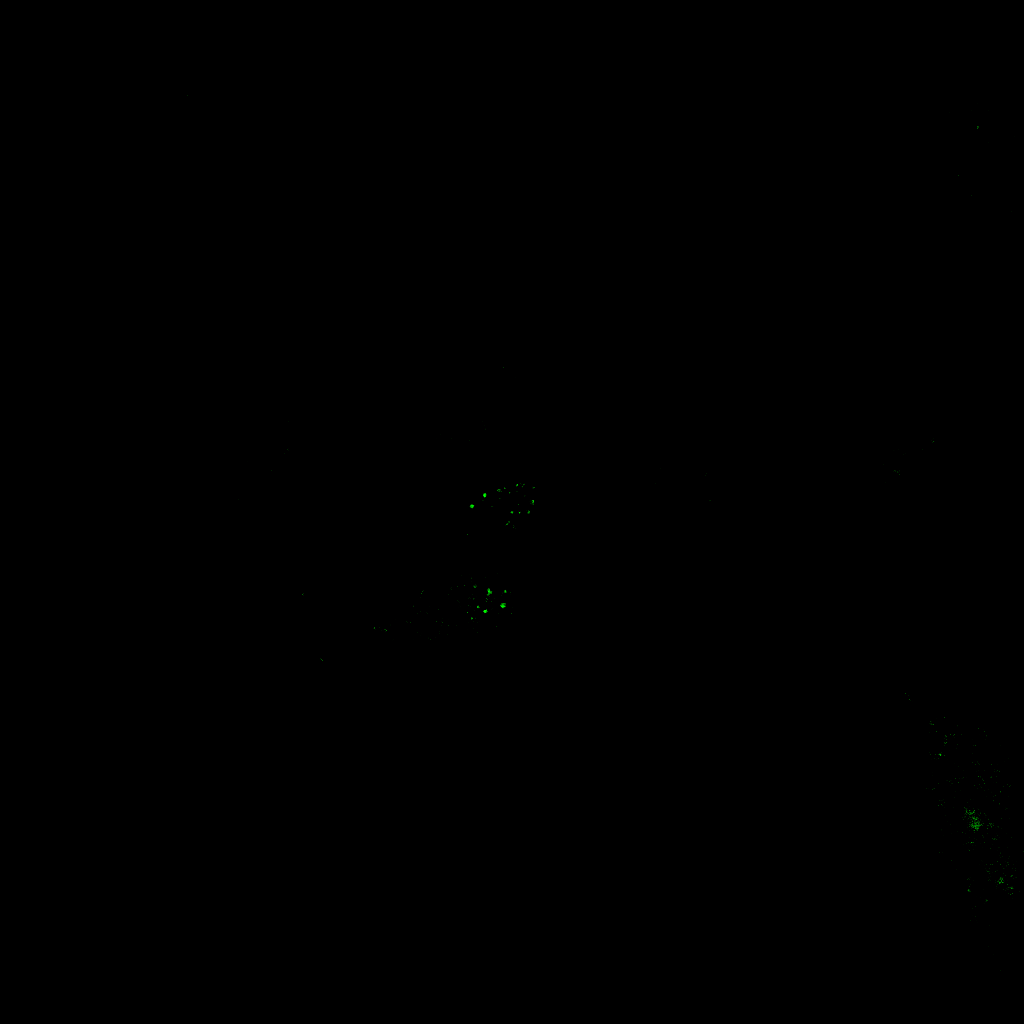

Supplement: Figure 5—source data 1. [file elife-85595-fig5-data1.zip › Figure 5-source data 1/Original_files/Figure 5B_S878A_CTRL_rH2AX.tif]

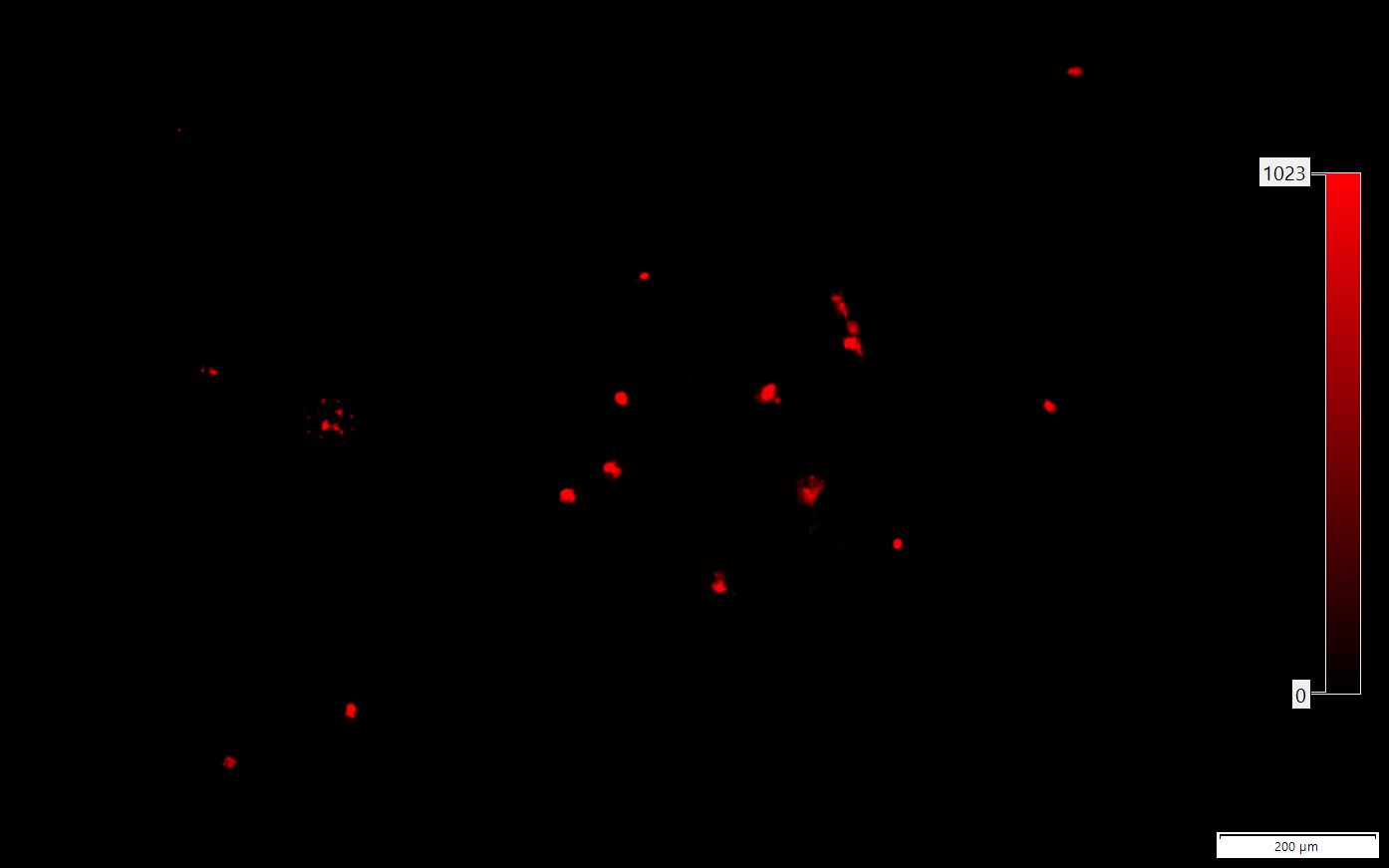

Supplement: Figure 5—source data 1. [file elife-85595-fig5-data1.zip › Figure 5-source data 1/Original_files/Figure 5C_S878A_O-GlcNAc.tif]

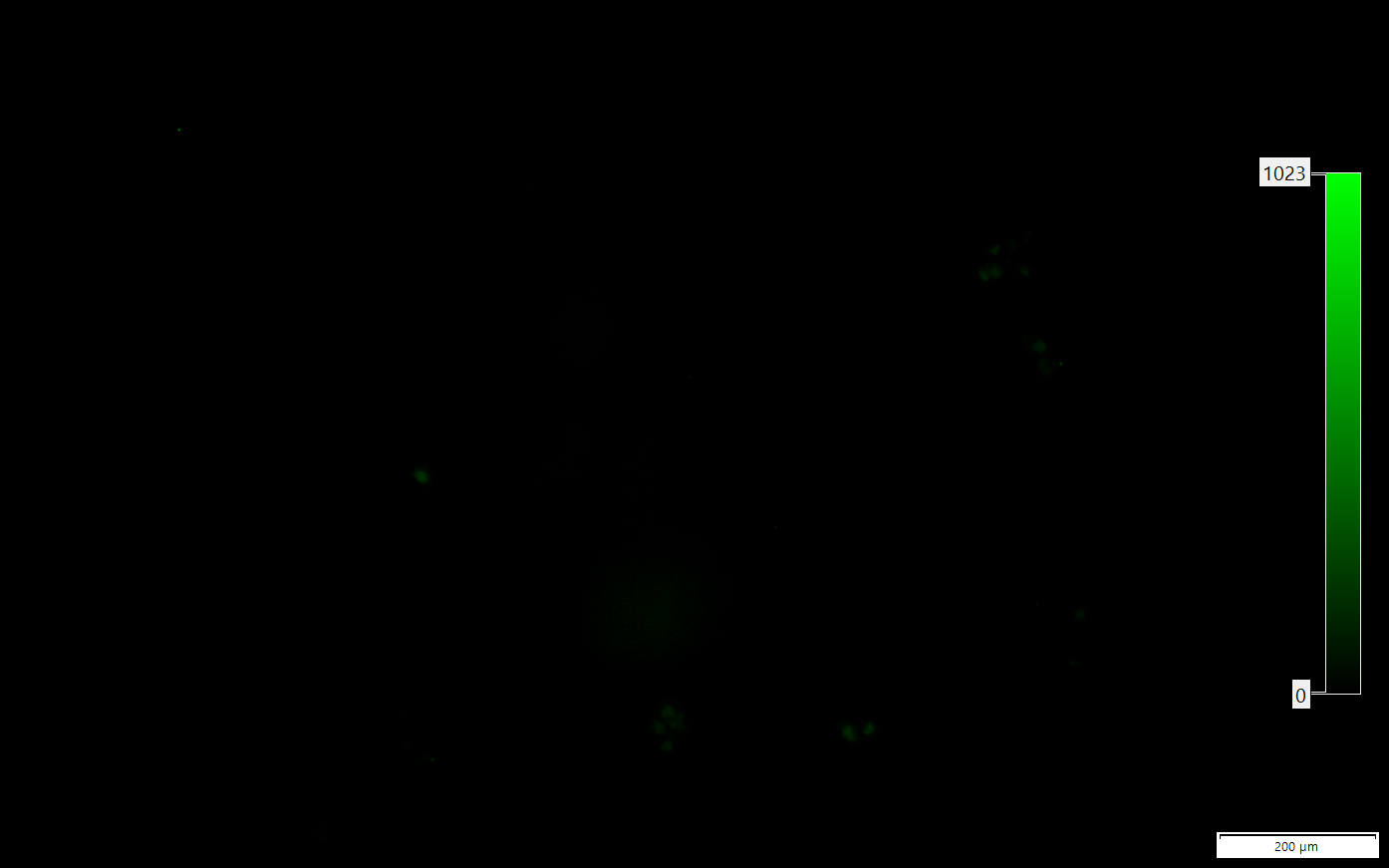

Supplement: Figure 5—source data 1. [file elife-85595-fig5-data1.zip › Figure 5-source data 1/Original_files/Figure 5A_WT_CTRL.tif]

Figure 5—figure supplement 3

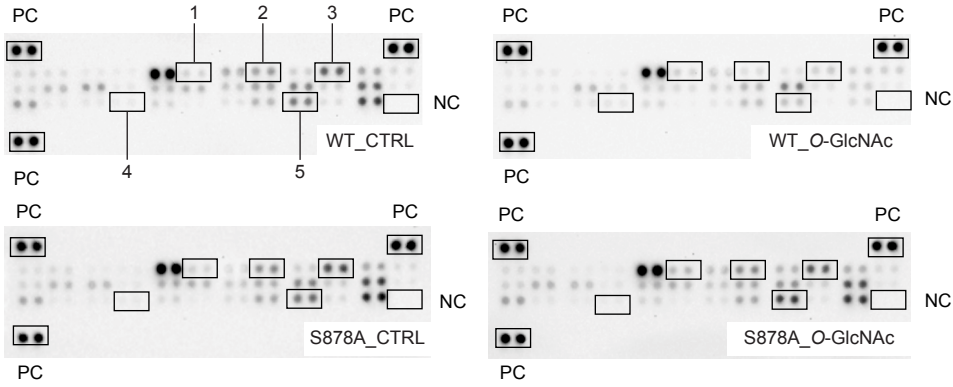

WT\_CTRL

WT\_O-GlcNAc

S878A\_CTRL

S878A\_O-GlcNAc

Supplement: Figure 5—figure supplement 3—source data 1. [file elife-85595-fig5-figsupp3-data1.zip › Figure 5-figure supplement 3-source data 1/Labeled_file/Figure 5-figure supplement 3-source data 1.pdf]

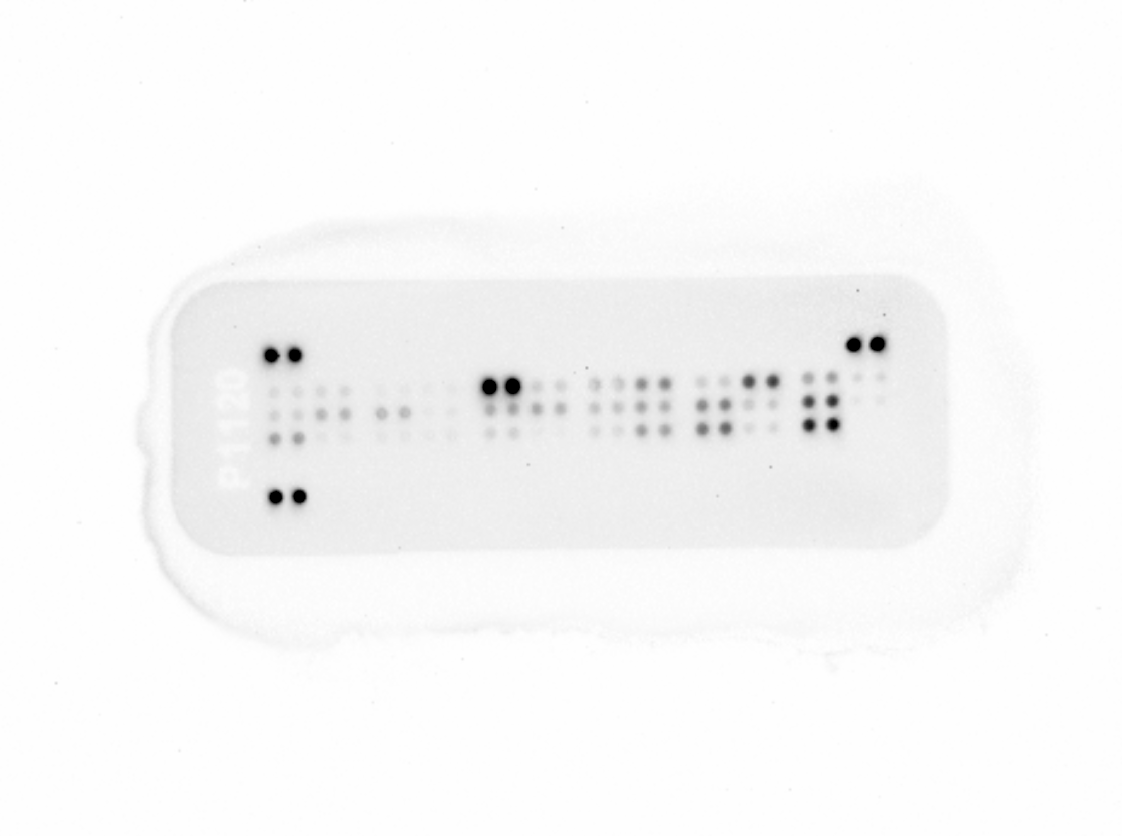

Supplement: Figure 5—figure supplement 3—source data 1. [file elife-85595-fig5-figsupp3-data1.zip › Figure 5-figure supplement 3-source data 1/Original_files/Figure 5-figure supplement 3_S878A_CTRL.tif]

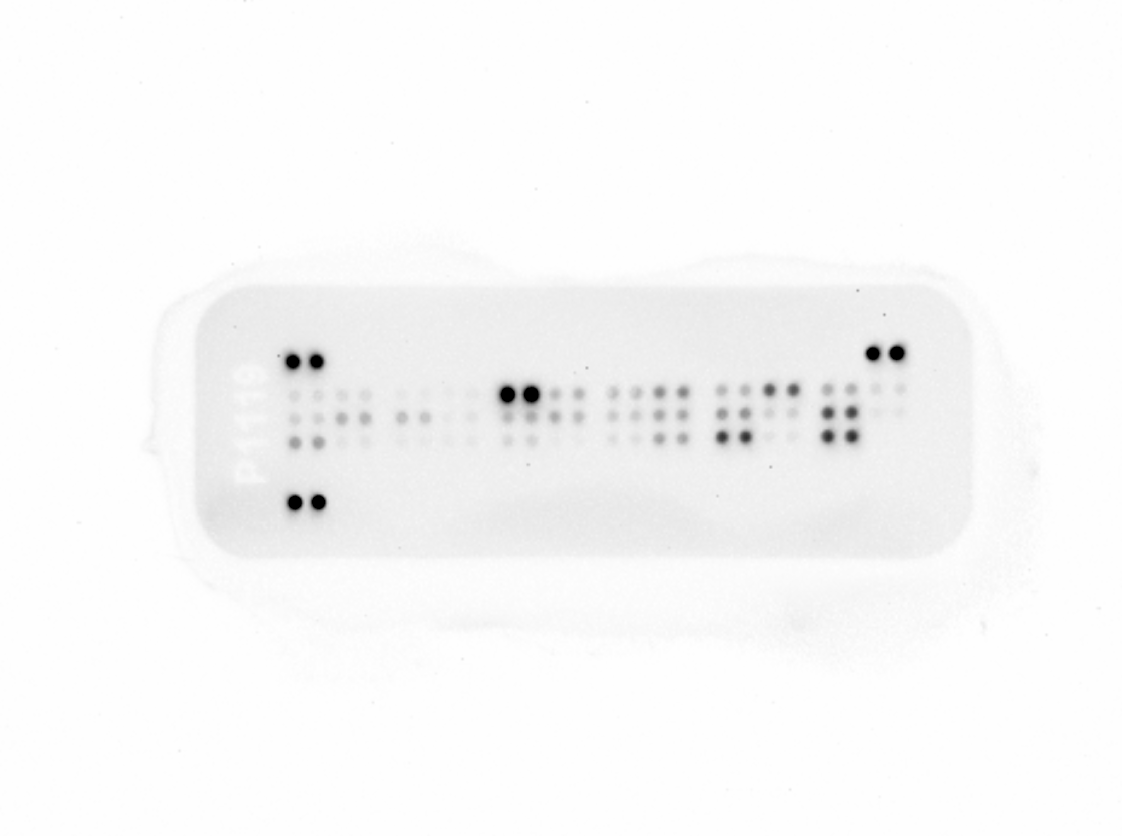

Supplement: Figure 5—figure supplement 3—source data 1. [file elife-85595-fig5-figsupp3-data1.zip › Figure 5-figure supplement 3-source data 1/Original_files/Figure 5-figure supplement 3_S878A_O-GlcNAc.tif]

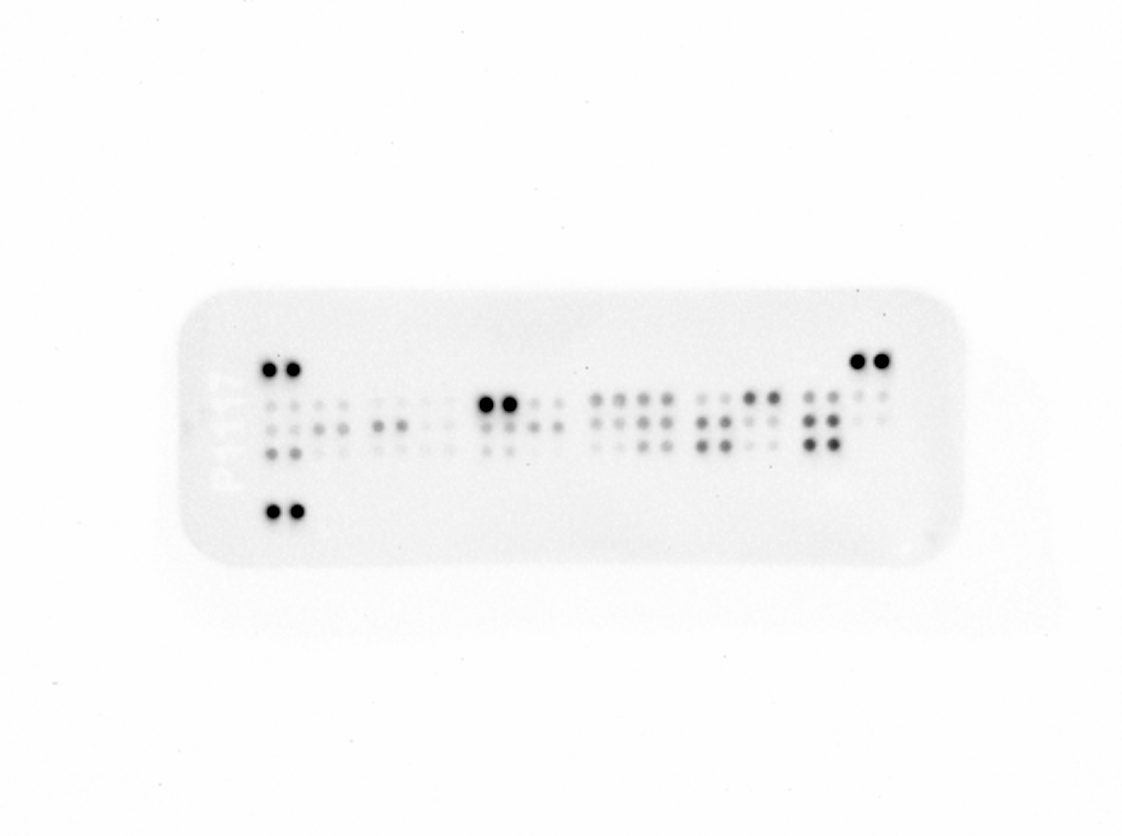

Supplement: Figure 5—figure supplement 3—source data 1. [file elife-85595-fig5-figsupp3-data1.zip › Figure 5-figure supplement 3-source data 1/Original_files/Figure 5-figure supplement 3_WT_CTRL.tif]

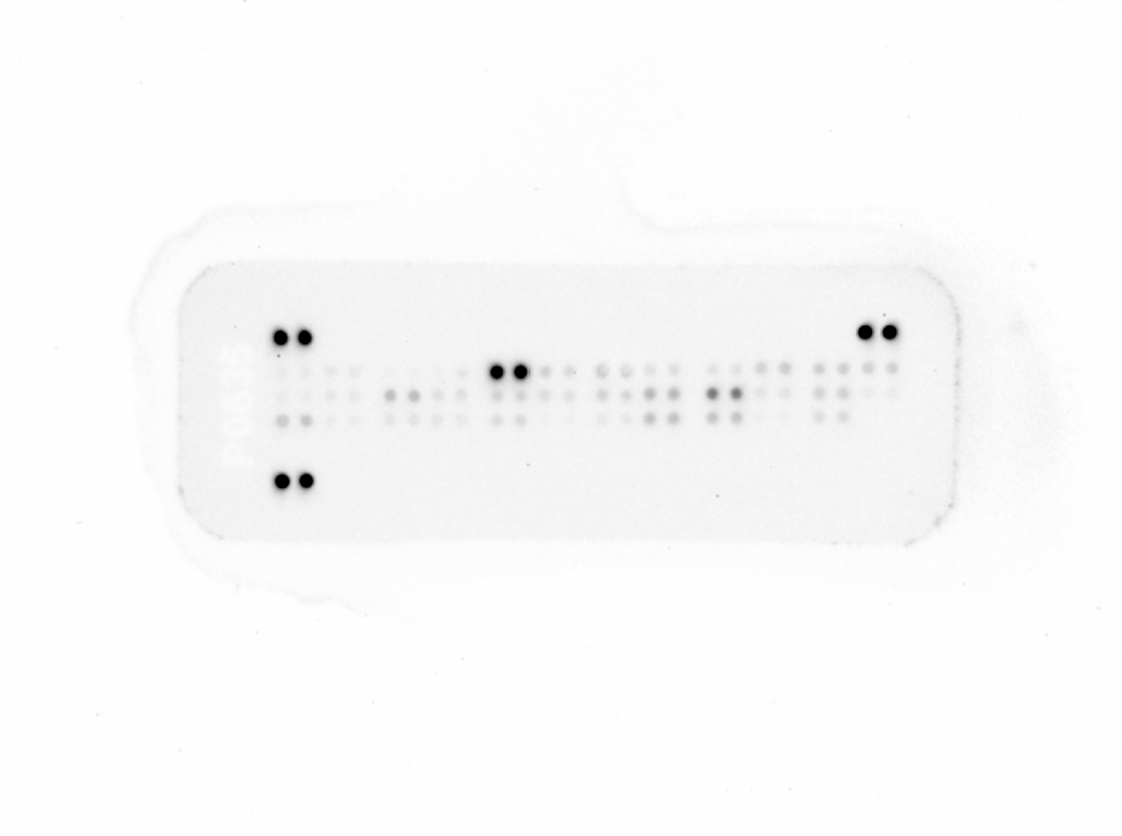

Supplement: Figure 5—figure supplement 3—source data 1. [file elife-85595-fig5-figsupp3-data1.zip › Figure 5-figure supplement 3-source data 1/Original_files/Figure 5-figure supplement 3_WT_O-GlcNAc.tif]
