# Supplementary material for: Inhibition of DNMT1 methyltransferase activity via glucose-regulated O-GlcNAcylation alters the epigenome
Source: eLife. 2023 Jul 20;12:e85595. doi: 10.7554/eLife.85595 (PMC10390045; doi:10.7554/eLife.85595)
Supplement: Supplementary file 1. [file elife-85595-supp1.docx]

**Supplementary File 1**

| **Protein name** | **Locations** | **Predicted *O*-GlcNAcylated sites** | **Score** |
| --- | --- | --- | --- |
| DNMT1 (P26358) | 5 | -MPAR T APARV | 0.8089 |
|  | 158 | PSPRI T RKSTR | 0.9368 |
|  | 161 | RITRK S TRQTT | 0.7442 |
|  | 162 | ITRKS T RQTTI | 0.7014 |
|  | 165 | KSTRQ T TITSH | 0.7822 |
|  | 166 | STRQT T ITSHF | 0.7471 |
|  | 168 | RQTTI T SHFAK | 0.6403 |
|  | 534 | NKIET T VPPSG | 0.7764 |
|  | 616 | KDRGP T KATTT | 0.8524 |
|  | 801 | WFCAG T DTVLG | 0.5094 |
|  | 882 | ESPPK T QPTED | 0.9432 |
|  | 895 | FKFCV S CARLA | 0.6618 |
|  | 977 | HYRKY S DYIKG | 0.3536 |
|  | 1034 | KSTPA S YHADI | 0.7661 |
|  | 1076 | CVQVY S MGGPN | 0.499 |
|  | 1122 | KGKPK S QACEP | 0.7928 |

**Supplementary File 1.** Prediction of *O*-GlcNAcylated sites within DNMT1 using OGTSite.
