# Supplementary material for: Inhibition of DNMT1 methyltransferase activity via glucose-regulated O-GlcNAcylation alters the epigenome
Source: eLife. 2023 Jul 20;12:e85595. doi: 10.7554/eLife.85595 (PMC10390045; doi:10.7554/eLife.85595)
Supplement: Supplementary file 2. [file elife-85595-supp2.docx]

**Supplementary File 2**

| **Gene**  **Symbol** | **Accession**  **[#]** | **Coverage [%]** | **Unique**  **Peptides**  **[#]** | **AAs**  **[#]** | **MW**  **[kDa]** | **calc. [pI]** | **Modifications** |
| --- | --- | --- | --- | --- | --- | --- | --- |
| DNMT1 | P26358 | 96 | 380 | 1616 | 183.1 | 7.75 | HexNAc [S878];  Phospho [T208; S209; S394; S398; S714; S953; S954; S1105; S1122] |
| PRMT5 | O14744 | 73 | 71 | 637 | 72.6 | 6.29 |  |
| PCNA | P12004 | 100 | 58 | 261 | 28.8 | 4.69 |  |
| USP7 | Q93009 | 38 | 43 | 1102 | 128.2 | 5.55 |  |
| HSPA1 | P0DMV9 | 60 | 42 | 641 | 70 | 5.66 |  |
| HSPA8 | P11142 | 57 | 40 | 646 | 70.9 | 5.52 |  |
| HSPD1 | P10809 | 57 | 34 | 573 | 61 | 5.87 |  |
| RBM10 | P98175 | 36 | 33 | 930 | 103.5 | 5.97 | Phospho [S554] |
| HSPA9 | P38646 | 50 | 29 | 679 | 73.6 | 6.16 |  |
| WDR77 | Q9BQA1 | 78 | 28 | 342 | 36.7 | 5.17 | Phospho [T5] |
| PPM1B | O75688 | 48 | 24 | 479 | 52.6 | 5.05 |  |
| EIF4B | P23588 | 43 | 19 | 611 | 69.1 | 5.73 | Phospho [S459] |
| OTUD4 | Q01804 | 19 | 16 | 1114 | 124 | 6.71 |  |
| TUBB | P07437 | 39 | 15 | 444 | 49.6 | 4.89 |  |
| STK38L | Q9Y2H1 | 36 | 13 | 464 | 54 | 6.81 | Phospho [S282] |
| TRIM21 | P19474 | 33 | 13 | 475 | 54.1 | 6.38 |  |
| HSPA5 | P11021 | 23 | 13 | 654 | 72.3 | 5.16 |  |
| KRT10 | P13645 | 19 | 13 | 584 | 58.8 | 5.21 |  |
| ERH | P84090 | 79 | 9 | 104 | 12.3 | 5.92 |  |
| RPL27A | P46776 | 49 | 9 | 148 | 16.6 | 11 |  |
| CLNS1A | P54105 | 44 | 9 | 237 | 26.2 | 4.11 | Phospho [S102] |
| PRPF31 | Q8WWY3 | 20 | 8 | 499 | 55.4 | 5.78 |  |
| PFKFB3 | Q16875 | 16 | 8 | 520 | 59.6 | 8.21 |  |
| KIF11 | P52732 | 11 | 8 | 1056 | 119.1 | 5.64 |  |
| SNRPD2 | P62316 | 81 | 7 | 118 | 13.5 | 9.91 |  |
| KRT1 | P04264 | 10 | 7 | 644 | 66 | 8.12 |  |
| MAP1B | P46821 | 4 | 7 | 2468 | 270.5 | 4.81 |  |
| MYCBP | Q99417 | 48 | 6 | 103 | 12 | 5.91 |  |
| SPIN1 | Q9Y657 | 24 | 6 | 262 | 29.6 | 6.96 |  |
| NPM1 | P06748 | 17 | 6 | 294 | 32.6 | 4.78 |  |
| KRT2 | P35908 | 13 | 6 | 639 | 65.4 | 8 |  |
| RPS11 | P62280 | 35 | 5 | 158 | 18.4 | 10.3 |  |
| HNRNPK | P61978 | 17 | 5 | 463 | 50.9 | 5.54 |  |
| STK38 | Q15208 | 13 | 5 | 465 | 54.2 | 7.15 | Phospho [S281] |
| KRT9 | P35527 | 12 | 5 | 623 | 62 | 5.24 |  |
| RIOK1 | Q9BRS2 | 12 | 5 | 568 | 65.5 | 6.19 |  |
| BCLAF1 | Q9NYF8 | 7 | 5 | 920 | 106.1 | 9.98 | Phospho [S512; S531] |
| TMPO | P42166 | 7 | 5 | 694 | 75.4 | 7.66 |  |
| QPCTL | Q9NXS2 | 15 | 4 | 382 | 42.9 | 9.82 |  |
| DBT | P11182 | 12 | 4 | 482 | 53.5 | 8.51 |  |
| TUFM | P49411 | 11 | 4 | 452 | 49.5 | 7.61 |  |
| SLC25A5 | P05141 | 11 | 4 | 298 | 32.8 | 9.69 |  |
| PABPC1 | P11940 | 5 | 4 | 636 | 70.6 | 9.5 |  |
| HYDIN | Q4G0P3 | 1 | 4 | 5121 | 575.5 | 6.06 |  |
| RPL29 | P47914 | 25 | 3 | 159 | 17.7 | 11.66 |  |
| RPS17 | P08708 | 21 | 3 | 135 | 15.5 | 9.85 |  |
| EEF1B2 | P24534 | 18 | 3 | 225 | 24.7 | 4.67 |  |
| C11orf84 | Q9BUA3 | 15 | 3 | 381 | 41 | 5.01 | Phospho [S308] |
| PRDX1 | Q06830 | 15 | 3 | 199 | 22.1 | 8.13 |  |
| RPS2 | P15880 | 14 | 3 | 293 | 31.3 | 10.24 |  |
| PSMD4 | P55036 | 12 | 3 | 377 | 40.7 | 4.79 |  |
| PSMC2 | P35998 | 10 | 3 | 433 | 48.6 | 5.95 |  |
| C1QBP | Q07021 | 9 | 3 | 282 | 31.3 | 4.84 |  |
| RPL38 | P63173 | 36 | 2 | 70 | 8.2 | 10.1 |  |
| SNRPD1 | P62314 | 18 | 2 | 119 | 13.3 | 11.56 |  |
| RPS25 | P62851 | 17 | 2 | 125 | 13.7 | 10.11 |  |
| PTS | Q03393 | 16 | 2 | 145 | 16.4 | 6.68 |  |
| RPS12 | P25398 | 15 | 2 | 132 | 14.5 | 7.21 |  |
| PRDX5 | P30044 | 14 | 2 | 214 | 22.1 | 8.7 |  |
| RPL13 | P26373 | 14 | 2 | 211 | 24.2 | 11.65 |  |
| RPS3 | P23396 | 12 | 2 | 243 | 26.7 | 9.66 |  |
| PSMD13 | Q9UNM6 | 8 | 2 | 376 | 42.9 | 5.81 |  |
| CAPZA1 | P52907 | 8 | 2 | 286 | 32.9 | 5.69 |  |
| YWHAB | P31946 | 8 | 2 | 246 | 28.1 | 4.83 |  |
| RPS24 | P62847 | 8 | 2 | 133 | 15.4 | 10.78 |  |
| PSMC4 | P43686 | 6 | 2 | 418 | 47.3 | 5.21 |  |
| CMBL | Q96DG6 | 6 | 2 | 245 | 28 | 7.18 |  |
| PSPC1 | Q8WXF1 | 6 | 2 | 523 | 58.7 | 6.67 |  |
| PTBP1 | P26599 | 5 | 2 | 531 | 57.2 | 9.17 |  |
| RPL11 | P62913 | 5 | 2 | 178 | 20.2 | 9.6 |  |
| PSMC1 | P62191 | 5 | 2 | 440 | 49.2 | 6.21 |  |
| P4HB | P07237 | 5 | 2 | 508 | 57.1 | 4.87 |  |
| PSMD12 | O00232 | 5 | 2 | 456 | 52.9 | 7.65 |  |
| HNRNPH1 | P31943 | 4 | 2 | 449 | 49.2 | 6.3 |  |
| CCT5 | P48643 | 4 | 2 | 541 | 59.6 | 5.66 |  |
| HSPH1 | Q92598 | 3 | 2 | 858 | 96.8 | 5.39 |  |
| BACE2 | Q9Y5Z0 | 3 | 2 | 518 | 56.1 | 5.15 |  |
| ADRB2 | P07550 | 3 | 2 | 413 | 46.4 | 7.03 |  |
| OSBPL1A | Q9BXW6 | 3 | 2 | 950 | 108.4 | 6.38 |  |
| FAM160A1 | Q05DH4 | 3 | 2 | 1040 | 116.5 | 4.86 |  |
| HSPA4L | O95757 | 3 | 2 | 839 | 94.5 | 5.88 |  |
| RNF219 | Q5W0B1 | 3 | 2 | 726 | 81.1 | 5.72 |  |
| THRAP3 | Q9Y2W1 | 2 | 2 | 955 | 108.6 | 10.15 |  |
| ITPRIP | Q8IWB1 | 2 | 2 | 547 | 62 | 5.88 |  |
| SPEF2 | Q9C093 | 1 | 2 | 1822 | 209.7 | 5.54 |  |
| ERCC6L | Q2NKX8 | 1 | 2 | 1250 | 141 | 5.31 |  |
| SRCAP | Q6ZRS2 | 1 | 2 | 3230 | 343.3 | 5.96 |  |

**Supplementary File 2.** List of total identified proteins.
