# Supplementary material for: Inhibition of DNMT1 methyltransferase activity via glucose-regulated O-GlcNAcylation alters the epigenome
Source: eLife. 2023 Jul 20;12:e85595. doi: 10.7554/eLife.85595 (PMC10390045; doi:10.7554/eLife.85595)
Supplement: Supplementary file 3. [file elife-85595-supp3.docx]

**Supplementary File 3**

| **Protein name** | **PTM** | **Locations** | **Newly identified** |
| --- | --- | --- | --- |
| DNMT1 (P26358) | Glycyl lysine isopeptide | 259, 1609 | - |
|  | N6-acetyllysine | 160, 173, 188, 259, 366, 749, 891, 957, 961, 975, 1054, 1111, 1113, 1115, 1117, 1119, 1121, 1349, 1415 | - |
|  | N6,N6-dimethyllysine | 70 | - |
|  | N6-methyllysine | 142 | - |
|  | *O*-GlcNAc | 878 | S878 |
|  | Phosphothreonine | 137, 166, 208 | T208 |
|  | Phosphotyrosine | 399, 969 | - |
|  | Phosphoserine | 35, 127, 133, 141, 143, 152, 154, 189, 192, 209, 312, 394, 398, 509, 549, 714, 732, 878, 953, 954, 1105, 1122 | S209, S1122 |

**Supplementary File 3.** List of posttranslational modification (PTM) sites of human DNMT1.
