# Supplementary material for: Inhibition of DNMT1 methyltransferase activity via glucose-regulated O-GlcNAcylation alters the epigenome
Source: eLife. 2023 Jul 20;12:e85595. doi: 10.7554/eLife.85595 (PMC10390045; doi:10.7554/eLife.85595)
Supplement: Supplementary file 4. [file elife-85595-supp4.docx]

**Supplementary File 4**

| **Deposited data** | | |
| --- | --- | --- |
| DNMT1-WT-CTRL | This paper | GEO: GSE201470 |
| DNMT1-WT-*O*-GlcNAc | This paper | GEO: GSE201470 |
| DNMT1-S878A-CTRL | This paper | GEO: GSE201470 |
| DNMT1-S878A-*O*-GlcNAc | This paper | GEO: GSE201470 |
| Mass spectrometry proteomics data | This paper | PXD043031 |
| Partially methylated domains of liver cancer | Li et al., 2016 | GEO: GSE70091 |
| RNA sequencing | Chang et al., 2014 | GEO: GSE49994 |

**Supplementary File 4.** List of deposited data in this study.
